# A Bayesian multivariate factor analysis model for causal inference using time-series observational data on mixed outcomes

**DOI:** 10.1093/biostatistics/kxad030

**Published:** 2023-12-06

**Authors:** Pantelis Samartsidis, Shaun R Seaman, Abbie Harrison, Angelos Alexopoulos, Gareth J Hughes, Christopher Rawlinson, Charlotte Anderson, André Charlett, Isabel Oliver, Daniela De Angelis

**Affiliations:** MRC Biostatistics Unit, East Forvie Building, Cambridge Biomedical Campus, Cambridge, CB2 0SR, UK; MRC Biostatistics Unit, East Forvie Building, Cambridge Biomedical Campus, Cambridge, CB2 0SR, UK; UK Health Security Agency, London, E14 4PU, UK; MRC Biostatistics Unit, East Forvie Building, Cambridge Biomedical Campus, Cambridge, CB2 0SR, UK; Department of Economics, Athens University of Economics and Business, Athens, 104 34, Greece; UK Health Security Agency, London, E14 4PU, UK; UK Health Security Agency, London, E14 4PU, UK; UK Health Security Agency, London, E14 4PU, UK; UK Health Security Agency, London, E14 4PU, UK; UK Health Security Agency, London, E14 4PU, UK; MRC Biostatistics Unit, East Forvie Building, Cambridge Biomedical Campus, Cambridge, CB2 0SR, UK; UK Health Security Agency, London, E14 4PU, UK

**Keywords:** Causal inference, Contact tracing, Data augmentation, Factor analysis, Policy evaluation

## Abstract

Assessing the impact of an intervention by using time-series observational data on multiple units and outcomes is a frequent problem in many fields of scientific research. Here, we propose a novel Bayesian multivariate factor analysis model for estimating intervention effects in such settings and develop an efficient Markov chain Monte Carlo algorithm to sample from the high-dimensional and nontractable posterior of interest. The proposed method is one of the few that can simultaneously deal with outcomes of mixed type (continuous, binomial, count), increase efficiency in the estimates of the causal effects by jointly modeling multiple outcomes affected by the intervention, and easily provide uncertainty quantification for all causal estimands of interest. Using the proposed approach, we evaluate the impact that Local Tracing Partnerships had on the effectiveness of England’s Test and Trace programme for COVID-19.

## 1 Introduction

This article considers the problem of estimating from observational data the causal effect of an intervention (or treatment/policy) on an outcome of interest in applications where: (i) there are multiple sample units, some of which receive the intervention; (ii) for each unit, the outcome of interest is measured at multiple time points; and (iii) the units receiving the intervention and the times at which the intervention is implemented are not randomized. This problem arises often in epidemiology (Barnard *et al.*, 2022), public health (Bruhn *et al.*, 2017), economics (Hsiao *et al.*, 2012), marketing (Brodersen *et al.*, 2015), political science (Xu, 2017), and social studies (Ben-Michael *et al.*, 2023). Often the sample units in such applications represent groups of individuals; for example, the units might be hospitals or administrative regions. See Ferman *et al.* (2020) and Samartsidis *et al.* (2019) for a list of real-world examples.

Methods for causal evaluation in settings where (i)–(iii) apply can be broadly classified as *causal factor analysis* (or “matrix completion”) (Kim and Oka, 2014; Gobillon and Magnac, 2016; Xu, 2017; Athey *et al.*, 2021; Nethery *et al.*, 2023; Samartsidis *et al.*, 2020; Pang *et al.*, 2021, among others) or *synthetic control* (Abadie *et al.*, 2010; Hsiao *et al.*, 2012; Brodersen *et al.*, 2015; Robbins *et al.*, 2017; Ben-Michael *et al.*, 2021, among others) approaches. For an overview of these methods and an explanation of how they account for potential confounding, see Clarke *et al.* (2023), Xu (2023), and Samartsidis *et al.* (2019).

Existing approaches have limitations. First, for some of them, it is hard to obtain uncertainty intervals for the causal effects of interest. Second, most of them are designed to deal with continuous outcomes. An exception is Nethery *et al.* (2023), who develop a factor model for a single count outcome. However, these authors do not consider binomial outcomes, which arise frequently in applications; nor do they account for uncertainty in the number of latent factors. Third, few of these methods allow joint modeling of multiple outcomes affected by the intervention. Such joint modeling may improve statistical efficiency, which is particularly valuable when evaluating policies in contexts where data are sparse, i.e. only available annually or quarterly over a limited number of years. In recent work (Samartsidis *et al.*, 2020), we used the multivariate factor analysis model of De Vito *et al.* (2021) to jointly model multiple normally distributed outcomes. However, this model cannot be applied to binomial/count data. It also assumes that variability shared across any of the multiple outcomes is shared across all the multiple outcomes.

Here, we develop a general approach to causal inference from time-series observational data that tackles these limitations. This method uses a multivariate factor analysis model that can be applied to multiple outcomes of mixed type (continuous/binomial/count) and allows for sharing of the loadings parameters across these outcomes. The model is Bayesian and thus automatically provides uncertainty quantification for all quantities of interest. Our model accounts for uncertainty in the number of latent factors and allows sharing of loadings across any subsets of the multiple outcomes. The Markov chain Monte Carlo (MCMC) algorithm we propose for fitting the model combines modern data augmentation and sampling techniques to overcome slow mixing issues stemming from the well-known identifiability problems of factor analysis models. The potential benefits of joint outcome modeling are demonstrated via a simulation study. We apply the proposed method to real data on a new policy for COVID-19 contact tracing in UK, providing valuable insights on the effectiveness of this policy. Finally, although our model is developed for intervention evaluation problems, it can be of use in other fields where multivariate factor analysis models are of interest, such as cancer biology (Avalos-Pacheco *et al.*, 2022).

The remainder is structured as follows. In Section 2, we introduce the motivating COVID-19 contact tracing policy evaluation problem. Section 3 presents our Bayesian model. In Section 4, we discuss model fitting. Section 5 presents a simulation study that demonstrates the benefit of jointly modeling multiple outcomes. Section 6 contains results from our motivating data set. Finally, Section 7 summarizes the article’s findings and lists some directions for future research.

## 2 COVID-19 contact tracing policy evaluation problem

The NHS *Test & Trace* (TT) programme was launched in May 2020 as part of the containment strategy for the COVID-19 pandemic in UK. One of its main functions was to ensure that individuals who tested positive for COVID-19 (the *cases*) were aware^1^ of their infection, and to inform them of their legal duty to self-isolate to prevent onward transmission. When TT communicated with a case (henceforth this communication will be referred to as *tracing*), the case was asked to list the individuals (*contacts*) with whom he/she had been in close physical proximity within 48 h of his/her symptom onset (for asymptomatic cases, date of positive test is used). TT would then attempt to reach these contacts to provide advice and tell them to self-isolate. Multiple studies have provided evidence in favor of TT (Fetzer and Graeber, 2021).

Starting in July 2020, local authorities gradually introduced *local tracing partnerships* (LTPs). These LTPs involved the local authority working with national TT to improve tracing of cases and contacts. An LTP team was composed of staff based locally, whose job was to trace cases resident in that local authority who had not been able to be traced by TT. This is done mainly by telephone, often using the team’s local knowledge and, in several authorities, by visiting a case’s home if telephone communication had not been successful. The question we address here is whether LTPs (the *intervention*) have had an impact on the effectiveness of TT. Which local authorities introduced LTPs, and when, depended mainly on the interest expressed by local authorities, rather than being randomized, which complicates estimation of the causal effects of LTPs.

We judge the effectiveness of TT in terms of four outcomes measured daily: (i) the proportion of cases on that day that were ultimately completed, where a case is considered *complete* if TT manage to contact him/her; (ii) the proportion of completed cases whose completion was timely, i.e., occurs within 48 h of the case being registered with TT; (iii) the total number of contacts elicited from cases completed on that day; and (iv) the proportion of these elicited contacts who were subsequently completed. We shall refer to these as case completion, timely case completion, number of contacts and contact completion, respectively. We have daily measurements of these four outcomes on 181 spatial units during the study period July 01, 2020 to November 15, 2020 (i.e., 138 days). Each of these units is either an upper-tier local authority (UTLA) or part of a UTLA.

**Fig. 1:**
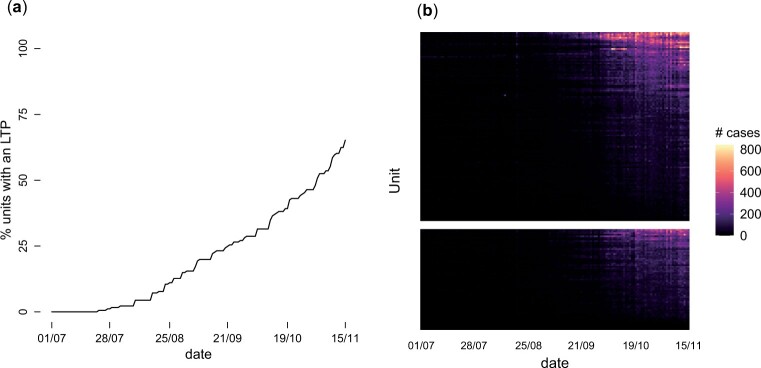
Graphical summaries of the data. (a) Proportion of units (out of 181) that had already formed an LTP by each day. (b) Total number of new cases for each day and unit, where units have been ordered by average number of new cases.

Figure 1 provides some graphical summaries of the data. The roll-out of LTPs is summarized in Fig. 1(a). The first LTP was formed on July 15, 2020 and by November 15, 2020, 118 (66%) of the units were covered by an LTP. Figure 1(b) shows the number of cases for each day and unit; during July–September the number of cases is low for most of the units, but it increases thereafter. The data on the four outcomes (Fig. D.1 of the supplementary material available at *Biostatistics* online) show considerable variability across units for the same day and across time within the same unit. The existing methods for policy evaluation listed in Section 1 cannot be used to investigate whether LTPs improved some of these outcomes because they are not applicable to binomial data.

## 3 Methods for estimation of causal effects

### 3.1 Latent factor model and causal effects

Let i=1,…,N index the sample unit and t=1,…T denote time (e.g., in days) relative to a fixed calendar time (e.g., July 1st 2020 in our application). Let *N*_1_ and $N_{2}=N-N_{1}$ be the numbers of *control units* and *treated units*, respectively (i.e., units that do not and units that do experience the intervention). Without loss of generality, assume units are ordered so that $1,\ldots ,N_{1}$ are control units. Let *T_i_* denote the last time at which unit *i* has not experienced the intervention (for control units, *T_i_* = *T*). Let $T_{\text{min}}=\min\{T_{1},\ldots T_{N}\}$ and ${\tilde{N}}_{t}=\sum_{i=N_{1}+1}^{N}I(T_{i}<t)$.

Suppose there are *D*_1_ continuous, *D*_2_ binomial and *D*_3_ count outcomes. Let *y_itd_* be the observed value of the *d*th continuous outcome ($d=1,\ldots ,D_{1}$). For the *d*th binomial outcome ($d=1,\ldots ,D_{2}$), let *k_itd_* be the observed number of successes in a known number, *n_itd_*, of independent Bernoulli trials. Let *z_itd_* be the observed value of the *d*th count outcome ($d=1,\ldots ,D_{3}$).

We shall define the causal effect of the intervention on any one of the $D=D_{1}+D_{2}+D_{3}$ outcomes in unit *i* at time *t* in terms of the difference between its observed value and the outcome that unit *i* would have if it were not treated at time *t*. The latter value is called a *potential untreated outcome* (Holland, 1986). We denote the potential untreated continuous, binomial, and count outcomes as $y_{\mathrm{itd}}^{\left(0\right)},\;k_{\mathrm{itd}}^{\left(0\right)}$, and $z_{\mathrm{itd}}^{\left(0\right)}$, respectively. We also define the *potential treated outcomes* $y_{\mathrm{itd}}^{\left(s\right)},\;k_{\mathrm{itd}}^{\left(s\right)}$, and $z_{\mathrm{itd}}^{\left(s\right)}$ ($s=1,\ldots T_{i}$) as the outcomes that unit *i* would have at time *t* if time *s* had been the last time at which unit *i* had not experienced the intervention. We make the stable unit treatment value assumption (SUTVA), which implies that the potential outcomes of one unit do not depend on whether, or at what time, the intervention is applied to other units. We further make the no-treatment-anticipation assumption (Athey and Imbens, 2021) which means that $y_{\mathrm{itd}}^{\left(s\right)}=y_{\mathrm{itd}}^{\left(0\right)},\;k_{\mathrm{itd}}^{\left(s\right)}=k_{\mathrm{itd}}^{\left(0\right)}$, and $z_{\mathrm{itd}}^{\left(s\right)}=z_{\mathrm{itd}}^{\left(0\right)}$ for all t≤s. With these assumptions, the observed outcomes are related to the potential outcomes by $y_{\mathrm{itd}}=y_{\mathrm{itd}}^{\left(0\right)},\;k_{\mathrm{itd}}=k_{\mathrm{itd}}^{\left(0\right)}$, and $z_{\mathrm{itd}}=z_{\mathrm{itd}}^{\left(0\right)}$ if $t\leq T_{i}$; and $y_{\mathrm{itd}}=y_{\mathrm{itd}}^{\left(T_{i}\right)},\;k_{\mathrm{itd}}=k_{\mathrm{itd}}^{\left(T_{i}\right)}$, and $z_{\mathrm{itd}}=z_{\mathrm{itd}}^{\left(T_{i}\right)}$ if $t>T_{i}$.

There is a growing interest in using *factor analysis* (FA) to model the potential untreated outcomes (see Section 1 for references), or as the data generating mechanism to study the properties of synthetic control-type approaches (Abadie *et al.*, 2010; Hsiao *et al.*, 2012; Ben-Michael *et al.*, 2021; Ferman, 2021). This is due to the FA model’s ability to adjust for observed covariates and to allow for the presence of unmeasured confounding with a particular structure. Here, we propose a multivariate, mixed-outcome FA model for the potential untreated outcomes. Specifically, for each i=1,…,N and t=1,…,T, we assume

(3.1) $$\begin{array}{c} y_{\mathrm{itd}}^{\left(0\right)}∼N\left(\mu_{\mathrm{itd}},\sigma_{id}^{2}\right),\mu_{\mathrm{itd}}=\lambda_{i}^{⊤}f_{td}+\eta_{1,d}^{⊤}x_{it},(d=1,\ldots ,D_{1})\\ k_{\mathrm{itd}}^{\left(0\right)}∼\text{Bin}\left(n_{\mathrm{itd}},p_{\mathrm{itd}}\right),\text{logit}(p_{\mathrm{itd}})=\lambda_{i}^{⊤}g_{td}+\eta_{2,d}^{⊤}x_{it},(d=1,\ldots ,D_{2})\\ z_{\mathrm{itd}}^{\left(0\right)}∼\text{NegBin}\left(w_{\mathrm{itd}}q_{\mathrm{itd}}\xi_{d}^{-1},{\left(1+\xi_{d}\right)}^{-1}\right),\;\text{log}(q_{\mathrm{itd}})=\lambda_{i}^{⊤}h_{td}+\eta_{3,d}^{⊤}x_{it}.(d=1,\ldots ,D_{3}). \end{array}$$

Here, $f_{td},g_{td},h_{td}$ are *J*-vectors of unobserved factors at time *t*; $\lambda_{i}$ is an unobserved *J*-vector of loadings for unit *i*; $x_{it}$ is a *P*-vector of (possibly time-dependent) exogenous (not affected by the intervention) covariates; and $\eta_{\ell ,d}$ are its regression coefficients (ℓ=1,2,3; $d=1,\ldots ,D_{\ell }$). NegBin(a,b) denotes the negative binomial distribution with mean a(1−b)/b and variance $a(1-b)/b^{2}$. It follows from (3.1) that $E(z_{\mathrm{itd}})=w_{\mathrm{itd}}q_{\mathrm{itd}}$ and $V\text{ar}(z_{\mathrm{itd}})=w_{\mathrm{itd}}q_{\mathrm{itd}}(1+\xi_{d})$. So, $\xi_{d}>0$ is the degree of overdispersion relative to the Poisson distribution. Here, *w_itd_* is a known offset; if there is no offset, *w_itd_* = 1. In some applications, there might be enough information to estimate a separate dispersion for each unit; in such cases, we can replace *ξ_d_* by *ξ_id_*.

We assume that. for all i=1,…,N,

$$T_{i}⊥⊥\{(y_{it1}^{\left(0\right)},\ldots ,y_{itD_{1}}^{\left(0\right)},k_{it1}^{\left(0\right)},\ldots ,k_{itD_{2}}^{\left(0\right)},z_{it1}^{\left(0\right)},\ldots ,z_{itD_{3}}^{\left(0\right)}):\;t=1,\ldots ,T\}|x_{i1},\ldots ,x_{iT},\lambda_{i}.$$

This implies that after controlling for observed potential confounders $x_{i1},\ldots ,x_{iT}$ and unobserved potential confounders $\lambda_{i}$, there is no confounding of the causal effects of the intervention on the *d*th continuous, *d*th binary and *d*th count outcome of unit $i>N_{1}$ at time $t>T_{i}$, defined as

(3.2) $$\begin{array}{c} \alpha_{\mathrm{itd}}=y_{\mathrm{itd}}^{\left(T_{i}\right)}-y_{\mathrm{itd}}^{\left(0\right)}=y_{\mathrm{itd}}-y_{\mathrm{itd}}^{\left(0\right)},\;\gamma_{\mathrm{itd}}=k_{\mathrm{itd}}^{\left(T_{i}\right)}-k_{\mathrm{itd}}^{\left(0\right)}=k_{\mathrm{itd}}-k_{\mathrm{itd}}^{\left(0\right)},\;\delta_{\mathrm{itd}}=z_{\mathrm{itd}}^{\left(T_{i}\right)}\\ \;-z_{\mathrm{itd}}^{\left(0\right)}=z_{\mathrm{itd}}-z_{\mathrm{itd}}^{\left(0\right)}, \end{array}$$

respectively. An alternative to *γ_itd_* would be to define the causal effect on the *d*th binomial outcome of unit *i* at time *t* in terms of the success probability of the Bernoulli trials. To do this, we would assume $k_{\mathrm{itd}}^{\left(T_{i}\right)}=k_{\mathrm{itd}}∼\text{Bin}\left(n_{\mathrm{itd}},\;p_{\mathrm{itd}}^{\left(T_{i}\right)}\right)$ (for $i>N_{1},\;t>T_{i}$) and define the causal effect as

(3.3) $$\beta_{\mathrm{itd}}=p_{\mathrm{itd}}^{\left(T_{i}\right)}-p_{\mathrm{itd}}.$$

For the *d*th continuous outcome, we define (with slight abuse of notation) the average (over time) causal effect in unit *i* as $\alpha_{id}=\sum_{t=T_{i}+1}^{T}\alpha_{\mathrm{itd}}/(T-T_{i})$ and the overall (over both time and units) causal effect as $\alpha_{d}=(\sum_{i=N_{1}+1}^{N}\sum_{t=T_{i}+1}^{T}\alpha_{\mathrm{itd}})/(\sum_{t=1}^{T}{\tilde{N}}_{t})$. Corresponding average causal effects, *β_id_*, *β_d_*, *γ_id_*, *γ_d_*, *δ_id_*, and *δ_d_*, for binary and count outcomes are defined analogously.

Policy makers are also interested in identifying treated units whose response to the intervention (i.e., causal effect) is extreme compared to other treated units. This could be useful, for example, to identify units to which alternative interventions should be applied. For the *d*th continuous outcome, unit *i* and time $t>T_{i}$, define $r_{\mathrm{itd}}^{\left(\alpha \right)}$ as the (scaled) rank of the *α_itd_* values of the ${\tilde{N}}_{t}$ units treated by time *t*, i.e.,

(3.4) $$r_{\mathrm{itd}}^{\left(\alpha \right)}=\frac{\sum_{j:T_{j}<t}I(\alpha_{\mathrm{jtd}}\leq \alpha_{\mathrm{itd}})}{{\tilde{N}}_{t}+1}.$$

In (3.4), we have scaled by ${\left({\tilde{N}}_{t}+1\right)}^{-1}$ to ensure that ranks are comparable between any times *t* and *s* (both $>T_{\text{min}}$) for which ${\tilde{N}}_{t}\neq {\tilde{N}}_{s}$. Further, let $r_{id}^{\left(\alpha \right)}=\sum_{t=T_{i}+1}^{T}r_{\mathrm{itd}}^{\left(\alpha \right)}/(T-T_{i})$ be the average (over time) rank of unit *i*. Ranks for *β*, *γ*, and *δ* are defined analogously.

### 3.2 LTP application

In our motivating problem, where intervention is the introduction of an LTP, *N* = 181, *T* = 138, $D_{1}=0$ (no continuous outcomes), $D_{2}=3$ (case completion, timely case completion and contact completion), $D_{3}=1$ (number of contacts), and $N_{1}=63$ units have no LTP during study period.

For the first binary outcome (case completion), $n_{it1}$ is the number of cases registered on day *t* for unit *i*, and $k_{it1}$ is the number of these cases that were ultimately completed. For the second binary outcome (timely case completion), $n_{it2}$ is the number of cases completed on day *t* for unit *i*, and $k_{it2}$ is the number of these cases that were completed within 48 h. For the count outcome (number of contacts), $w_{it1}$ is the number of cases that were completed on day *t* for unit *i*, and $z_{it1}$ is the number of contacts elicited from these $w_{it1}$ cases. For the third binary outcome (contact completion), $k_{it3}$ is the number of contacts that were ultimately completed from the $n_{it3}=z_{it1}$ contacts. No covariates $x_{it}$ are considered.

In Section 3.1, we assumed *n_itd_* and *w_itd_* are not affected by the intervention. In the LTP application, however, $n_{it2}=k_{it1}$ and $n_{it3}=z_{it1}$, which may both be affected. Also, $w_{it1}$ is related to $k_{ij1}$ and $k_{ij2}$ for j≤t, and so may be affected. It is also possible that $n_{it1}$ is affected (successful tracing can prevent new cases). This changes the interpretation of the causal effects $\gamma_{it1},\;\gamma_{it2},\;\gamma_{it3}$, and $\delta_{it1}$. As shown in Section A of the supplementary material available at *Biostatistics* online, they can be interpreted (under some additional assumptions) as separable direct effects on $k_{it1},\;k_{it2},\;k_{it3}$, and $z_{it1}$, respectively. These are different from the corresponding *total* effects, defined as ${\tilde{\gamma }}_{\mathrm{itd}}=k_{\mathrm{itd}}-{\tilde{k}}_{\mathrm{itd}}^{\left(0\right)}$ and ${\tilde{\delta }}_{\mathrm{itd}}=z_{\mathrm{itd}}-{\tilde{z}}_{\mathrm{itd}}^{\left(0\right)}$, respectively, where ${\tilde{k}}_{\mathrm{itd}}^{\left(0\right)}$ and ${\tilde{z}}_{\mathrm{itd}}^{\left(0\right)}$ are obtained by replacing *n_itd_* and *w_itd_* with their potential untreated outcomes in (3.1). We do not consider the total effects (even though they can be estimated), because we believe they can be misleading. For example, suppose that for some $i>N_{1}$ and $t>T_{i},\;p_{it3}^{\left(T_{i}\right)}=p_{it3}$ and $n_{it3}>{\tilde{n}}_{it3}$, that is, LTP has no impact on contact completion probability but increases the number of contacts elicited. Then $k_{it3}>{\tilde{k}}_{it3}^{\left(0\right)}$ (because larger number of trials), and hence ${\tilde{\gamma }}_{it3}>0$, suggesting that the LTP increased the number of contacts completed. In contrast, $\gamma_{it3}$, interpreted as the total number of additional contacts completed thanks to the LTP, would be zero.

### 3.3 Outline of inference

In order to estimate the causal effects defined in Section 3.1, we need to estimate the potential untreated outcomes of the treated units $i>N_{1}$ in their postintervention periods $t>T_{i}$. We do this under the Bayesian paradigm as follows.

First, we set prior distributions for the parameters of the multivariate FA model (3.1). These are detailed in Section 3.4. We then fit, using the MCMC algorithm in Section 4, the FA model to pre-intervention data only (i.e., for each *i*, we only use data up to time *T_i_*). The posterior samples that we obtain allow us to draw samples from the posterior predictive distribution of the potential untreated outcomes conditional on $x_{it}$, *n_itd_* and *w_itd_*. We transform them using (3.2), to obtain samples from the posterior distribution of causal effects *α_itd_*, *γ_itd_*, and *δ_itd_*. For *β_itd_*, we further need to account for uncertainty in $p_{\mathrm{itd}}^{\left(T_{i}\right)}$. For each $i>N_{1},\;t>T_{i}$, and $d=1,\ldots ,D_{2}$, we *a priori* assume that $p_{\mathrm{itd}}^{\left(T_{i}\right)}∼\text{Beta}(a_{p},b_{p})$. Thus, conditional on postintervention data, $p_{\mathrm{itd}}^{\left(T_{i}\right)}∼\text{Beta}(a_{p}+k_{\mathrm{itd}},b_{p}+n_{\mathrm{itd}}-k_{\mathrm{itd}})$. We obtain samples from the posterior of *β_itd_* using (3.3), i.e., by subtracting the draws from the posterior of *p_itd_* from the samples obtained from the beta posterior of $p_{\mathrm{itd}}^{\left(T_{i}\right)}$.

Let $\alpha_{\mathrm{itd}}^{\left(\ell \right)}$ denote the ℓth sample (ℓ=1,…,L) from the posterior distribution of *α_itd_*. We estimate *α_itd_* as $\sum_{\ell =1}^{L}\alpha_{\mathrm{itd}}^{\left(\ell \right)}/L$ (the posterior mean) and calculate 95% posterior credible intervals (CIs) using the 2.5% and 97.5% percentiles of the $\alpha_{\mathrm{itd}}^{\left(\ell \right)}$. Samples from the posterior (and hence point estimates and 95% CIs) of *α_id_*, *α_td_*, *α_d_*, and $r_{\mathrm{itd}}^{\left(\alpha \right)}$ can be obtained from the $\alpha_{\mathrm{itd}}^{\left(\ell \right)}$ using the expressions provided in Section 3.1. For binomial and count outcomes, point estimates and 95% CIs are obtained analogously.

### 3.4 Prior distributions

One of the main challenges in fitting the FA model (3.1) is that the number of latent loadings *J* is not known. To account for uncertainty in *J*, we start by setting it to $J^{*}$, an upper limit for the number of factors we expect. Following Gao *et al.* (2016), we assign a three-level three-parameter beta prior to the loadings. In particular, for $j=1,\ldots ,J^{*}$ we let

(3.5) $$\begin{array}{c} \;\;\lambda_{ij}∼N(0,\frac{1}{\phi_{ij}}-1),\;\phi_{ij}∼\text{TPB}(a_{\lambda },b_{\lambda },\frac{1}{\zeta_{j}}-1),\\ \zeta_{j}∼\text{TPB}(c_{\lambda },d_{\lambda },\frac{1}{\rho }-1),\;\rho ∼\text{TPB}(e_{\lambda },f_{\lambda },\nu ), \end{array}$$

where x∼TPB(a,b,c) means that x∈(0,1) has density $\pi (x|a,b,c)=\frac{\Gamma (a+b)}{\Gamma (a)\Gamma (b)}c^{b}x^{b-1}{\left(1-x\right)}^{a-1}{\left(1+(c-1)x\right)}^{-a-b}$. Following Zhao *et al.* (2016), we set $a_{\lambda },\ldots ,f_{\lambda }=0.5$ and ν=0.1.

Let Λ be the $N\times J^{*}$ matrix with rows $\lambda_{i}$. The prior (3.5) induces three levels of regularization in Λ (Zhao *et al.*, 2016). The overall matrix shrinkage is controlled by *ρ*. The *ζ_j_* induce loading-specific shrinkage. When *ζ_s_* is estimated as ≈1 for some $1\leq s\leq J^{*}$, we will also have that $\lambda_{is}\approx 0$ for all *i*, thus effectively removing the *s*th column of Λ. This allows us to recover the effective number of factors supported by the data as the number of nonzero^2^ columns in Λ. Finally, the $\phi_{ij}$ allow the scale of the *j*th loading to differ across the *N* units. This is useful because given the interpretation of *λ_ij_* as potential unobserved confounders, one may expect them not to be similar across units, especially between control and treated units.

It is possible that each element *λ_ij_* of $\lambda_{i}$ affects only some of the outcomes. To address this issue, one potential solution would be to assume a different loadings vector for each outcome. However, this is inefficient, especially when *T_i_*’s are small (see simulation study of Section 5). Another solution would be to assume that there are loadings that are specific to each outcome and loadings that are shared by all outcomes (De Vito *et al.*, 2021). However, such an approach does not allow for loadings that are shared by subsets of the outcomes. Grabski *et al.* (2023) propose a model for normal outcomes that allows for shared loadings among a subset of outcomes. However, $v_{j\ell }$ are effectively either zero or one, thus not allowing loadings to affect the outcomes to different extents (which is the case when $0<v_{j\ell }<1$). Here, we allow for this via the prior on the factor parameters.

Let *f_tdj_*, *g_tdj_*, and *h_tdj_* denote the elements of $f_{td},\;g_{td}$, and $h_{td}$, respectively ($j=1,\ldots ,J^{*}$). We assume that $f_{\mathrm{tdj}}∼N(0,s_{1,dj}),\;g_{\mathrm{tdj}}∼N(0,s_{2,dj})$ and $h_{\mathrm{tdj}}∼N(0,s_{3,dj})$. For each *j*, let $v_{j}$ be a vector of size *D* with elements $v_{j\ell }$ defined as

$$v_{j\ell }=\{\begin{array}{cc} s_{1,\ell ,j}, & \ell \leq D_{1}\\ s_{2,\ell -D_{1},j}, & D_{1}<\ell \leq D_{1}+D_{2}\\ s_{3,\ell -D_{1}-D_{2},j}, & \ell >D_{1}+D_{2} \end{array}.$$

We introduce variables $M_{j}\in \left\{1,\ldots ,D\right\}$ which, for each *j*, indicate the outcome “most affected” by loading *j*. Conditional on *M_j_* = *l*, we set *v_jl_* = 1 and assume $v_{j\ell }∼\text{Uni}[0,1]$ for each ℓ≠l. When $v_{j\ell }$ is close to zero for some ℓ, the values of the corresponding factor will be close to zero, and hence the effect of loading *j* on outcome ℓ will be small. An alternative (which we test on our real data) is to let $v_{j\ell }∼\text{Uni}[0,1]$ for all *j* and ℓ. This introduces more free parameters than needed but has worked well in previous studies (Roy *et al.*, 2021).

For fixed *d* and *j*, we have chosen not to impose any temporal structure on ${\left\{f_{\mathrm{tdj}}\right\}}_{t=1}^{T}$ (or ${\left\{g_{\mathrm{tdj}}\right\}}_{t=1}^{T},\;{\left\{h_{\mathrm{tdj}}\right\}}_{t=1}^{T}$) to simplify posterior computations. We expect the loss in efficiency to be small. The reason is that in policy evaluation problems, the number of control units at each time point is typically sufficiently high to allow accurate estimation of factor parameters without the need to impose further structure, see e.g., simulation study in Samartsidis *et al.* (2020).

For the remaining model parameters, we specify weakly informative prior distributions. For all *i* and $d=1,\ldots ,D_{1}$, we assume $\sigma_{id}^{2}∼\text{Uni}(0,10^{2})$. We prefer this prior to the conjugate IG(ϵ,ϵ) with small *ϵ*, because the latter encourages values of $\sigma_{id}^{2}$ near zero. This can lead to over-fitting as non-systematic fluctuations are encouraged to be absorbed by the loadings/factors term. For all ℓ=1,2,3 and *d*, we assume that regression coefficients $\eta_{\ell ,d}∼N(0,10^{2}I)$. For $d=1,\ldots ,D_{3}$, we let $\xi_{d}∼\text{Uni}(0,20),$ i.e., we assume that the variance of the counts is at most 20 times higher compared to a Poisson model. For each loading *j*, we set $P(M_{j}=\ell )=1/D$ for ℓ=1,…,D.

Finally, we set $a_{p}=b_{p}=1$ i.e., let $p_{\mathrm{itd}}^{\left(T_{i}\right)}∼\text{Uni}(0,1)$. The choice of prior on $p_{\mathrm{itd}}^{\left(T_{i}\right)}$ is not crucial for the LTP data as *n_itd_* ($t>T_{i}$) are large, allowing us to estimate these parameters accurately. However, when *n_itd_* are low, the posterior uncertainty in $p_{\mathrm{itd}}^{\left(T_{i}\right)}$ under independent uniform priors will be high, leading to high uncertainty in *β_itd_* (see (3.3)). In such cases, efficiency can be gained by imposing structure on $p_{\mathrm{itd}}^{\left(T_{i}\right)}$. For example, one can assume that for fixed *i* and *d*, $\text{logit}(p_{\mathrm{itd}}^{\left(T_{i}\right)})$ arise from an AR(1) process.

## 4 Posterior computations

We are interested in drawing samples from the high-dimensional posterior with density

(4.6) $$\pi \left({\left\{\lambda_{i},{\left\{\xi_{id}\right\}}_{d=1}^{D_{3}}\right\}}_{i=1}^{N},{\left\{{\left\{g_{td}\right\}}_{t=1}^{T},\eta_{2,d}\right\}}_{d=1}^{D_{2}},{\left\{{\left\{h_{td}\right\}}_{t=1}^{T},\eta_{3,d}\right\}}_{d=1}^{D_{3}},\theta |\text{data}\right),$$

where

$$\theta =\left\{{\left\{{\left\{f_{td}\right\}}_{t=1}^{T},{\left\{\sigma_{id}^{2}\right\}}_{i=1}^{N}\right\}}_{d=1}^{D_{1}},{\left\{\xi_{d}\right\}}_{d=1}^{D_{3}},{\left\{{\left\{\phi_{ij}\right\}}_{i=1}^{N},\zeta_{j},{\left\{v_{j,l}\right\}}_{\ell =1}^{D},M_{j}\right\}}_{j=1}^{J^{*}},\rho \right\}$$

and

$$\text{data}={\left\{{\left\{{\left\{y_{\mathrm{itd}}\right\}}_{d=1}^{D_{1}},{\left\{k_{\mathrm{itd}},n_{\mathrm{itd}}\right\}}_{d=1}^{D_{2}},{\left\{z_{\mathrm{itd}},w_{\mathrm{itd}}\right\}}_{d=1}^{D_{3}},x_{it}\right\}}_{t=1}^{T_{i}}\right\}}_{i=1}^{N}.$$

The reason for grouping some parameters in θ is that they are easy to sample. Posterior (4.6) is analytically intractable; we thus use MCMC to sample from it. We propose a Metropolis-within-Gibbs sampler in which subsets of parameters are drawn (updated) in blocks from their full conditionals. Here, we provide an outline of the algorithm; for further details see Section B of the supplementary material available at *Biostatistics* online.

The parameters in θ are straightforward to update. We update each $\sigma_{id}^{2}$ (i=1,…,N and $d=1,\ldots ,D_{1}$) using a Metropolis–Hastings step. The $f_{td}$ (t=1,…,T and $d=1,\ldots ,D_{1}$) and $\eta_{1,d}$ ($d=1,\ldots ,D_{1}$) are drawn from their multivariate normal full conditional distributions. For the loadings shrinkage parameters, we follow Gao *et al.* (2016). They show that by using an equivalent representation of the prior (3.5), one can update all $\phi_{ij}$ (i=1,…,N and $j=1,\ldots ,J^{*}$), *ζ_j_* ($j=1,\ldots ,J^{*}$) and *ρ* using Gibbs steps. We draw each *M_j_* ($j=1,\ldots ,J^{*}$) from its full conditional (these parameters can only take *D* values), after integrating out *v_dj_* (d=1,…,D). Conditional on *M_j_*, the *v_dj_* (d=1,…,D and $j=1,\ldots ,J^{*}$) are either set to one or drawn from their truncated inverse-gamma full conditionals (see Section B of the supplementary material available at *Biostatistics* online).

The most challenging step in the proposed MCMC algorithm is to update $\lambda_{i},\;g_{td}$, and $h_{td}$. Their full conditionals are not available in closed form and hence Gibbs updates are not possible. Moreover, standard algorithms which employ a constant variance–covariance matrix in their proposal (e.g., MALA, preconditioned MALA, HMC) can be inefficient in FA models. This due to the label-switching, rotation, and scale ambiguity problems (Zhao *et al.*, 2016, among others). Consider, for example, the choice of a proposal variance for an element *h_tdj_* of $h_{td}$. Tuning this value is nontrivial as *h_tdj_* may represent a different factor (due to label-switching) or change its scale (due to scale ambiguity) at each iteration of MCMC. To overcome these problems, we make use of data augmentation techniques. In particular, we employ methods developed in the case of univariate models (Polson *et al.*, 2013; Zhou and Carin, 2015). As explained below, introducing auxiliary variables enables efficient sampling of $\lambda_{i},\;g_{td}$, and $h_{td}$. Finally, we note that under the proposed data augmentation scheme, drawing the remaining parameters $\eta_{2,d},\;\eta_{3,d}$, and *ξ_d_* is also conducted efficiently, see the remaining of this section.

Let PG(a,b) denote the Pólya–Gamma distribution with parameters *a* and *b*. Following Polson *et al.* (2013), for each $i=1,\ldots ,N,\;t\leq T_{i}$ and $d=1,\ldots ,D_{2}$, we introduce latent variables *ω_itd_* which are *a priori* $\text{PG}(n_{\mathrm{itd}},0)$ distributed. Using results from Polson *et al.* (2013), we can show that the likelihood contribution of each binomial data point conditional on *ω_itd_* is

(4.7) $$\pi (k_{\mathrm{itd}}|n_{\mathrm{itd}},\lambda_{i},g_{td},\eta_{2,d},x_{it},\omega_{\mathrm{itd}})\propto \;\text{exp}\;\left\{-\frac{\omega_{\mathrm{itd}}}{2}{\left(\frac{\kappa_{\mathrm{itd}}}{\omega_{\mathrm{itd}}}-\lambda_{i}^{⊤}g_{td}-\eta_{2,d}^{⊤}x_{it}\right)}^{2}\right\},$$

where $\kappa_{\mathrm{itd}}=k_{\mathrm{itd}}-n_{\mathrm{itd}}/2$. The likelihood (4.7) is a quadratic form of both $g_{td}$ and $\eta_{2,d}$, and these parameters have multivariate normal priors. Therefore, introducing *ω_itd_* allows us to draw each $g_{td}$ ($d=1,\ldots D_{2}$ and t=1,…,T) and $\eta_{2,d}$ ($d=1,\ldots ,D_{2}$) from their multivariate normal full conditionals (see SM Section B). Further, the full conditional of *ω_itd_* is a $\text{PG}(n_{\mathrm{itd}},\lambda_{i}^{⊤}g_{td}+\eta_{2,d}x_{it})$ (Polson *et al.*, 2013), a result which allows us to update these parameters easily.

Let CRT(a,b) denote the Chinese restaurant table distribution with parameters *a* and *b*. Following Zhou and Carin (2015), for each $i=1,\ldots ,N,\;t\leq T_{i}$ and $d=1,\ldots ,D_{3}$, we introduce latent variables $L_{\mathrm{itd}}∼\text{CRT}(z_{\mathrm{itd}},w_{\mathrm{itd}}q_{\mathrm{itd}}/\xi_{d})$. These can be drawn as $L_{\mathrm{itd}}=\sum_{l=1}^{z_{\mathrm{itd}}}b_{l}$, where $b_{l}∼\text{Bernoulli}(\frac{w_{\mathrm{itd}}q_{\mathrm{itd}}/\xi_{d}}{w_{\mathrm{itd}}q_{\mathrm{itd}}/\xi_{d}+l-1})$. Recall that $q_{\mathrm{itd}}=\text{exp}\;\left(\lambda_{i}^{⊤}h_{td}+\eta_{3,d}^{⊤}x_{it}\right)$. From Section 3.1 of Zhou and Carin (2015), we have that

(4.8) $$\begin{array}{c} \pi \left(z_{\mathrm{itd}},L_{\mathrm{itd}}|w_{\mathrm{itd}},q_{\mathrm{itd}},x_{it},\xi_{d}\right)=\text{CRT}\left(L_{\mathrm{itd}};z_{\mathrm{itd}},\frac{w_{\mathrm{itd}}q_{\mathrm{itd}}}{\xi_{d}}\right)\text{NegBin}\left(z_{\mathrm{itd}};\frac{w_{\mathrm{itd}}q_{\mathrm{itd}}}{\xi_{d}},\frac{1}{1+\xi_{d}}\right)\\ =\frac{\xi_{d}^{z_{\mathrm{itd}}}L_{\mathrm{itd}}!|S(z_{\mathrm{itd}},L_{\mathrm{itd}})|}{{\left(1+\xi_{d}\right)}^{z_{\mathrm{itd}}}{\left(\text{log}(1+\xi_{d})\right)}^{L_{\mathrm{itd}}}}\text{Pois}\left(L_{\mathrm{itd}};\frac{w_{\mathrm{itd}}q_{\mathrm{itd}}}{\xi_{d}}\text{log}(1+\xi_{d})\right), \end{array}$$

where *S*(*a*, *b*) are Stirling numbers of the first kind. In (4.8), *q_itd_* only appear in the second term (Poisson probability mass function). As shown in Section A of the supplementary material available at *Biostatistics* online, this enables us to update $h_{td}$ ($d=1,\ldots ,D_{3}$, and t=1,…,T), $\eta_{3,d}$ ($d=1,\ldots ,D_{3}$) and $\lambda_{i}$ (i=1,…,N) using the simplified manifold Metropolis adjusted Langevin (SMMALA) algorithm (Girolami and Calderhead, 2011), which would be very hard unless introducing the latent variables *L_itd_* and *ω_itd_*. The SMMALA algorithm exploits the geometry of the parameter space to adapt the variance–covariance matrix of its normal proposal at each iteration of MCMC, thus mitigating the problems caused by label-switching and scale ambiguity explained above. In our simulation studies, we found that SMMALA massively outperformed MALA and HMC, for which convergence was extremely slow.

Finally, we update each negative binomial dispersion parameter *ξ_d_* ($d=1,\ldots ,D_{3}$) using the Barker method (Livingstone and Zanella, 2022). To reduce dependence on the *L_itd_* ($t=1,\ldots ,T_{i}$), we integrate these parameters out when updating *ξ_d_*. This is straightforward using (4.8), see Section A of the supplementary material available at *Biostatistics* online. The Barker method requires the specification of a stepsize parameter; following Livingstone and Zanella (2022), we tune this for each *i* during the burn-in phase of the MCMC to achieve an acceptance rate near 40%.

## 5 Simulation studies

We perform a simulation study to demonstrate benefits, in terms of efficiency of causal effect estimates, that can be obtained by modeling multiple outcomes jointly (i.e., loadings are shared across outcomes) rather than individually (i.e., each outcome has its own loadings matrix). See Section C of the supplementary material available at *Biostatistics* online for additional simulations in settings that differ from the one considered here.

### 5.1 Data generating mechanism

We simulate *B* = 2, 500 synthetic data sets. For each one, we generate the untreated outcomes from the FA model of (3.1) with $D_{1}=D_{2}=D_{3}=1$, *T* = 24 (2 years of monthly data), *N* = 80 and $\lambda_{i},f_{t},g_{t},h_{t}\in R^{7}$. Since we consider only one outcome of each type, we omit the outcome index *d* for the remainder of this section to ease notation.

For all *i*, we set $\lambda_{i1}=1$ (to control the mean), and draw $\lambda_{ij}∼N(0,1)$ for j=2,…,7. Let ${\tilde{f}}_{j}={\left(f_{1j},\ldots ,f_{Tj}\right)}^{⊤},\;{\tilde{g}}_{j}={\left(g_{1j},\ldots ,g_{Tj}\right)}^{⊤}$, and ${\tilde{h}}_{j}={\left(h_{1j},\ldots ,h_{Tj}\right)}^{⊤}$. For each *j*, we generate ${\tilde{f}}_{j},\;{\tilde{g}}_{j}$ and ${\tilde{h}}_{j}$ from a $N(m_{1j}1,s_{1j}^{2}R),\;N(m_{2j}1,s_{2j}^{2}R)$ and $N(m_{3j}1,s_{3j}^{2}R)$, respectively, where 1 is a *T*-vector of ones and ***R*** is a *T* × *T* correlation matrix. The values of *m_dj_* and $s_{\ell j}$ (ℓ=1,2,3) are shown in Table 1. These specifications imply that $E(\mu_{it})=5,\;E(p_{it})=0.6$, and $E(q_{it})=4$. Some of the $s_{\ell j}$ are set to zero so that each loading *j* affects only a subset of the outcomes. For fixed outcome, the nonzero $s_{\ell j}$ are all equal for simplicity. The values of $s_{\ell }$ are chosen such that the 97.5% quantiles of *μ_it_*, *p_it_*, and *z_it_* (over units, times, and simulations) are roughly 7.5, 0.85, and 10, respectively. ***R*** has elements $R_{ts}=\text{exp}(\text{log}(0.8)|t-s|)$. This nondiagonal ***R*** introduces temporal correlations within the data on each unit.

**Table 1:** Mean and standard deviation used to simulate the seven factors of Section 5.

	Mean			Standard deviation		
*j*	Normal	Binomial	Count	Normal	Binomial	Count
1	$m_{11}=5$	$m_{21}=\text{logit}(0.6)$	$m_{31}=\text{log}(4)$	0	0	0
2	0	0	0	*s*_1_	*s*_2_	0
3	0	0	0	*s*_1_	0	*s*_3_
4	0	0	0	0	*s*_2_	*s*_3_
5	0	0	0	*s*_1_	0	0
6	0	0	0	0	*s*_2_	0
7	0	0	0	0	0	*s*_3_

The value of *σ* is chosen such that $\lambda_{i}^{⊤}f_{t}$ accounts for 80% of the variability in the *y_it_*. We draw the *n_it_* from a $\text{Pois}({\tilde{n}}_{it})$ distribution. For each *i*, ${\tilde{n}}_{i1}=5,\;{\tilde{n}}_{iT}$ is drawn from a Uni(50,200) distribution and ${\tilde{n}}_{it}={\tilde{n}}_{i1}+\frac{t-1}{T-1}({\tilde{n}}_{iT}-{\tilde{n}}_{i1})$ for 1<t<T. Similarly, we draw the *w_it_* from a $\text{Pois}({\tilde{w}}_{it})$ distribution where for each *i*, ${\tilde{w}}_{i1}=5,\;{\tilde{w}}_{iT}$ is drawn from a Uni(25,75) distribution and ${\tilde{w}}_{it}={\tilde{w}}_{i1}+\frac{t-1}{T-1}({\tilde{w}}_{iT}-{\tilde{w}}_{i1})$ for 1<t<T. We have made ${\tilde{n}}_{it}$ and ${\tilde{w}}_{it}$ increasing to mimic the LTP data of Section 5. Finally, we set *ξ* = 2.

In each data set, *T_i_* are chosen as follows. For every *i* and *t*, we draw $u_{it}∼\text{Uni}(0,1)$ and let

(5.9) $$\varpi_{it}=\{\begin{array}{cc} \text{expit}\left(\kappa_{0}+\kappa_{1}p_{it}+\kappa_{2}q_{it}\right) & t>t_{\text{min}}\\ 0 & t\leq t_{\text{min}} \end{array},$$

where expit(·)=exp(·)/(1+exp(·)). Then, for each *i* we set $T_{i}=\text{min}\left\{t:u_{it}<\varpi_{it}\right\}$. We set the minimum number of pre-intervention time points to $t_{\text{min}}=8$. The value of *κ*_0_ is chosen such that the average over simulated data sets *N*1 is 40. The values of *κ*_1_ and *κ*_2_ control the degree of unobserved confounding of the effects *β_it_* and *δ_it_*, respectively. We set *κ*_1_ such that the average (over simulated data sets) value of the empirical completion probability $k_{it}/n_{it}$ is roughly 10% higher in controls units for all $t>t_{\text{min}}$. The value of *κ*_2_ is chosen such that on average (over simulated data sets), $z_{it}/w_{it}$ is roughly 10% higher in controls units for every $t>t_{\text{min}}$.

For each simulated data set *b*, we perform a multivariate (MV) and a univariate (UV) analysis. MV analysis is carried out by fitting the FA model of Section 3 to all three outcomes jointly. UV analysis is carried out by fitting the FA model of Section 3 to each one of the outcomes individually. In both cases, the models that we fit are correctly specified. For both MV and UV analyses, we run MCMC for 100,000 iterations, saving posterior draws every 50 iterations to obtain 2,000 posterior draws. Of these, 500 are discarded as burn-in. The maximum number of factors $J^{*}$ in MV and UV analyses is set to 25 and 15, respectively.

For each *b*, we generate the data of treated units in their post-intervention period under *L* = 50 different scenarios, corresponding to causal effects of increasing magnitude. Thus, we obtain *L* different estimates (and CIs) for all causal effects defined in Section 3. For each ℓ=1,…,L, let ${\tilde{\alpha }}_{it}^{\left(\ell \right)},\;{\tilde{\beta }}_{it}^{\left(\ell \right)}$, and ${\tilde{\delta }}_{it}^{\left(\ell \right)}$ be the causal effect of intervention on *μ_it_*, $\text{logit}(p_{it})$ and $\text{log}\;\left(q_{it}\right)$, respectively. We use the tilde ($\tilde{\cdot }$) notation to indicate that these are the effects on the mean function rather than on the outcome as in (3.2). For the binomial and count outcomes, the effects are applied on the logit-scale and log-scale, respectively, to avoid negative values. For each ℓ, we assume that for all $i:T_{i}<T$ and $t>T_{i},\;{\tilde{\alpha }}_{it}^{\left(\ell \right)}={\tilde{\alpha }}^{\left(\ell \right)},\;{\tilde{\beta }}_{it}^{\left(\ell \right)}={\tilde{\beta }}^{\left(\ell \right)}$, and ${\tilde{\delta }}_{it}^{\left(\ell \right)}={\tilde{\delta }}^{\left(\ell \right)}$. The magnitude of ${\tilde{\alpha }}^{\left(\ell \right)},\;{\tilde{\beta }}^{\left(\ell \right)}$, and ${\tilde{\delta }}^{\left(\ell \right)}$ increases with ℓ and ${\tilde{\alpha }}^{\left(1\right)}={\tilde{\beta }}^{\left(1\right)}={\tilde{\delta }}^{\left(1\right)}=0$. Despite considering *L* scenarios for the intervention effect, we only need to run the MV and UV analyses once for each *b*, as the draws from the posterior of the potential untreated outcomes do not depend on postintervention data.

### 5.2 Results

We report results only for $C_{i}=\left\{\alpha_{i},\beta_{i},\gamma_{i},\delta_{i}\right\}$, representing the average response of each unit to the intervention. We compare the performance of the MV and UV approaches in terms of (i) the bias of the point estimates of the $C_{i}$; (ii) the standard error of the point estimates of the $C_{i}$; (iii) the width of the 95% CIs of $C_{i}$; (iv) the probability of detecting an intervention effect (power). For scenario ℓ=1 (i.e., no intervention effect), we define a detection as a CI that does not include zero to obtain the false positive rate. For any other scenario (i.e., positive intervention effect), we define a detection as a CI whose lower bound is larger than zero. For each estimand in $C_{i}$, we summarize each performance measure by taking the weighted average over simulated data sets and treated units. The weights are introduced to account for the varying number of treated units. More specifically, we assign a weight of $1/(BN_{2}^{\left(b\right)})$ to each treated unit in simulated data set *b*, where $N_{2}^{\left(b\right)}$ is the total number of treated units in that data set. To investigate the impact of *T_i_*, we further calculate the measures over simulations and units with *T_i_* = 8, *T_i_* = 16, and *T_i_* = 23.

The results for scenario ℓ=1 (no intervention effect) are presented in Table B.1 of the supplementary material available at *Biostatistics* online. Overall, there are no major problems with the estimates provided by UV and MV approaches: the bias of the point estimates of $C_{i}$ is negligible (compared to the standard deviation), and the false positive rates are close to the nominal 5%. The exceptions are the estimates of *β_i_*, *γ_i_*, and *δ_i_* provided by UV approach for *T_i_* = 8 i.e., for units with limited data in the preintervention period. For these, we find considerable bias (compared to the standard error) and inflated false positive rates. The estimates of *α_i_* are not affected because there is no confounding of these effects: the $\varpi_{it}$ in (5.9) are not a function of *μ_it_*.

In terms of efficiency, we see that the MV approach outperforms the UV approach. In particular, the standard errors of the point estimates of $C_{i}$, as well as the width of CIs, are on average smaller for the MV approach, as can be seen in Table 1 of Appendix B of the supplementary material available at *Biostatistics* online. The gains in efficiency due to joint outcome modeling are higher when *T_i_* is small. This is expected, because when *T_i_* is large there is sufficient data per treated unit on each outcome to estimate the loadings and so there is less benefit from using data on the other outcomes. Finally, it is worth noting that for all estimands and both approaches, the measures of efficiency (standard error, CI width) are better for *T_i_* = 16 than they are for *T_i_* = 8 and *T_i_* = 23. The reason is that the efficiency in the estimates of $C_{i}$ improves with both *T_i_* and $T-T_{i}$ (total number of postintervention time points). When *T_i_* increases, there is more data to estimate the loadings and thus the potential untreated outcomes. When $T-T_{i}$ increases, there is more data for each one of the $C_{i}$. In our simulation with fixed *T*, units of moderate *T_i_* achieve the better balance between *T_i_* and $T-T_{i}$.

**Fig. 2:**
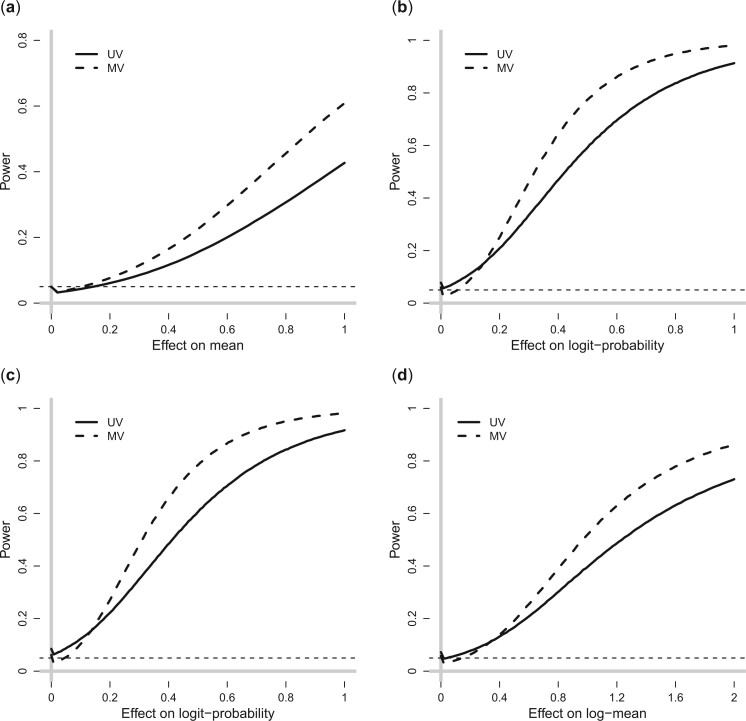
Power of detecting an intervention effect for a randomly chosen treated unit (*y* axis) as a function of the magnitude of the intervention effect (*x* axis). (a)–(d) correspond to causal effects *α_i_*, *β_i_*, *γ_i_*, and *δ_i_*, respectively. The results are based on 2,500 simulated data sets.

The bias of point estimates, standard error of point estimates and width of CIs in scenarios ℓ>1 are very similar to scenario ℓ=1 and therefore not discussed^3^. Figure 2 shows the power achieved by the UV and MV approaches across all the different scenarios. We see that for all four estimands, the gains in efficiency due to joint outcome modeling substantially improve the probability of detecting a nonzero intervention effect. For example, ${\tilde{\beta }}_{i}=0.4$ is detected with probability 44% using the UV approach, whereas it is detected with probability 65% when using the MV approach. Figure 2 in Appendix B of the supplementary material available at *Biostatistics* online shows the power for different values of *T_i_*. For reasons explained above, we see that the improvements in power achieved by the MV approach compared to the UV are greater when *T_i_* is either 8 or 16.

**Fig. 3:**
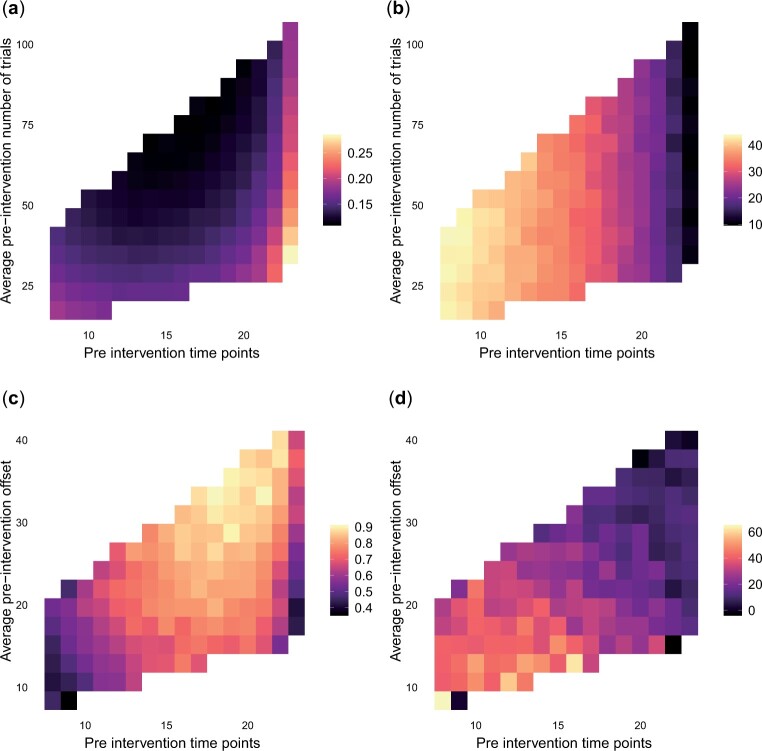
Effect of *n_it_* and *w_it_* on the efficiency of the causal estimates. (a) average (over simulated data sets and treated units) CI width of *β_i_* achieved by the MV model, as a function of ${\tilde{n}}_{i}=\sum_{t=1}^{T_{i}}n_{it}/T_{i}$ and *T_i_*. (b) average (over data sets and units) % reduction in CI width of *β_i_* as a function of ${\tilde{n}}_{i}$ and *T_i_* achieved by the MV model compared to the UV. (c) power of detecting a moderate intervention effect from *δ_i_* achieve by the MV model, for different values of ${\tilde{w}}_{i}=\sum_{t=1}^{T_{i}}w_{it}/T_{i}$. (d) % increase in power of detecting a moderate effect on *δ_i_* achieved by the MV model compared to the UV. In all panels, entries that were obtained as the average of less than 50 simulated data sets were discarded.

For binomial outcomes, the uncertainty in the estimates of a treated unit’s potential untreated outcomes *p_it_* and $k_{it}^{\left(0\right)}$, and thus the accuracy of the estimates of *β_it_* and *γ_it_*, depends on both *T_i_* and the values of *n_it_* in the pre-intervention period. More specifically, the lower the counts ${\left\{n_{it}\right\}}_{t=1}^{T_{i}}$ are, the less information to estimate the loadings there is. Similarly, for count outcomes, the uncertainty in the estimates of a treated unit’s $z_{it}^{\left(0\right)}$ ($t>T_{i}$) depends on ${\left\{w_{it}\right\}}_{t=1}^{T_{i}}$. Figure 3(a) presents a heatmap of the CI width for $\beta_{i}$ obtained by the MV approach for different combinations of *T_i_* and ${\bar{n}}_{i}=\frac{1}{T_{i}}\sum_{i=1}^{T_{i}}n_{it}$. For any fixed *T_i_*, the width of CIs decreases with ${\bar{n}}_{i}$. Figure 3(b) shows the percentage decrease in CI width achieved by the MV approach compared to the UV approach for different combinations of *T_i_* and ${\bar{n}}_{i}$. We see that for fixed *T_i_*, the gains in efficiency due to joint outcome modeling are similar for the different values of ${\bar{n}}_{i}$. For the count outcome, we present the power achieved for moderate ${\tilde{\delta }}_{i}$ (≈1.25) as a measure of efficiency; see Fig. 3(c). We find that power increases with ${\bar{w}}_{i}$ for fixed *T_i_*. Figure 3(d) shows the percentage increase in power for moderate $\tilde{\delta }$ achieved by the MV approach compared to the UV. Again, we see that for fixed *T_i_*, the gains do not differ much over across the ${\bar{w}}_{i}$.

## 6 Evaluation of LTPs

**Fig. 4:**
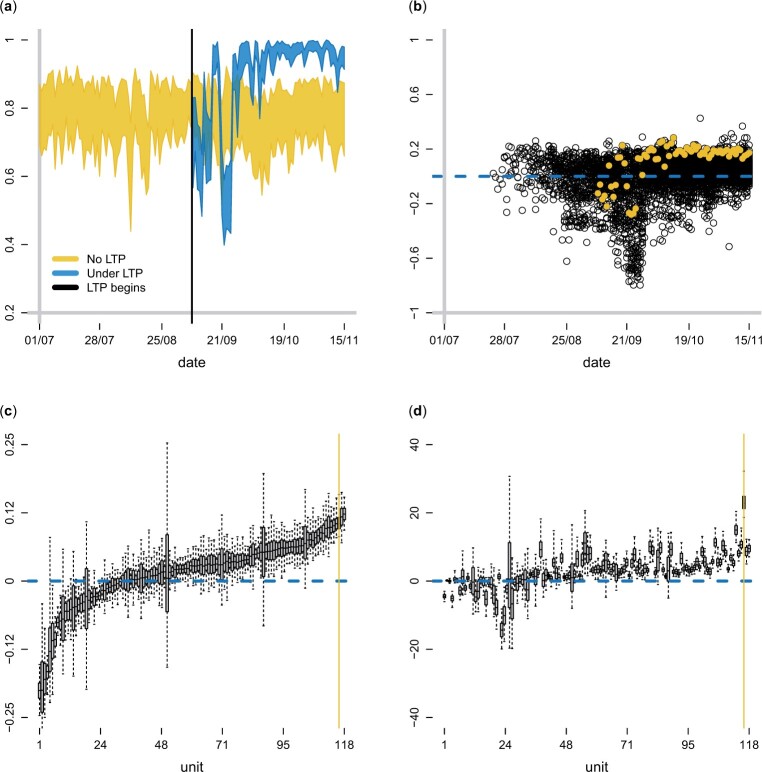
Results for outcome *completion*. (a) 95% CIs for $p_{\iota t1}$ and $p_{\iota t1}^{\left(T_{\iota }\right)}$, where *ι* is a unit chosen for illustration. (b) scatterplot of the posterior mean of $\beta_{it1}$. Filled yellow dots represent $\beta_{\iota t1}$. Panels (c) and (d) show posterior summaries of $\beta_{i1}$ and $\gamma_{i1}$, respectively, where units have been ordered by increasing mean posterior $\beta_{i1}$ in both plots. The vertical yellow lines indicate unit *ι*. Boxes and whiskers represent the 75% and 95% CIs, respectively.

In this section, we apply our multivariate FA model to the LTP data of Section 2. We set $J^{*}=25$ and ran three MCMC chains starting from different initial values. For each chain, we ran for 250,000 iterations, discarding the first 50,000 as burn-in, and thinning the remaining draws at every 100 iterations to obtain 2,000 draws from the posterior. Convergence was assessed visually by comparing the posterior densities and trace plots obtained from the three chains for multiple randomly selected causal estimands; these showed that all three chains have converged to the same stationary distribution and that mixing was good. Results for the case completion outcome are shown in Fig. 4. Figures D.2, D.3, and D.4 of the supplementary material available at *Biostatistics* online show results for the remaining outcomes.

**Potential outcomes**. Figure 4(a) refers to a unit, say *ι*, chosen for illustration. The plot shows 95% CIs for $p_{\iota t1}$ and $p_{\iota t1}^{\left(T_{\iota }\right)}$ in yellow and blue, respectively. For most days, the bands for $p_{\iota t1}^{\left(T_{\iota }\right)}$ are above the bands for $p_{\iota t1}$, indicating that the LTP had a positive effect on completion probability on these days. We also see that the 95% CIs for $p_{\iota t1}$ are substantially wider than the CIs for $p_{\iota t1}^{\left(T_{\iota }\right)}$; this is due to the large number of cases in the post-LTP period, allowing $p_{\iota t1}^{\left(T_{\iota }\right)}$ to be estimated with high precision. Hence, uncertainty in $\beta_{\iota t1}$ is mainly due to the uncertainty in $p_{\iota t1}$.

**Effects by unit and day**. Scatterplots of the posterior means of $\beta_{it1}$ and $\gamma_{it1}$ are shown in Fig. 4(b) and Figure D.5(a), respectively, where the $\beta_{\iota t1}$ and $\gamma_{\iota t1}$ are shown in yellow. There is considerable heterogeneity in the estimated effects, both in terms of magnitude and sign. We observe an adverse effect during mid-September, a period during which several of the UTLAs operating LTPs reported that the number of people employed locally for contact tracing was insufficient to deal with the increasing number of cases. However, there are overall more positive than negative effects (especially toward the end of the study), suggesting that LTPs improved case completion TT on average.

There is also considerable heterogeneity of estimated effects for the other outcomes, see Section D of the supplementary material available at *Biostatistics* online. For timely case completion, there are both periods with positive and periods with negative effects (Fig. D.2(a) and (b) of the supplementary material available at *Biostatistics* online). We note that when we considered timely case completion out of $n_{it1}$ (rather than $k_{it1}$), the results (not shown) suggested that timeliness improves. This is expected, because LTPs lead to additional cases being completed, some of which are completed within 48 hours. For contact completion (Fig. D.3 of the supplementary material available at *Biostatistics* online) and number of contacts (Fig. D.4 of the supplementary material available at Biostatistics online), the estimated effects are centered around zero indicating that on average, LTPs did not affect the performance of TT for these outcomes.

A possible explanation as to why LTPs appear to improve TT performance in some units/days while negatively affecting it in some others is that the model of LTP employed varies between units (e.g., 5-day vs. 7-day working pattern or larger teams vs. smaller teams). Population factors, such as demographic characteristics of the cases, may also play a role. Finally, it is possible that staff delivering LTPs were becoming more effective as they acquired more experience.

**Average unit effects**. The posterior distribution of the average (over time) unit effects $\beta_{i1}$ is summarized in Fig. 4(c), where we have sorted units by increasing mean posterior $\beta_{i1}$. There are units that benefited from the LTPs on average ($\text{Pr}\left(\beta_{i1}<0\right)$ small), units that showed an adverse effect on average ($\text{Pr}\left(\beta_{i1}>0\right)$ small), and units that seemed unaffected (95% CIs centered at zero). This is also the case for the other three outcomes (see Section D of the supplementary material available at *Biostatistics* online). Figure 4(d) summarizes the posterior distribution of $\gamma_{i1}$, where units are sorted as in Fig. 4(c). Note that LTPs can have a strong effect (positive or negative) on the $\gamma_{i1}$ (or $\gamma_{i2},\;\gamma_{i3}$) of a unit even if $\beta_{i1}$ (or $\beta_{i2},\;\beta_{i3}$) on the same unit is close to zero. This is because the latter measure depends on the total number of cases on each day: a 2% improvement in completion probability, will result in tens of additional completed cases in units with thousands of new cases, but possibly none in units with tens of new cases.

**Overall effects.** The estimated average effects over all units and post-intervention time points, *β_d_*, *γ_d_* (*d* = 1, 2, 3), and *δ*_1_, are presented in Table 2 of the supplementary material available at *Biostatistics* online. These results suggest that case completion benefited from the LTPs as we estimate an additional 3.61 (95% CI [3.02–4.26]) completed cases per unit per day). Further, it appears that LTPs led to a drop in the number of contacts (we estimate that on average, 1.99 less contacts per unit per day were obtained due to LTPs).

**Table 2:** Posterior mean along with 95% CIs for the “overall” effects *β_d_* (*d* = 1, 2, 3), *γ_d_* (*d* = 1, 2, 3) and *δ*_1_. We present overall effects for the entire study period, as well as the period October 1, 2020–November 15, 2020.

		Whole period		10/2020 onward	
Outcome	Effect	Estimate	95% CI	Estimate	95% CI
Completion	*β*_1_	0.013	0.006–0.021	0.044	0.038–0.051
	*γ*_1_	3.612	3.015–4.262	5.734	4.885–6.618
Timeliness	*β*_2_	–0.074	–0.080––0.068	0.014	0.007–0.020
	*γ*_2_	0.218	–0.221–0.660	1.973	1.367–2.602
Contact completion	*β*_3_	-0.014	–0.022––0.007	-0.007	–0.015–0.001
	*γ*_3_	-1.992	–3.743––0.439	-2.416	–4.746––0.411
Number of contacts	*δ*_1_	-0.552	–7.852–4.893	-0.994	–10.870–6.480

**Posterior ranks**. Identifying the units that benefit the least from their LTPs is important to improve the way of implementing LTPs in these units (e.g., by increasing the size of the teams). We do this using the posterior ranks. In Fig. D.6 of the supplementary material available at *Biostatistics* online, we summaries the posterior distribution of $r_{\iota t1}^{\left(\beta \right)},\;r_{\iota t2}^{\left(\beta \right)},\;r_{\iota t3}^{\left(\beta \right)}$, and $r_{\iota t1}^{\left(\delta \right)}$. For most of the days in unit *ι*’s postintervention period, the mean posterior $r_{\iota t1}^{\left(\beta \right)}$ is high (≈0.85), suggesting that unit *ι* was one of the units who benefited most from the LTPs in terms of case completion. The ranks for the remaining estimands as not as high.

**Sensitivity analyses**. In Section D of the supplementary material available at *Biostatistics* online, we perform a series of sensitivity to study the robustness of our findings to some hyper-parameter values and modeling choices. In particular, we repeat the analysis setting *ν* = 1, setting $J^{*}=15$ and $J^{*}=35$, as well as allowing the $v_{jl}∼\text{Uni}[0,1]$ for all *j* and ℓ. The results suggest that our findings are not sensitive to these specifications.

## 7 Discussion

Motivated by an application concerning COVID-19, we have proposed a novel methodology for evaluating the impact of an intervention using observational time-series data. This methodology generalizes an existing causal multivariate FA method for normally distributed outcomes (Samartsidis *et al.*, 2020) to the mixed outcome setting. This is an important addition to the literature in the field because, to our knowledge, it is the only method that can simultaneously (i) deal with outcomes of mixed type, (ii) make use of shared variability between subsets of multiple outcomes to improve the statistical efficiency of causal estimates, and (iii) provide uncertainty quantification for all causal estimands of interest.

We used the proposed methodology to estimate the impact of LTPs on the effectiveness of England’s NHS TT. The results suggest that on average, LTPs did not have a strong effect on contact completion or number of contacts elicited but improved case completion and timely case completion. However, there is big heterogeneity in the estimates of the causal effects both between and within (over time) units. It is likely that the heterogeneity is partly driven by differences in the model of LTP employed in each unit and day. Unfortunately, further investigation is not possible because this information has not been routinely collected. Nonetheless, our analyses highlight the importance of recording such data and we hope that they will encourage future collection.

Our approach leans itself to a number of extensions. First, it is likely that the potential untreated outcomes of units in spatial proximity are correlated, but our FA model does not make use of the geographical location of units. This could be addressed by assuming that for any *j*, $C\text{or}(\lambda_{ij},\lambda_{\iota j})$ is a function of the distance between units *i* and *ι*. Second, we have assumed that continuous outcomes are normally distributed. This assumption might not hold in some data sets, even after transformations are applied. Hence, it is worth considering more flexible continuous distributions, e.g., the Student *t* and Gamma. Third, we will impose temporal structure on the factor parameters (e.g., with splines) parameters, to improve efficiency further.

The computation time required to apply our method to a typically sized policy evaluation data set is reasonable. For example, MCMC took approximately 90 min and 8 h to run on the simulated and real data, respectively, both on a 3.6 GHz Intel i7 machine with 16 GB of RAM. The main computational complexity arises from the need to invert a $J^{*}\times J^{*}$ matrix to update each of the *N* vectors of loadings and *TD* vectors of factors and is thus $O\left((N+TD)J^{{*}^{3}}\right)$. This can be prohibitive when applying to data from other fields such as biology. To overcome this issue, we will consider alternative approaches for estimation, e.g., using variational methods.

## 8 Software

Implementation codes are available at https://github.com/psamartsidis/cbfa.git.

## Supplementary Material

kxad030_Supplementary_Data

## Footnotes

1 Cases are informed of their test result electronically (using the individual’s preferred means of communication), and may not necessarily be aware of their result at the time that TT attempt to get in touch with them.

2 In practice, one has to choose an arbitrary threshold *c* for $\left|\lambda_{ij}\right|$ to determine which columns are nonzero. This is not a problem in our application as we are not interested in making inference on *J* but rather account for uncertainty in this quantity when estimating the causal effects of interest.

3 For some of the estimands, this can be expected. Consider, for example, *α_i_*. Suppose that $T_{i}=T-1$ and let $\alpha_{i,\ell }$ and $y_{iT,\ell }$ be the simulated values of *α_i_* and *y_iT_* in scenario ℓ, respectively. For all ℓ, we will have that ${\hat{\alpha }}_{i,\ell }=y_{iT,\ell }-{\hat{y}}_{iT}^{\left(0\right)}=N(\mu_{iT}+\alpha_{i,\ell },\sigma_{i})-{\hat{y}}_{iT}^{\left(0\right)}\approx \left(N(\mu_{iT},\sigma_{i})+\alpha_{i,\ell }\right)-{\hat{y}}_{iT}^{\left(0\right)}\approx \alpha_{i,\ell }+(y_{iT,1}-{\hat{y}}_{iT}^{\left(0\right)})$. The argument extends to t<T−1. Hence, the bias in *α_i_* will only depend on the $y_{it,\ell }-{\hat{y}}_{it}^{\left(0\right)}$.
