# Supplementary material for: A Bayesian multivariate factor analysis model for causal inference using time-series observational data on mixed outcomes
Source: Biostatistics. 2023 Dec 6;25(3):867–84. doi: 10.1093/biostatistics/kxad030 (PMC11247182; doi:10.1093/biostatistics/kxad030)
Supplement: kxad030_Supplementary_Data [file kxad030_supplementary_data.zip › biosts-22315-File001.pdf]

Supplementary material for *'A Bayesian multivariate factor analysis model for causal inference using time-series observational data on mixed outcomes'*

P Samartsidis, SR Seaman, A Harrison, A Alexopoulos, GJ Hughes, C Rawlinson, A Charlett, I Oliver and D De Angelis

A Interpretation of causal effects

Recall that in the main text, we define causal effects $\gamma_{itd} = k_{itd} - k_{itd}^{(0)}$ and $\delta_{itd} = z_{itd} - z_{itd}^{(0)}$, where $k_{itd}^{(0)} \sim \text{Bin}\left(n_{itd}, p_{itd}\right)$ and $z_{itd}^{(0)} \sim \text{NegBin}\left(w_{itd}q_{itd}\xi_d^{-1}, (1+\xi_d)^{-1}\right)$. In this section, we provide an interpretation of these effects in the context of the LTP application. To simplify the exposition, we omit $\boldsymbol{x}_{it}$, k_{it2} and k_{it3}, and consider a fixed day (and so omit the time index). We further omit the unit index i to simplify the notation. So, here we consider only the number of cases reported on that day (denoted as N, not to be confused with the sample size), the number of these N cases that were eventually completed (denoted as K), the number of cases completed on that day (denoted as W [1]), and the number of contacts elicited from these W completed cases (denoted as Z).

We use the binary variable A to indicate whether the LTP was in force on that day for the unit: $A = 1$ if the unit was subject to a LTP; $A = 0$ otherwise. Suppose that one can separate [Robins and Richardson, 2011; Stensrud et al., 2022, among others] the LTP intervention into components A_1, A_2 and A_3, where A_1 an intervention designed to reduce the number of cases (e.g. by reducing the spread of the virus), A_2 is an intervention to improve the completion of cases, and A_3 is an intervention to improve the elicitation of contacts. This separation of the intervention into components is reflected by the directed acyclic graph (DAG) of Figure A.1, where Λ represents the vector of loadings defined in Section 3.1. An assumption that is implicit in this DAG is that there are no common causes (other than A_1, A_2 and A_3) of N, K, W and/or Z that are caused by A. That is, we do not allow any variables denoted V in the DAG of Figure A.2 to be present.

In the observed data, the values of the unit's three variables A_1, A_2, A_3 are all equal to its value of A: $A_1 = A_2 = A_3 = A$. That is, either all or none of the three components of the intervention are applied. However, we can consider the causal effect of applying some but not all of the components, and this is what we shall do now.

Let $\pi^{(a_1,a_2,a_3)}\left(\lambda, n, k, w, z\right)$ denote the joint distribution of $\{\Lambda, N, K, W, Z\}$ under the intervention that sets $A_1 = a_1$, $A_2 = a_2$ and $A_3 = a_3$. By the g-functional

[1] These W cases include a subset of the K cases just mentioned, specifically those that were completed on the same day that they were reported.

[Robins and Richardson, 2011], we have that

$$\begin{aligned}\pi^{(a_1,a_2,a_3)}(\lambda,n,k,w,z) &= \Pr(\Lambda=\lambda)\times\Pr(N=n\mid\Lambda=\lambda,A_1=a_1)\\ &\times\Pr(K=k\mid N=n,\Lambda=\lambda,A_2=a_2)\\ &\times\Pr(W=w\mid K=k,\Lambda=\lambda,A_2=a_2)\\ &\times\Pr(Z=z\mid W=w,\Lambda=\lambda,A_3=a_3).\end{aligned}$$

Hence, the conditional distribution of $\{N,K,W,Z\}$ given Λ under the intervention is

$$\begin{aligned}\pi^{(a_1,a_2,a_3)}(n,k,w,z\mid\lambda) &= \Pr(N=n\mid\Lambda=\lambda,A_1=a_1)\\ &\times\Pr(K=k\mid N=n,\Lambda=\lambda,A_2=a_2)\\ &\times\Pr(W=w\mid K=k,\Lambda=\lambda,A_2=a_2)\\ &\times\Pr(Z=z\mid W=w,\Lambda=\lambda,A_3=a_3). \qquad (1)\end{aligned}$$

First, consider the intervention that sets $A_1=1$ and $A_2=A_3=0$. We have that the conditional distribution of K given N and Λ under this intervention is

$$\begin{aligned}&\pi^{(1,0,0)}(k\mid n,\lambda)\\ &=\frac{\pi^{(1,0,0)}(n,k\mid\lambda)}{\pi^{(1,0,0)}(n\mid\lambda)}\\ &=\frac{\sum_w\sum_z\pi^{(1,0,1)}(n,k,w,z\mid\lambda)}{\sum_{k=1}^n\sum_w\sum_z\pi^{(1,0,1)}(n,k,w,z\mid\lambda)}\\ &=\frac{\Pr(N=n\mid\Lambda=\lambda,A_1=1)\Pr(K=k\mid N=n,\Lambda=\lambda,A_2=0)}{\Pr(N=n\mid\Lambda=\lambda,A_1=1)} \qquad (2)\\ &=\Pr(K=k\mid N=n,\Lambda=\lambda,A_2=0)\\ &=\Pr(K=k\mid N=n,\Lambda=\lambda,A_1=0,A_2=0,A_3=0) \qquad (3)\\ &=\Pr(K=k\mid N=n,\Lambda=\lambda,A=0), \qquad (4)\end{aligned}$$

where (2) is obtained by summing the right-hand side of (1) over w and z, and (3) holds because of the structure of the DAG in Figure A.1.

Second, consider the intervention that sets $A_1=A_2=1$ and $A_3=0$. Using the same approach that we used to obtain equation (4), we can show that the conditional distribution of K given N and Λ under this intervention is

$$\begin{aligned}&\pi^{(1,1,0)}(k\mid n,\lambda)\\ &=\Pr(K=k\mid N=n,\Lambda=\lambda,A_2=1)\\ &=\Pr(K=k\mid N=n,\Lambda=\lambda,A_1=1,A_2=1,A_3=1)\\ &=\Pr(K=k\mid N=n,\Lambda=\lambda,A=1). \qquad (5)\end{aligned}$$

Third, consider the intervention that sets $A_1=A_2=A_3=1$. Again using the same approach, we can show that the conditional distribution of K given N and

Λ under this intervention is

$$\begin{aligned}
&\pi^{(1,1,1)}(k \mid n, \lambda) \\
&= \Pr(K = k \mid N = n, \Lambda = \lambda, A_2 = 1) \\
&= \Pr(K = k \mid N = n, \Lambda = \lambda, A_1 = 1, A_2 = 1, A_3 = 1) \\
&= \Pr(K = k \mid N = n, \Lambda = \lambda, A = 1). \qquad (6)
\end{aligned}$$

Comparing equations (5) and (6), we see that

$$\pi^{(1,1,0)}(k \mid n, \lambda) = \pi^{(1,1,1)}(k \mid n, \lambda). \qquad (7)$$

In the paper, we assumed that the conditional distribution of K given $N = n, \Lambda = \lambda$ and $A = 0$ is the Binomial(n, p) distribution, where p is defined in Equation (3.1). Thus, it follows from Equation (4) that $\pi^{(1,0,0)}(k \mid n, \lambda)$ is the Binomial(n, p) distribution.

We defined the causal effect γ as $\gamma = k - k^{(0)}$ (we have dropped indices for simplicity). The former (i.e. k) is a realisation from $\pi^{(1,1,1)}(k \mid n, \lambda)$, or (because of equation (7)) $\pi^{(1,1,0}(k \mid n, \lambda)$. The latter (i.e. $k^{(0)}$) is drawn from a Binomial(n, p) distribution, which, as we have just shown, corresponds to $\pi^{(1,0,0)}(k \mid n, \lambda)$. This leads to the interpretation of γ as the separable direct effect of setting $A_2 = 1$ when A_1 has already been set to one. In other words, it is the causal effect of implementing the intervention to improve the completion of cases when the intervention to reduce the number of new cases has already been applied.

Similarly, δ can be shown to be the causal effect of implementing the intervention to improve the elicitation of contacts when the intervention to reduce the number of new cases and the intervention to improve the completion of contacts have already been applied.

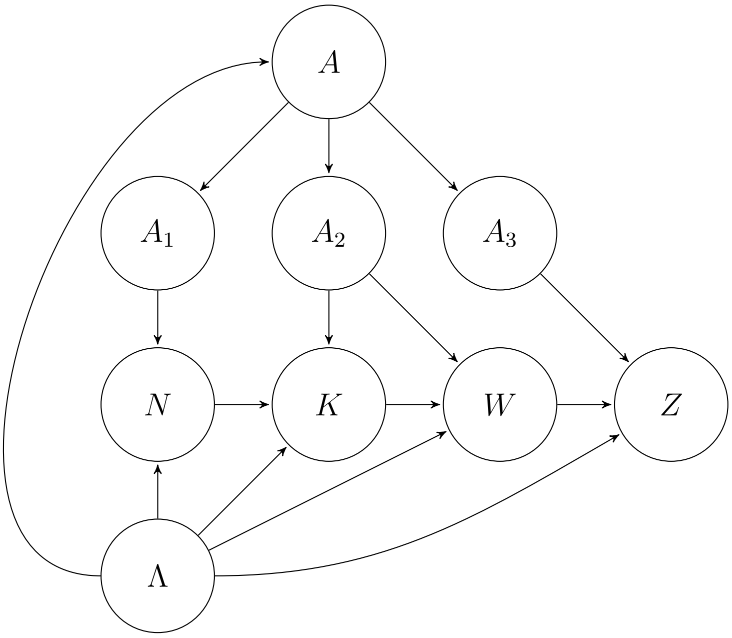

Figure A.1: Causal DAG with intervention A separated into three components

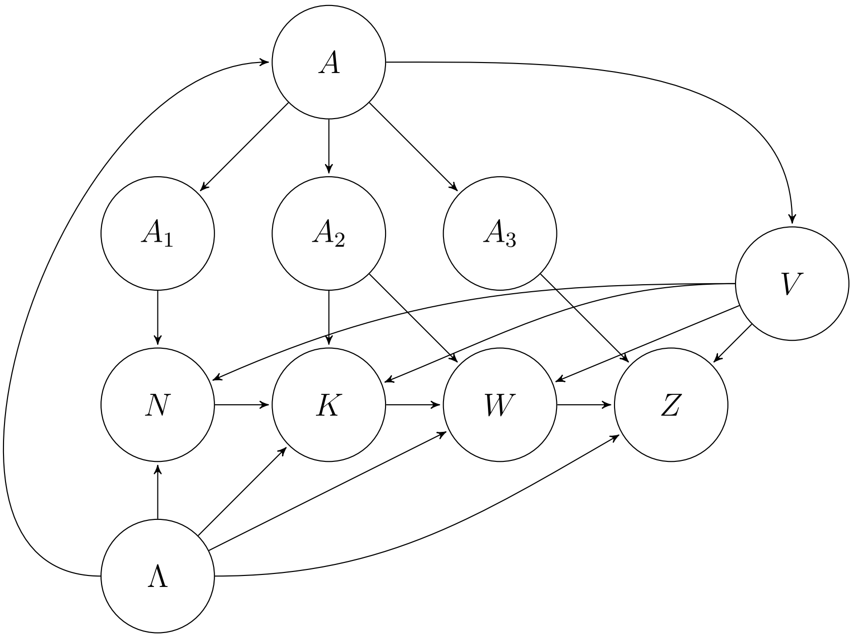

Figure A.2: The same causal DAG but with additional variable V that is not allowed to be present

B Markov chain Monte Carlo algorithm

In this section we present the details of the Metropolis-within-Gibbs sampler outlined in Section 4. A sketch of the sampler is shown in Algorithm 1. Details for some of the steps are presented in the following subsections. Throughout, we shall

use $\pi(\chi \mid \text{rest})$ to denote the full conditional of parameter χ given all the remaining model parameters and the data. In an attempt to ease the notation in Sections 2.1–2.5, we make two simplifications. Firstly, we assume that there is only one outcome of each type i.e. one continuous, one binomial and one count outcome; therefore, we drop the d index. Secondly, we do all calculations assuming that the data on treated units $i > N_1$ and post-intervention times $t > T_i$ are not discarded. Nonetheless, it is trivial to perform all calculations in these sections without having to make the two simplifications.

B.1 Loadings parameters

We start with a brief overview of the SMMALA algorithm [Girolami and Calderhead, 2011]. Let $\pi(\chi \mid y)$ denote the target density, where χ is the parameter of interest and y are the data. Further, let ϵ be the stepsize. At each MCMC iteration, we propose new values χ^* from

$$\mathrm{N}\left(\chi + \frac{\epsilon^2}{2} G^{-1}(\chi) \nabla \log \pi(\chi \mid y), \epsilon^2 G^{-1}(\chi)\right),$$

where

$$G(\chi) = -E_{y|\chi}\left[\frac{\partial^2}{\partial \chi_i \partial \chi_j} \log \pi(\chi \mid y)\right],$$

and accept the new value with probability $\min\left\{1, \frac{\pi(\chi^* | y) q(\chi | \chi^*)}{\pi(\chi | y) q(\chi^* | \chi)}\right\}$, where $q(\cdot \mid \cdot)$ denotes the density of the normal proposal.

Our target density is $\pi(\boldsymbol{\lambda}_i \mid \text{rest})$. Using Equations 4.1 and 4.2 in the main paper, we have that

$$\pi(\boldsymbol{\lambda}_i \mid \text{rest}) \propto \mathrm{N}(\boldsymbol{\lambda}_i; \mathbf{0}, \boldsymbol{\Phi}_i) \prod_{t=1}^{T} \left[\mathrm{N}(y_{it}; \boldsymbol{\lambda}_i^\top \boldsymbol{f}_t + \boldsymbol{\eta}_1^\top \boldsymbol{x}_{it}, \sigma_i^2) \exp\left\{ -\frac{\omega_{it}}{2} \left(\frac{\kappa_{it}}{\omega_{it}} - \boldsymbol{\lambda}_i^\top \boldsymbol{g}_t - \boldsymbol{\eta}_2^\top \boldsymbol{x}_{it} \right)^2 \right\} \right.$$
$$\left. \mathrm{Pois}\left(L_{it}; \frac{w_{it} \exp\left(\boldsymbol{\lambda}_i^\top \boldsymbol{h}_t + \boldsymbol{\eta}_3^\top \boldsymbol{x}_{it}\right)}{\xi_i} \log(1 + \xi_i) \right) \right], \quad (8)$$

where $\boldsymbol{\Phi}_i = \text{diag}\{\phi_{i1}, \ldots, \phi_{iJ}\}$. Therefore,

$$\log \pi(\boldsymbol{\lambda}_i \mid \text{rest}) \propto -\sum_{j=1}^{J} \frac{\lambda_{ij}^2}{2\phi_{ij}} - \sum_{t=1}^{T} \frac{(y_{it} - \boldsymbol{\lambda}_i^\top \boldsymbol{f}_t - \boldsymbol{\eta}_1^\top \boldsymbol{x}_{it})^2}{2\sigma_i^2} - \sum_{t=1}^{T} \frac{\omega_{it}}{2} \left(\frac{\kappa_{it}}{\omega_{it}} - \boldsymbol{\lambda}_i^\top \boldsymbol{g}_t^\top - \boldsymbol{\eta}_2^\top \boldsymbol{x}_{it} \right)^2 -$$
$$\frac{\log(1 + \xi_i)}{\xi_i} \sum_{t=1}^{T} w_{it} \exp\left(\boldsymbol{\lambda}_i^\top \boldsymbol{h}_t + \boldsymbol{\eta}_3^\top \boldsymbol{x}_{it}\right) + \sum_{t=1}^{T} L_{it} \left(\boldsymbol{\lambda}_i^\top \boldsymbol{h}_t + \boldsymbol{\eta}_3^\top \boldsymbol{x}_{it}\right). \quad (9)$$

Thus, we obtain

$$\frac{\partial}{\partial \lambda_{ij}} \log \pi(\boldsymbol{\lambda}_i \mid \text{rest}) = -\frac{\lambda_{ij}}{\phi_{ij}} + \frac{1}{\sigma_i^2} \sum_{t=1}^{T} f_{tj} \left(y_{it} - \boldsymbol{\lambda}_i^\top \boldsymbol{f}_t - \boldsymbol{\eta}_1^\top \boldsymbol{x}_{it}\right)$$
$$+ \sum_{t=1}^{T} \omega_{it} g_{tj} \left(\frac{\kappa_{it}}{\omega_{it}} - \boldsymbol{\lambda}_i^\top \boldsymbol{g}_t^\top - \boldsymbol{\eta}_2^\top \boldsymbol{x}_{it} \right) + \frac{\log(1 + \xi_i)}{\xi_i} \sum_{t=1}^{T} w_{it} h_{tj} \exp\left(\boldsymbol{\lambda}_i^\top \boldsymbol{h}_t + \boldsymbol{\eta}_3^\top \boldsymbol{x}_{it}\right) + \sum_{t=1}^{T} L_{it} f_{tj}$$

Input : Total number of MCMC iterations B

for $b = 1$ *to* $b = B$ **do**

  `// Loadings parameters`

  Update ρ (Gibbs)

  For each loading j, update ζ_j (Gibbs)

  For each unit i and loading j, update ϕ_{ij} (Gibbs)

  For each unit i, update $\boldsymbol{\lambda}_i$ (SMMALA)

  `// Factor variance parameters`

  For each loading j, update M_j after marginalising out the $\{v_{j\ell}\}_{\ell=1}^{D}$ (Gibbs). Conditional on $M_j = l$, set $v_{jl} = 1$ and update $v_{j\ell}$ for $\ell \neq l$ (Gibbs).

  `// Normal outcome parameters`

  For each unit i and normal outcome d, update σ^2_{id} (Metropolis-Hastings)

  For each normal outcome d and time t, update $\boldsymbol{f}_{td}$ (Gibbs).

  For each normal outcome d, update $\boldsymbol{\eta}_{1,d}$ (Gibbs).

  `// Binomial outcome parameters`

  For each binomial outcome d, unit i and time t, update ω_{itd} (Gibbs).

  For each binomial outcome d and time t, update $\boldsymbol{g}_{td}$ (Gibbs).

  For each binomial outcome d, update $\boldsymbol{\eta}_{2,d}$ (Gibbs).

  `// Count outcome parameters`

  For each count outcome d and unit i, update ξ_{id} after marginalising out the $\{L_{itd}\}_{t=1}^{T}$ (Barker). Conditional on ξ_{id}, update the L_{itd} (Gibbs).

  For each count outcome d and time t, update $\boldsymbol{h}_{td}$ (SMMALA).

  For each count outcome d, update $\boldsymbol{\eta}_{3,d}$ (SMMALA).

  Save the current state of all model parameters to $\boldsymbol{\chi}^{(b)}$

end

Output: $\boldsymbol{\chi}^{(1)}, \ldots, \boldsymbol{\chi}^{(B)}$

Algorithm 1: Metropolis-within-Gibbs algorithm for the mixed outcome multivariate factor analysis model. The type of update in each step is indicated in parentheses.

and

$$\frac{\partial^2}{\partial \lambda_{ij} \partial \lambda_{is}} \log \pi \left(\boldsymbol{\lambda}_i \mid \text{rest} \right) = -\frac{1}{\phi_{ij}} \mathbb{I} \left(j = s \right) - \frac{1}{\sigma_i^2} \sum_{t=1}^{T} f_{tj} f_{ts} - \sum_{t=1}^{T} \omega_{it} g_{tj} g_{ts} + \frac{\log \left(1 + \xi_i \right)}{\xi_i} \sum_{t=1}^{T} w_{it} h_{tj} h_{ts} \exp \left(\boldsymbol{\lambda}_i^\top \boldsymbol{h}_t + \boldsymbol{\eta}_3^\top \boldsymbol{x}_{it} \right). \quad (10)$$

From Eq. (10), we deduce that

$$G(\boldsymbol{\lambda}_i) = \boldsymbol{\Phi}_i^{-1} + \frac{1}{\sigma_i^2}\sum_{t=1}^{T}\boldsymbol{f}_t\boldsymbol{f}_t^\top + \sum_{t=1}^{T}\omega_{it}\boldsymbol{g}_t\boldsymbol{g}_t^\top - \frac{\log(1+\xi_i)}{\xi_i}\sum_{t=1}^{T} w_{it}\exp\left(\boldsymbol{\lambda}_i^\top\boldsymbol{h}_t + \boldsymbol{\eta}_3^\top\boldsymbol{x}_{it}\right)\boldsymbol{h}_t\boldsymbol{h}_t^\top.$$

For the update of the loadings prior parameters ϕ_{ij}, ζ_j and ρ, we refer the reader to Gao et al. [2016] who present the full conditional distributions for these parameters.

B.2 Factor variance parameters

Recall that for the derivations in this section, we have assumed that $D_1 = D_2 = D_3 = 1$ and hence $D = 3$. Therefore, for all loadings j, $\boldsymbol{v}_j = (v_{j1}, v_{j2}, v_{j3})^\top$. Further, for all $\ell = 1, 2, 3$, $\Pr(M_j = \ell) = 1/3$. For each j, we have that

$$\begin{aligned}\pi\left(M_j = l, v_{j1}, v_{j2}, v_{j3} \mid \text{rest}\right) \propto \Pr\left(M_j = l\right)\prod_{\ell=1}^{D}\pi\left(v_{j\ell} \mid M_j = l\right) \\ \times \prod_{t=1}^{T}\left[\mathrm{N}\left(f_{tj}; 0, v_{j1}\right)\mathrm{N}\left(g_{tj}; 0, v_{j2}\right)\mathrm{N}\left(h_{tj}; 0, v_{j3}\right)\right] \quad (11)\end{aligned}$$

For the normal outcome, we have that

$$\begin{aligned}\prod_{t=1}^{T}\mathrm{N}\left(f_{tj}; 0, v_{j1}\right) &\propto \prod_{t=1}^{T} v_{j1}^{-1/2}\exp\left(-\frac{1}{2v_{j1}}f_{tj}^2\right) \\ &= v_{j1}^{-T/2}\exp\left(-\frac{1}{2v_{j1}}\sum_{t=1}^{T}f_{tj}^2\right) \\ &:= v_{j1}^{-a}\exp\left(-\frac{b_{j1}}{v_{j1}}\right). \quad (12)\end{aligned}$$

A similar calculation can be done for the terms involving g_{tj} and h_{tj} in (11). Let $b_{j2} = \frac{1}{2}\sum_{t=1}^{T} g_{tj}^2$ and $b_{j3} = \frac{1}{2}\sum_{t=1}^{T} h_{tj}^2$. We can rewrite (11) as

$$\begin{aligned}\pi\left(M_j = l, v_{j1}, v_{j2}, v_{j3} \mid \text{rest}\right) &\propto \exp\left(-b_{jl}\right)\prod_{\ell=1,\ell\neq l}^{D}\left[v_{j\ell}^{-a}\exp\left(-\frac{b_{j\ell}}{v_{j\ell}}\right)\mathbb{I}\left(v_{j\ell}^2 \in [0,1]\right)\right] \\ &\propto \frac{b_{jl}^{a-1}}{\Gamma(a-1)}1^{-a}\exp\left(-b_{jl}\right)\prod_{\ell=1,\ell\neq l}^{D}\left[\frac{b_{j\ell}^{a-1}}{\Gamma(a-1)}v_{j\ell}^{-a}\exp\left(-\frac{b_{j\ell}}{v_{j\ell}}\right)\mathbb{I}\left(v_{j\ell} \in [0,1]\right)\right] \\ &= \phi_{\mathrm{IG}}\left(1; a-1, b_{jl}\right)\prod_{\ell=1,\ell\neq l}^{D}\phi_{\mathrm{IG}}\left(v_{j\ell}; a-1, b_{j\ell}\right)\mathbb{I}\left(v_{j\ell} \in [0,1]\right), \quad (13)\end{aligned}$$

where $\phi_{\text{IG}}(a; b, c)$ is the probability density function of an inverse-gamma distribution with parameters b and c, evaluated at a. It is now easy to obtain

$$\begin{aligned}
\Pr(M_j = l \mid \text{rest}) &= \int_{v_{j1}} \int_{v_{j2}} \int_{v_{j3}} \pi(M_j = l, v_{j1}, v_{j2}, v_{j3} \mid \text{rest})\, dv_{j3} dv_{j2} dv_{j1} \\
&\propto \phi_{\text{IG}}(1; a-1, b_{jl}) \prod_{\ell=1, \ell \neq l}^{D} \Phi_{\text{IG}}(1; a-1, b_{j\ell}), \qquad (14)
\end{aligned}$$

where $\Phi_{\text{IG}}(a; b, c)$ is the cumulative density function of an inverse-gamma distribution with parameters b and c, evaluated at a. Sampling from (14) is straightforward as l can only take D possible values. Conditional on $M_j = 1$, we set $v_{jl} = 1$. If $M_j \neq 1$, we have that (using (12))

$$\pi(v_{j1} \mid \text{rest}, M_j \neq 1) \propto v_{j1}^{-a} \exp\left(-\frac{b_{j1}}{v_{j1}}\right) \mathbb{I}(v_{j1} \in [0,1]),$$

which implies that we can draw v_{j1} from an $\text{IG}(a-1, b_{j1})$ truncated in $[0, 1]$.

B.3 Normal outcome parameters

Recall that $\mu_{it} = \boldsymbol{\lambda}_i^\top \boldsymbol{f}_t + \boldsymbol{\eta}_1^\top \boldsymbol{x}_{it}$. For each unit i, the full conditional of σ_i^2 is

$$\begin{aligned}
\pi\left(\sigma_i^2 \mid \text{rest}\right) &\propto \pi(\sigma_i^2) \prod_{t=1}^{T} \text{N}(y_{it}; \mu_{it}, \sigma_i^2) \\
&\propto \mathbb{I}\left(\sigma_i^2 \in [0, 10^2]\right) \left(\sigma_i^2\right)^{-T/2} \exp\left(-\frac{1}{2\sigma_i^2} \sum_{t=1}^{T} (y_{it} - \mu_{it})^2\right) \\
&\propto \mathbb{I}\left(\sigma_i^2 \in [0, 10^2]\right) \text{Gamma}\left(\sigma_i^2; \frac{T}{2} + 1, \frac{1}{2} \sum_{t=1}^{T} (y_{it} - \mu_{it})^2\right).
\end{aligned}$$

We propose a new value $\sigma_i^{2*} \sim \text{Gamma}\left(\frac{T}{2} + 1, \frac{1}{2} \sum_{t=1}^{T} (y_{it} - \mu_{it})^2\right)$. The Metropolis-Hastings ratio simplifies to $\mathbb{I}\left(\sigma_i^{2*} \in [0, 10^2]\right)$. Therefore, we accept the proposed value if it lies within the prior support of the uniform distribution. The full conditional distributions of $\boldsymbol{f}_t$ and $\boldsymbol{\eta}_1$ are straightforward to derive, see for example Section A in Samartsidis et al. [2020].

B.4 Binomial outcome parameters

Let $\tilde{y}_{it} = \frac{\kappa_{it}}{\omega_{it}} - \boldsymbol{\eta}_2^\top \boldsymbol{x}_{it}$. From Equation 4.1 in the main paper, we have that for each t

$$\begin{aligned}
\pi\left(\boldsymbol{g}_t \mid \text{rest}\right) &\propto \text{N}\left(\boldsymbol{g}_t; \mathbf{0}, \boldsymbol{S}_2\right) \prod_{i=1}^{N} \exp\left(-\frac{\omega_{it}}{2}\left(\tilde{y}_{it} - \boldsymbol{\lambda}_i^\top \boldsymbol{g}_t\right)^2\right) \\
&\propto \exp\left(-\frac{1}{2}\left(\boldsymbol{g}_t^\top \boldsymbol{S}_2^{-1} \boldsymbol{g}_t\right) - \frac{1}{2}\left(\tilde{\boldsymbol{Y}}_t - \boldsymbol{\Lambda} \boldsymbol{g}_t\right)^\top \boldsymbol{\Omega}_t \left(\tilde{\boldsymbol{Y}}_t - \boldsymbol{\Lambda} \boldsymbol{g}_t\right)\right) \\
&\propto \exp\left(\tilde{\boldsymbol{Y}}_t^\top \boldsymbol{\Omega}_t \boldsymbol{\Lambda} \boldsymbol{g}_t - \frac{1}{2} \boldsymbol{g}_t^\top \left(\boldsymbol{S}_2^{-1} + \boldsymbol{\Lambda}^\top \boldsymbol{\Omega}_t \boldsymbol{\Lambda}\right) \boldsymbol{g}_t\right),
\end{aligned}$$

where $\boldsymbol{S}_2 = \text{diag}\left\{s_{2,1}, \ldots, s_{2,J}\right\}$, $\tilde{\boldsymbol{Y}}_t = \left(\tilde{y}_{1t}, \ldots, \tilde{y}_{Nt}\right)^\top$ and $\boldsymbol{\Omega}_t = \text{diag}\left\{\omega_{1t}, \ldots, \omega_{Nt}\right\}$. Therefore, we draw $\boldsymbol{g}_t$ from a normal distribution with variance-covariance matrix $\boldsymbol{Q} = \left(\boldsymbol{S}_2^{-1} + \boldsymbol{\Lambda}^\top \boldsymbol{\Omega}_t \boldsymbol{\Lambda}\right)^{-1}$ and mean $\boldsymbol{Q} \boldsymbol{\Lambda}^\top \boldsymbol{\Omega}_t \tilde{\boldsymbol{Y}}_t$. The derivation of the full conditional of $\boldsymbol{\eta}_2$ is analogous and therefore not shown.

B.5 Negative Binomial outcome parameters

Recall that $q_{it} = \exp\left(\boldsymbol{\lambda}_i^\top \boldsymbol{h}_t + \boldsymbol{\eta}_3^\top \boldsymbol{x}_{it}\right)$. For each unit i, we have from Equation 4.2 in the main paper that

$$\pi\left(\xi_i, \{L_{it}\}_{t=1}^T \mid \text{rest}\right) \propto \mathbb{I}\left(\xi_i \in [0, 20]\right) \prod_{t=1}^{T} \left[\text{CRT}\left(L_{it}; z_{it}, \frac{w_{it} q_{it}}{\xi_i}\right) \text{NegBin}\left(z_{it}; \frac{w_{it} q_{it}}{\xi_i}, \frac{1}{1 + \xi_i}\right)\right]. \quad (15)$$

It is straightforward to integrate out L_{it} from (15)

$$\begin{aligned}
\pi\left(\xi_i \mid \text{rest} \setminus \{L_{it}\}_{t=1}^T\right) &= \sum_{L_{i1}=0}^{\infty} \cdots \sum_{L_{iT}=0}^{\infty} \left[\pi\left(\xi_i, \{L_{it}\}_{t=1}^T \mid \text{rest}\right)\right] \\
&\propto \mathbb{I}\left(\xi_i \in [0, 20]\right) \prod_{t=1}^{T} \left[\text{NegBin}\left(z_{it}; \frac{w_{it} q_{it}}{\xi_i}, \frac{1}{1 + \xi_i}\right) \sum_{L_{it}=0}^{\infty} \text{CRT}\left(L_{it}; z_{it}, \frac{w_{it} q_{it}}{\xi_i}\right)\right] \\
&= \mathbb{I}\left(\xi_i \in [0, 20]\right) \prod_{t=1}^{T} \text{NegBin}\left(z_{it}; \frac{w_{it} q_{it}}{\xi_i}, \frac{1}{1 + \xi_i}\right). \quad (16)
\end{aligned}$$

We draw ξ_i from (16) using the Barker method [Livingstone and Zanella, 2022]. In our simulation studies, we found that drawing ξ_i from (16) leads to much better mixing for these parameters compared to drawing them from $\pi\left(\xi_i \mid \text{rest}\right)$ (i.e. their full conditional).

The remaining negative binomial parameters $\boldsymbol{h}_t$ and $\boldsymbol{\eta}_3$ are updated using the SMMALA algorithm. The derivations of the gradients and tensor matrices follow the ones done in Section B.1 and therefore are not shown.

C Supplement to simulation studies

C.1 Simulation study 1: additional results

In this section, we provide supplementary results for the simulation study of Section 5 of the main paper. The bias, standard error of point estimates, credible interval width and false positive rate of the $\mathcal{C}_i = \{\alpha_i, \beta_i, \gamma_i, \delta_i\}$ obtained in scenario $\ell = 1$ (no intervention effect) are presented in Table C.1. Figure C.1 shows the power of detecting a non-zero effect on the $\mathcal{C}_i$, for different values of T_i.

Table C.1: Simulation results for scenario $\ell = 1$ (no intervention effect) in **simulation study 1**. The table presents the bias of the point estimates of $\mathcal{C}_i = \{\alpha_i, \beta_i, \gamma_i, \delta_i\}$, the standard error of the point estimates, the width of the 95% credible intervals and the false positive rates. The results are based on 2,500 simulated datasets.

Bias of point estimates

	α_i		β_i		γ_i		δ_i	
T_i	UV	MV	UV	MV	UV	MV	UV	MV
any	0.059	0.059	0.011	-0.006	1.293	-0.412	0.447	-1.226
8	0.125	0.141	0.034	-0.005	3.517	-0.091	0.051	-0.357
16	0.028	0.025	0.006	-0.005	0.725	-0.371	0.258	-0.958
23	-0.008	-0.004	-0.005	-0.006	-0.670	-0.745	-2.121	-1.694

Standard error of point estimates

	α_i		β_i		γ_i		δ_i	
T_i	UV	MV	UV	MV	UV	MV	UV	MV
any	0.668	0.499	0.066	0.041	6.858	4.294	50.673	26.956
8	0.860	0.614	0.080	0.048	7.591	4.480	108.728	36.300
16	0.544	0.403	0.054	0.034	5.989	3.767	30.309	20.879
23	0.755	0.695	0.061	0.054	7.482	6.640	28.382	26.593

Credible interval width

	α_i		β_i		γ_i		δ_i	
T_i	UV	MV	UV	MV	UV	MV	UV	MV
any	2.631	1.972	0.226	0.146	22.217	14.480	123.292	85.012
8	3.521	2.445	0.292	0.165	25.367	14.177	197.629	114.272
16	2.113	1.625	0.189	0.126	19.827	13.071	95.073	69.826
23	3.317	3.101	0.244	0.219	28.960	26.109	109.225	103.251

False positive rate

	α_i		β_i		γ_i		δ_i	
T_i	UV	MV	UV	MV	UV	MV	UV	MV

any	0.050	0.049	0.078	0.059	0.085	0.061	0.072	0.058
8	0.046	0.049	0.089	0.066	0.105	0.070	0.086	0.059
16	0.053	0.052	0.075	0.058	0.077	0.061	0.067	0.057
23	0.037	0.033	0.049	0.047	0.043	0.039	0.050	0.050

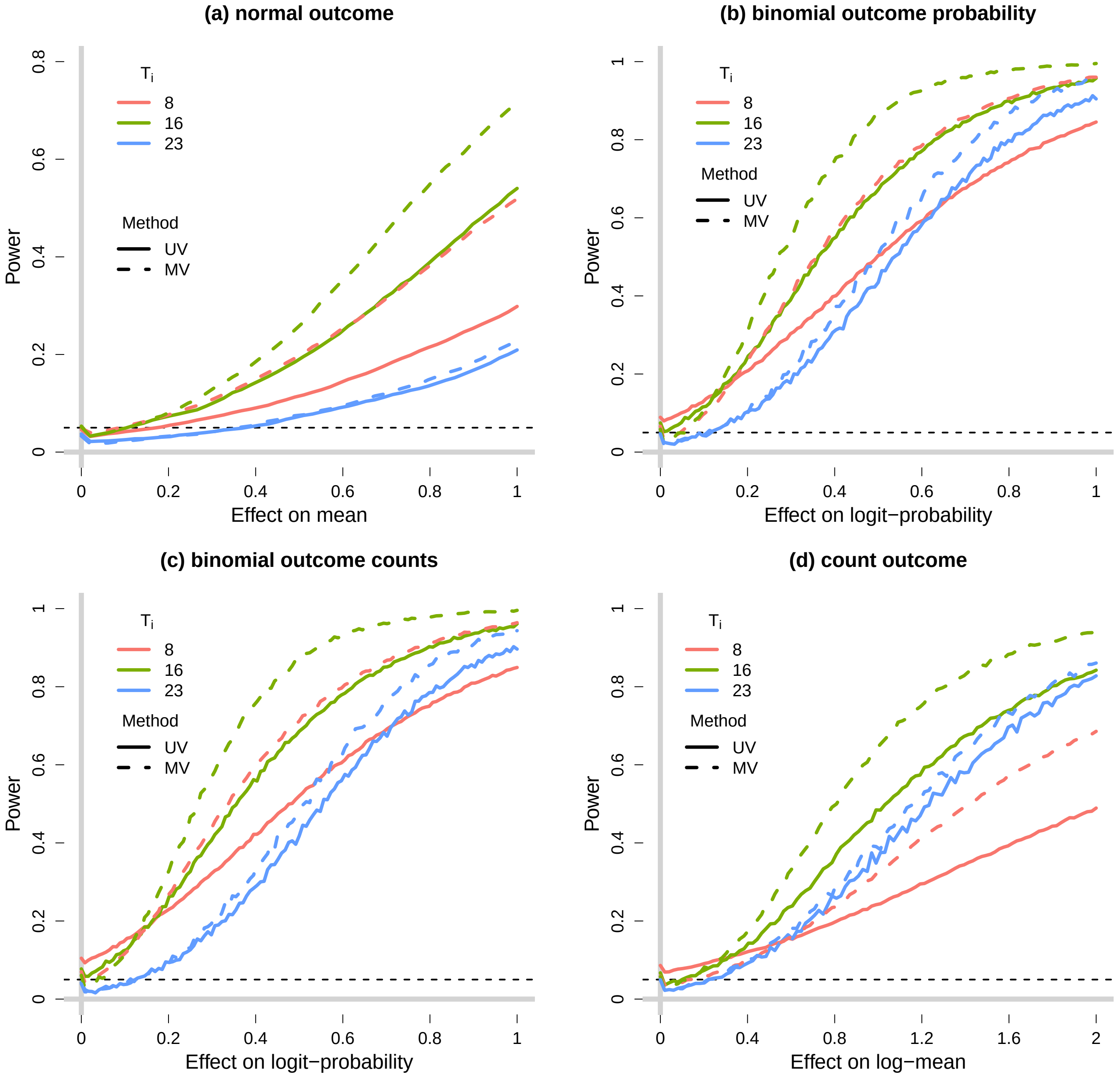

Figure C.1: Power of detecting an intervention effect for a randomly chosen treated unit (y-axis) as a function of the magnitude of the intervention effect (x-axis) in **simulation study 1**. Panels (a)-(d) correspond to causal effects α_i, β_i, γ_i and δ_i, respectively. The results are based on 2,500 simulated datasets.

C.2 Simulation study 2

We repeat the simulation study of Section 5 after changing some of the simulation parameters. Here, we consider a setting with a limited number of units but with longer time-series. In particular, we set $N = 40$ units, $T = 48$ time points thus keeping the total amount of data fixed. We let $t_{\min} = 16$. For each outcome and loading j, the factors follow a random walk process with standard deviations as in Table 1 of the main paper. The treated units are chosen as in simulation study 1 i.e. using Equation (5.9) of the main paper. Here, we choose κ_0 so that there are $N_2 = 20$ treated units on average (over simulated datasets). The value of κ_1 is set such that the average (over simulated datasets) value of the empirical completion probability k_{it}/n_{it} is roughly 10% higher in controls units for all $t > t_{\min}$, i.e. units with lower success probability are more likely to be treated. Similar, κ_2 is chosen such that on average (over simulated datasets), the value of z_{it}/w_{it} is roughly 10% higher in controls units for every $t > t_{\min}$.

We summarise results similar to simulation study 1. Table C.2 shows bias, standard errors, credible interval width and false positive rates of the point estimates of α_i, β_i, γ_i and δ_i in scenario $\ell = 1$ of no intervention effect. Figure C.2 shows power as a function of the intervention effect magnitude for a randomly selected unit. Figure C.3 presents power as a function of the intervention effect magnitude, for different values of T_i. We again see that the multivariate approach outperforms the univariate approach offering better control of false positive rates and statistical efficiency when the number of pre-intervention time point is either small ($T_i = 16$) or moderate $T_i = 32$.

Table C.2: Simulation results for scenario $\ell = 1$ (no intervention effect) in **simulation study 2**. The table presents the bias of the point estimates of $\mathcal{C}_i = \{\alpha_i, \beta_i, \gamma_i, \delta_i\}$, the standard error of the point estimates, the width of the 95% credible intervals and the false positive rates. The results are based on 2,500 simulated datasets.

**Bias of point estimates**								
	α_i		β_i		γ_i		δ_i	
T_i	UV	MV	UV	MV	UV	MV	UV	MV
any	0.009	0.024	-0.002	-0.002	-0.158	-0.140	0.789	1.666
16	0.014	0.066	-0.005	-0.002	-0.391	-0.087	-1.989	5.422
32	0.004	0.012	-0.001	-0.002	-0.037	-0.112	2.087	0.966
47	-0.024	-0.010	-0.002	-0.001	-0.205	-0.145	0.563	0.893

**Standard error of point estimates**								
	α_i		β_i		γ_i		δ_i	
T_i	UV	MV	UV	MV	UV	MV	UV	MV

any	0.761	0.659	0.069	0.047	7.178	4.935	61.712	45.687
16	1.211	1.016	0.105	0.076	10.418	7.489	92.276	73.096
32	0.562	0.482	0.055	0.036	6.132	3.922	40.343	30.195
47	0.774	0.775	0.051	0.049	6.009	5.865	41.998	38.293

Credible interval width

	α_i		β_i		γ_i		δ_i	
T_i	UV	MV	UV	MV	UV	MV	UV	MV
any	2.540	2.167	0.188	0.141	18.655	14.023	119.932	87.915
16	4.259	3.460	0.304	0.225	27.465	20.186	205.381	129.357
32	1.985	1.703	0.152	0.112	15.884	11.593	91.731	68.320
47	3.121	3.085	0.188	0.183	22.204	21.577	125.477	123.395

False positive rate

	α_i		β_i		γ_i		δ_i	
T_i	UV	MV	UV	MV	UV	MV	UV	MV
any	0.063	0.068	0.112	0.083	0.120	0.086	0.109	0.089
16	0.064	0.078	0.124	0.098	0.143	0.116	0.121	0.089
32	0.063	0.069	0.120	0.082	0.125	0.078	0.117	0.093
47	0.045	0.041	0.049	0.050	0.036	0.038	0.050	0.046

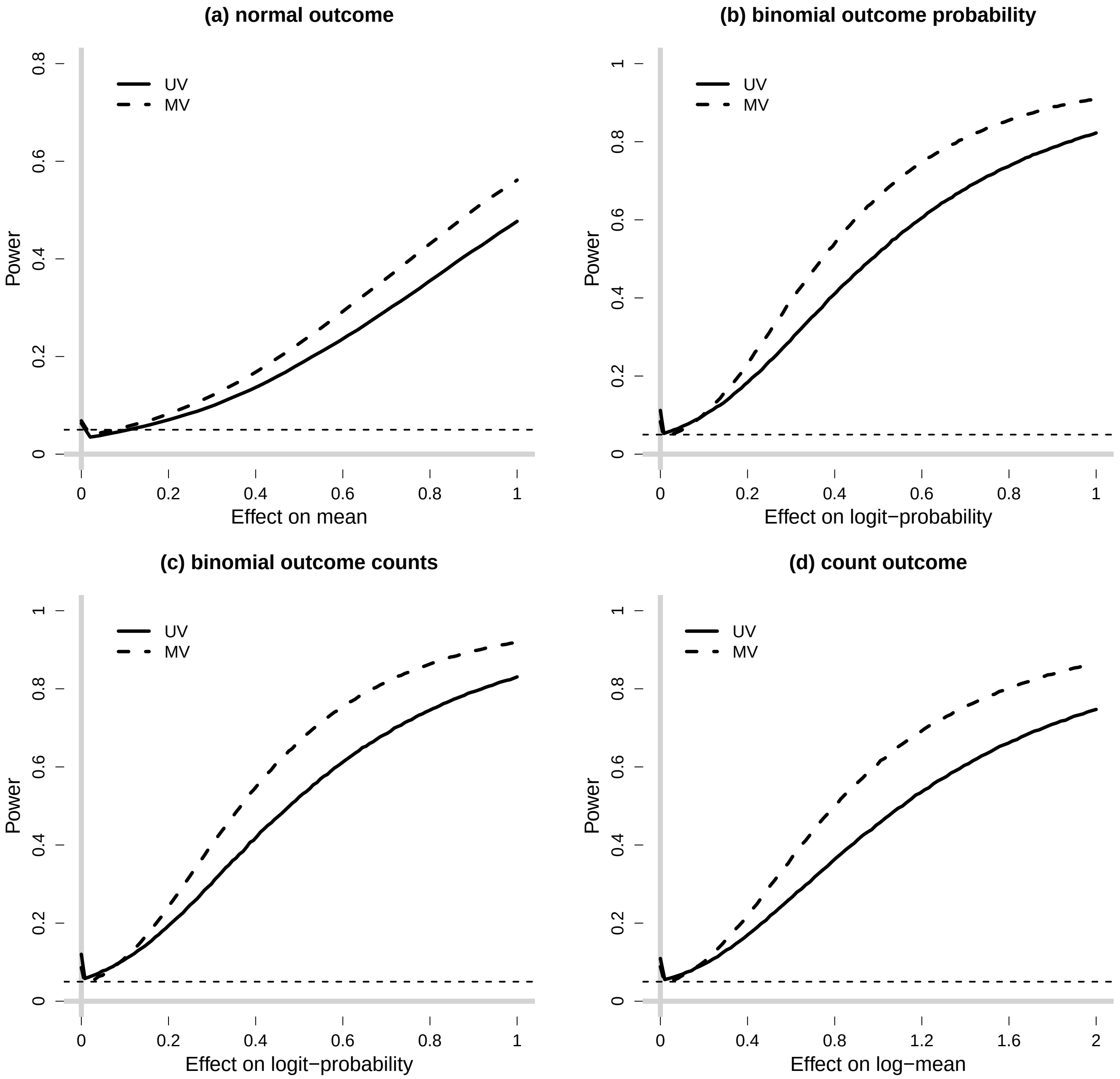

Figure C.2: Power of detecting an intervention effect for a randomly chosen treated unit (y-axis) as a function of the magnitude of the intervention effect (x-axis) in **simulation study 2**. Panels (a)-(d) correspond to causal effects α_i, β_i, γ_i and δ_i, respectively. The results are based on 2,500 simulated datasets.

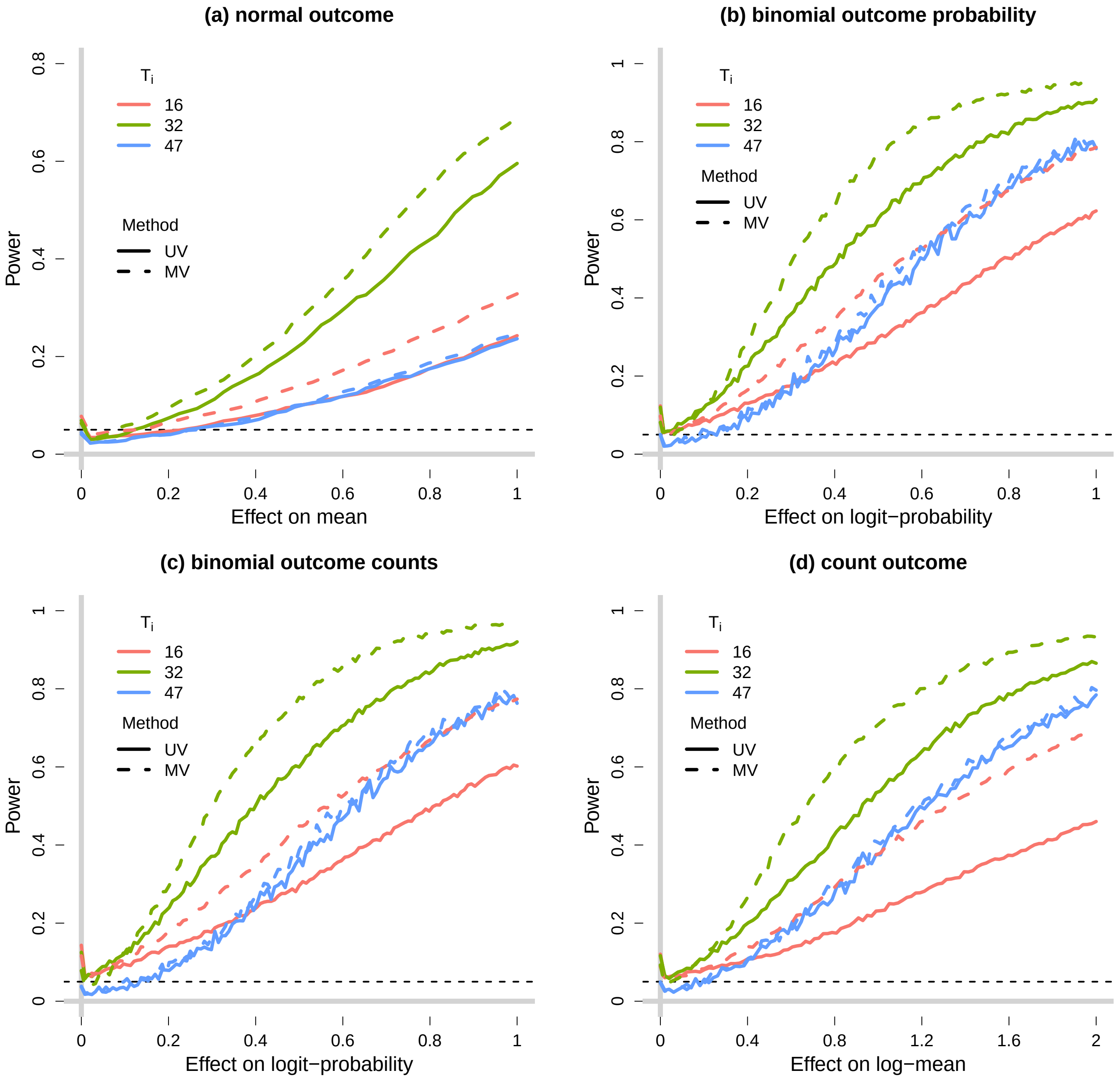

Figure C.3: Power of detecting an intervention effect for a randomly chosen treated unit with fixed T_i (y-axis) as a function of the magnitude of the intervention effect (x-axis) in **simulation study 2**. Panels (a)-(d) correspond to causal effects α_i, β_i, γ_i and δ_i, respectively. The results are based on 2,500 simulated datasets.

C.3 Simulation study 3

In our final simulation study we evaluated the performance of the multivariate FA model on datasets that resemble, as closely as possible, the LTP data introduced in Section 2 of the paper. We generated $B = 1,000$ simulated datasets from the (multivariate) FA model as follows.

Many of the parameters used to simulate the data are the same as those in the LTP data. Specifically, we set $D_1 = 0$, $D_2 = 3$, $D_3 = 1$, $N = 181$, $N_1 = 63$, $T = 138$, and we used the same n_{itd} (number of binomial trials) and w_{it} (offset for the count outcome — we omit the subscript d on w_{itd} because there is only one count outcome) that we have in the LTP data. The same values of all these quantities were used for all B simulated datasets. We now describe how we generated simulated dataset b (for a given $b = 1, \dots, B$).

First, we took the realisation of the unknown random variables $\big\{(p_{itd}, q_{it}, \xi) : i = 1, \dots, N; t = 1, \dots, T; d = 1, 2, 3\big\}$ obtained at the bth iteration (after thinning the chain) of our MCMC algorithm when it was applied to the real LTP data. This realisation represents a sample from the posterior distribution of these random variables. Potential untreated outcomes $k_{itd}^{(0)}$ and $z_{itd}^{(0)}$ for all units $i = 1, \dots, 181$ and all times $t = 1, \dots, 138$ were then generated from the FA model (equation (3.1) in the paper) conditional on these values of p_{itd}, q_{it} and ξ. By using this approach, we ensure that the temporal trends and relationships between the four outcomes that are seen in the LTP data were preserved in the simulated data.

The identity of the $N_2 = 118$ treated units and the last times at which these treated units are untreated, T_i, were then randomly sampled as follows. Let T_i^{LTP} ($i = 1, \dots, 181$) denote the value of T_i in the real LTP data (with $T_i^{\text{LTP}} = 181$ for the 63 untreated units), and let $m_t = \sum_{i=1}^{181} \mathbb{I}\left(T_i^{\text{LTP}} + 1 = t\right)$ ($t = 1, \dots, 138$) denote the number of units starting treatment at time t. For the bth simulated dataset, we sample m_t units to start treatment at time t from the set $\mathcal{U}_t$ of available (i.e. not yet treated) units at time t, with sampling weights

$$\varpi_{it,\ell} \propto \sum_{j \in \mathcal{U}_t} \left[\mathbb{I}\left(\tilde{n}_{it} > \tilde{n}_{jt}\right) + \mathbb{I}\left(\tilde{p}_{it1} \leq \tilde{p}_{jt1}\right) + \mathbb{I}\left(\tilde{q}_{it} \leq \tilde{q}_{jt}\right)\right],$$

where $\tilde{n}_{it} = \sum_{s=1}^{t-1} n_{is}/(t-1)$ ($\tilde{p}_{it1}$ and $\tilde{q}_{it}$ are defined analogously). This means that units with a high average number of cases, or a low average case completion rate, or a low number of contacts per completed case are more likely to be selected. Figure C.4 shows the proportion of the $B = 1000$ simulated datasets in which each of the 181 units was a control unit. We see that some units were more likely to be treated than others.

Once the treated units and their treatment times had been determined, we generated the post-treatment outcomes of these treated units as follows. We begin with the first binomial outcome (i.e. case completion). First, for $i > N_1$ and $t > T_i^{\text{LTP}}$ we calculate $\tilde{\beta}_{it1} = k_{it1}^{\text{LTP}}/n_{it1}^{\text{LTP}} - p_{it1}$ where $k_{it1}^{\text{LTP}}/n_{it1}^{\text{LTP}}$ is the value of k_{it1}/n_{it1} that we observe in the LTP data. The $\tilde{\alpha}_{it1}$'s are representative of the effects that we estimated from the LTP data, and are thus characterised by large heterogeneity. Second, we calculated the sample mean and variance of the $\tilde{\alpha}_{it1}$'s;

denote these by $\mu_{\tilde{\beta}}$ and $\sigma^2_{\tilde{\beta}}$, respectively. Third, for each $i > N_1$ and $t > T_i$, we set $p^{(T_i)}_{it1} = p_{it1} + \beta_{it1}$, where $\alpha_{it1} \sim \text{Normal}\left(\mu_{\tilde{\beta}}, \sigma^2_{\tilde{\beta}}\right)$. If this leads to a value of $p^{(T_i)}_{it1}$ that is not within $[0, 1]$, we re-draw α_{it1} until that condition is satisfied. Fourth, we draw $k_{it1} \sim \text{Binomial}\left(n_{it1}, p^{(T_i)}_{it1}\right)$. The post-treatment data are generated for the three remaining outcomes in a similar fashion.

The simulation of dataset b is now complete. To analyse this simulated dataset, we set $J^* = 20$, ran MCMC for 75,000 iterations, discarded the first 25,000 as a burn-in, and thinned the remaining 50,000 every 25 iterations to obtain 2,000 draws from the posterior. For each $i > N_1$ and $t > T_i$ we calculated the point estimates (posterior means) and 95% posterior CIs of β_{it1}. Based on these we calculated the error $e^{\beta_1}_{it} = \hat{\beta}_{it1} - \beta_{it1}$ and $o^{\beta_1}_{it} = \mathbb{I}\left(\beta_{itd} \in [\beta^{\mathrm{L}}_{itd}, \beta^{\mathrm{U}}_{itd}]\right)$, where β^{L}_{itd} and β^{U}_{itd} are the lower and upper limits of the 95% posterior CI. Finally, we repeated this for β_{it2}, β_{it3}, γ_{it1}, γ_{it2}, γ_{it3} and δ_{it}.

Having carried out this procedure for all $B = 1,000$ simulations, we have B values of $e^{\beta_1}_{it}$ and $o^{\beta_1}_{it}$ (and similarly for the e_{it}'s and o_{it}'s corresponding to each of β_{it2}, β_{it3}, γ_{it1}, γ_{it2}, γ_{it3} and δ_{it}). We shall add a subscript b to these e_{it}'s and o_{it}'s to indicate the simulation number ($b = 1, \ldots, B$). Next, for each unit $i = 1, \ldots, N$ and time $t = 1, \ldots, T$, let B_{it} denote the number of simulated datasets in which unit i was treated at time t. When $B_{it} > 0$, we can calculate the 'bias' and 'coverage' for β_{it1} (and similarly for the remaining estimands) as $\sum_{b=1}^{B} e^{\beta_1}_{it,b}/B_{it}$ and $\sum_{i=1}^{B} o^{\beta_1}_{it,b}/B_{it}$, respectively, where $e^{\beta_1}_{it,b} = o^{\beta_1}_{it,b} = 0$ if unit i was not treated at time t in simulated dataset b.

We show results for β_{itd} ($d = 1, 2, 3$) and δ_{it}. Histograms of bias and coverage are shown in Figures C.5 and C.6, respectively, where we have removed data on combinations of i and t with $B_{it} < 500$. In terms of bias, we generally see that the values are centered at zero in all four outcomes, meaning that overall our point estimates neither over-estimated nor under-estimated the causal effects. The coverage is around 95% for all outcomes (slightly less for contact completion), meaning that the CIs provided by our method included the true values in the majority of simulations. Hence, we conclude that overall our method performs well in this challenging scenario.

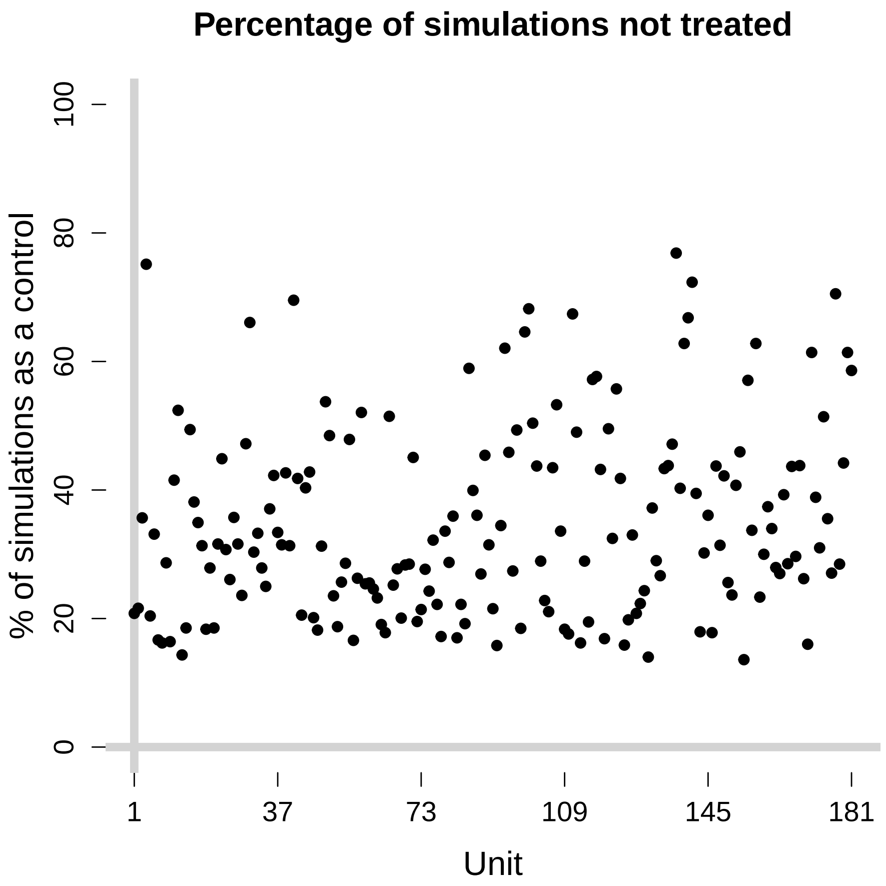

Figure C.4: Percentage of simulations out of 1,000 that each unit was a control unit in **simulation study 3**.

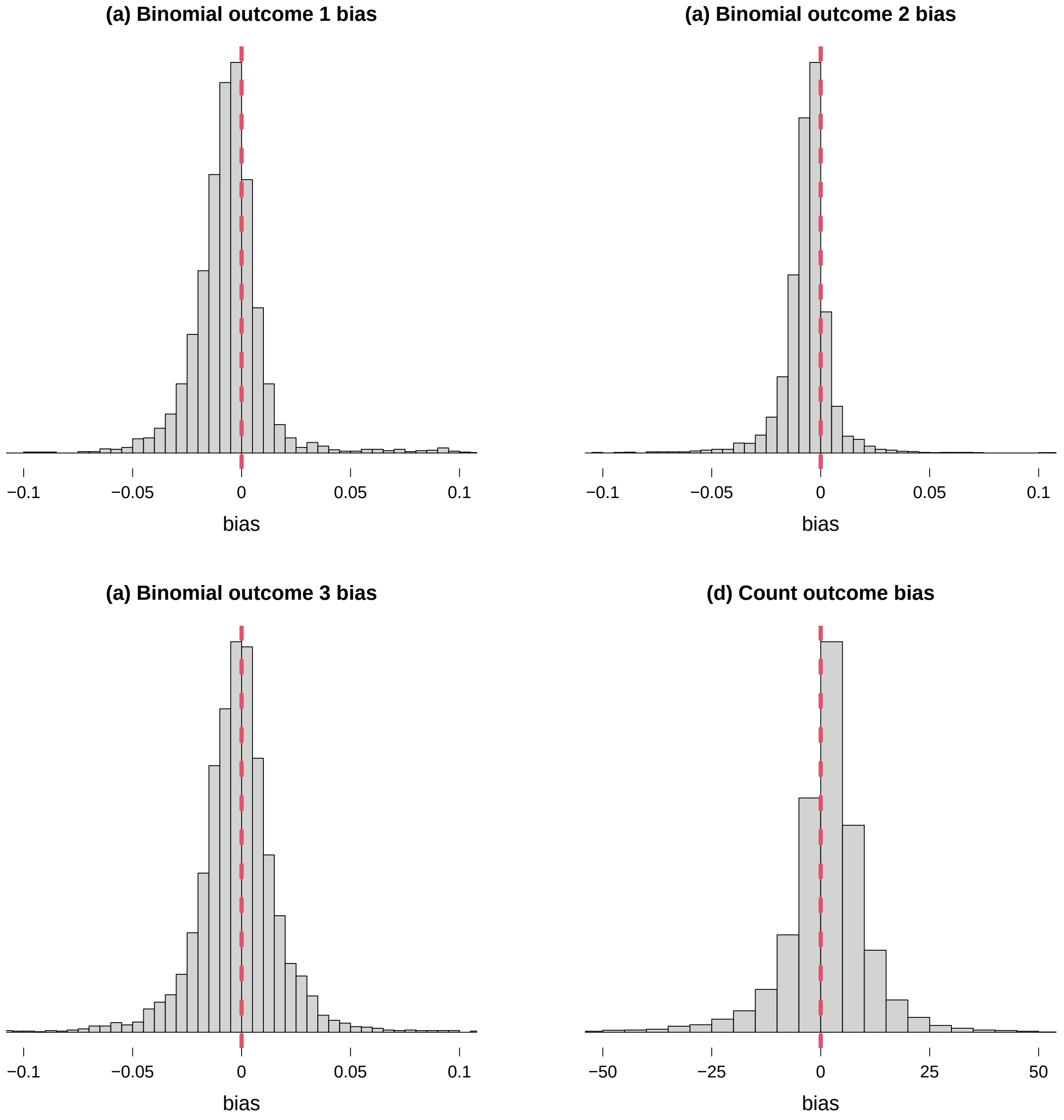

Figure C.5: Histogram of 'bias' in the estimates of β_{it1} (panel a), β_{it2} (panel b), β_{it3} (panel c) and δ_{it} (panel d) in **simulation study 3**. Combinations of i and t for which $B_{it} < 500$ have been excluded from the plot.

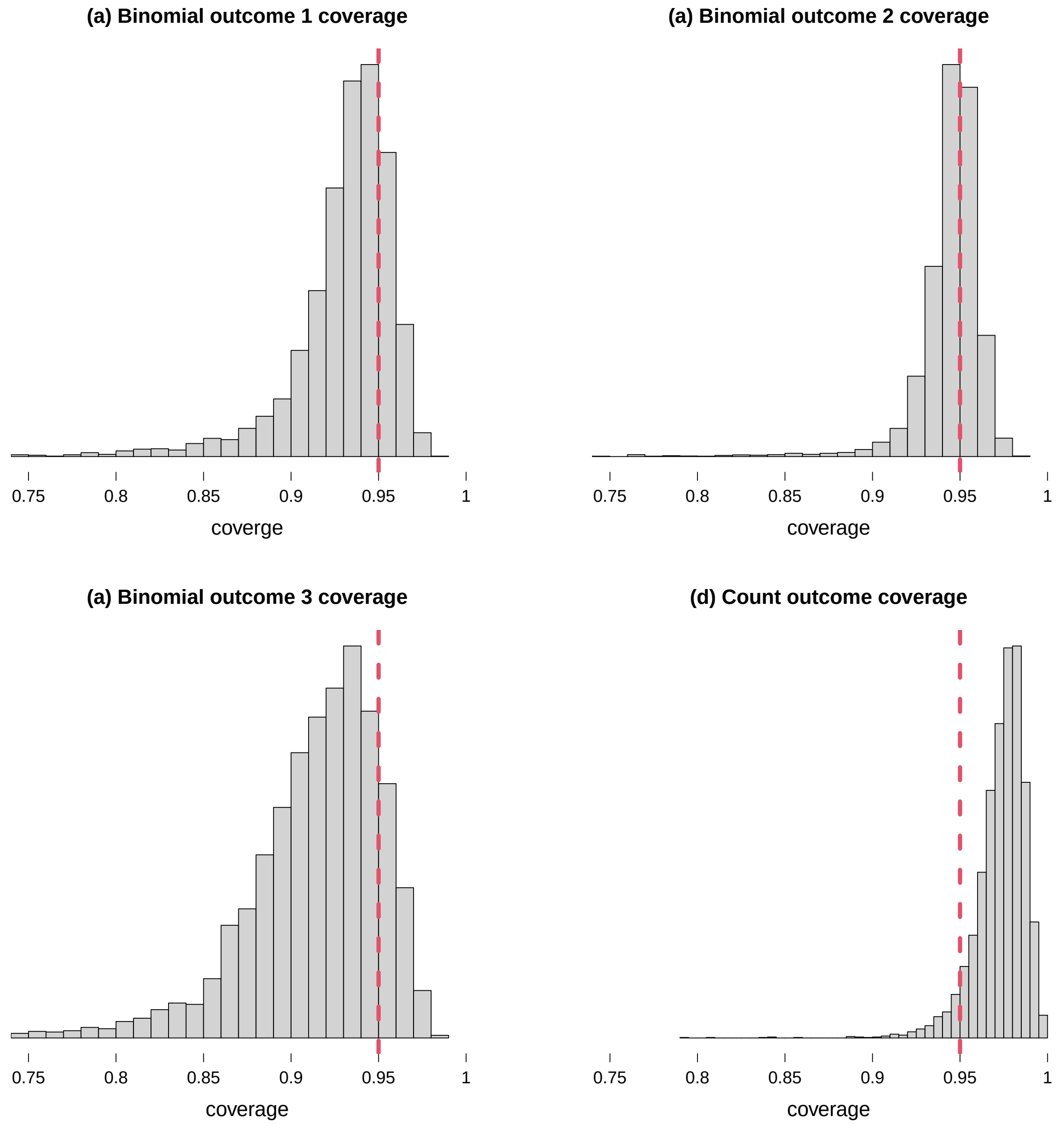

Figure C.6: Histogram of the coverage of 95% CIs of β_{it1} (panel a), β_{it2} (panel b), β_{it3} (panel c) and δ_{it} (panel d) in **simulation study 3**. Combinations of i and t for which $B_{it} < 500$ have been excluded from the plot.

D Supplement to LTP data analysis

In this Section we provide supplementary plots and results for the LTP application introduced in Section 2 of the main paper. Table D.1 shows he estimated average effects over all units and post-intervention time points, β_d, γ_d ($d = 1, 2, 3$) and δ_1. Figure D.1 shows summaries of the four outcomes. Figures D.2, D.3 and D.4 show summaries (similar to Figure 4 of the main paper) of the LTP data analysis described in Section 6 of the main paper, for outcomes timely completion, contact completion and number of contacts, respectively. Figure D.5 presents the point estimates (obtained as the posterior mean) of the causal effects γ_{it1}, γ_{itd2} and γ_{itd3}. The posterior distributions of ranks $r^{(\beta)}_{\iota t1}$, $r^{(\beta)}_{\iota t2}$, $r^{(\beta)}_{\iota t3}$ and $r^{(\delta)}_{\iota t1}$, where ι is the unit that we used in Section 6 of the main paper for illustration, are summarised in Figure D.6.

We perform four sensitivity analyses. For each one, we repeat the real analysis of Section 6 of the main paper with the same MCMC specifications, changing some of the hyper-parameter values or model specifications. We do this to investigate the effect that these have on the conclusions that we draw regarding the effectiveness of LTPs. In the first two, we set $J^* = 15$ and $J^* = 35$, respectively. In the third one, we set $\nu = 1$. Finally, in the last one we do not introduce the variables M_j but instead let the factor variance terms $v_{jl} \sim \text{Uni}(0, 1)$ for all $j = 1, \ldots, J^*$ and all $\ell = 1, \ldots, D$.

For each one of the four sensitivity analyses considered, we plot the point estimates and width of 95% credible of all causal estimands obtained, against the corresponding values obtained from our original analysis of Section 6 in the main paper. Here, we show this comparison for estimands β_{it1} (Fig. D.7 for point estimates and D.8 for CIs), γ_{it1} (Fig. D.9 for point estimates and D.10 for CIs) and δ_{it1} (Fig. D.11 for point estimates and D.12 for CIs) to reduce the amount of figures. In all plots, we see that both the point estimates and width of CIs are very similar. Thus, we conclude that our results are not sensitive to the change in specifications listed above. However, this might not necessarily be the case in other real datasets.

Table D.1: Posterior mean along with 95% CIs for the 'overall' effects β_d ($d = 1, 2, 3$), γ_d ($d = 1, 2, 3$) and δ_1. We present overall effects for the entire study period, as well as the period 01/10/2020-15/11/2020.

		Whole period		10/2020 onward	
Outcome	Effect	Estimate	95% CI	Estimate	95% CI
Completion	β_1	0.013	[0.006,0.021]	0.044	[0.038,0.051]
	γ_1	3.612	[3.015,4.262]	5.734	[4.885,6.618]
Timeliness	β_2	-0.074	[-0.080,-0.068]	0.014	[0.007,0.020]
	γ_2	0.218	[-0.221,0.660]	1.973	[1.367,2.602]
Contact completion	β_3	-0.014	[-0.022,-0.007]	-0.007	[-0.015,0.001]
	γ_3	-1.992	[-3.743,-0.439]	-2.416	[-4.746,-0.411]
Number of contacts	δ_1	-0.552	[-7.852,4.893]	-0.994	[-10.870,6.480]

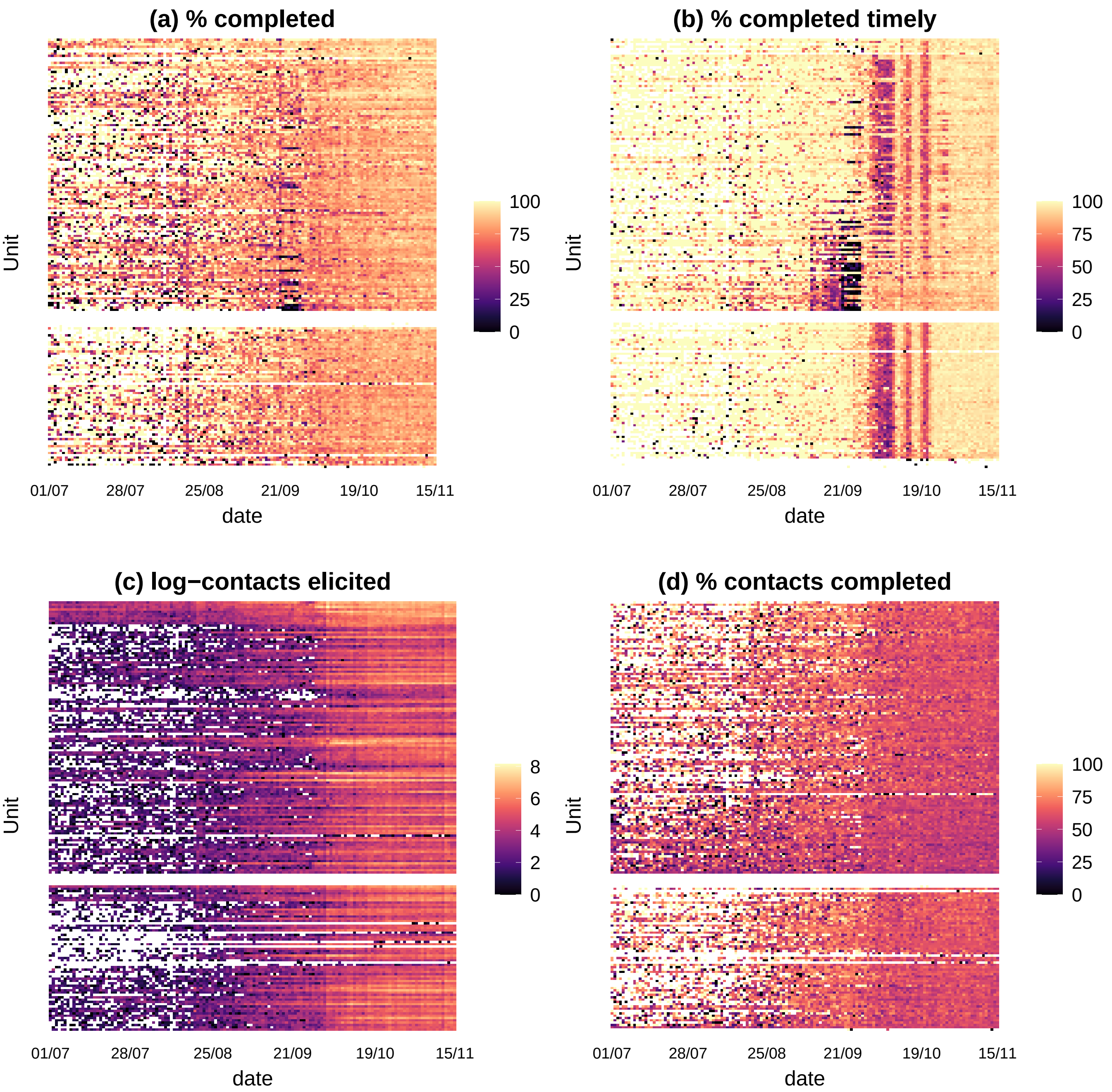

Figure D.1: Graphical summaries of the LTP data introduced in Section 2 of the main paper. Panels (a)–(d) show data on completion, timeliness, number of contacts and contact completion, respectively. Each panel is split into two sub-panels, where the top and bottom sub-panels represent the data on treated and control units, respectively.

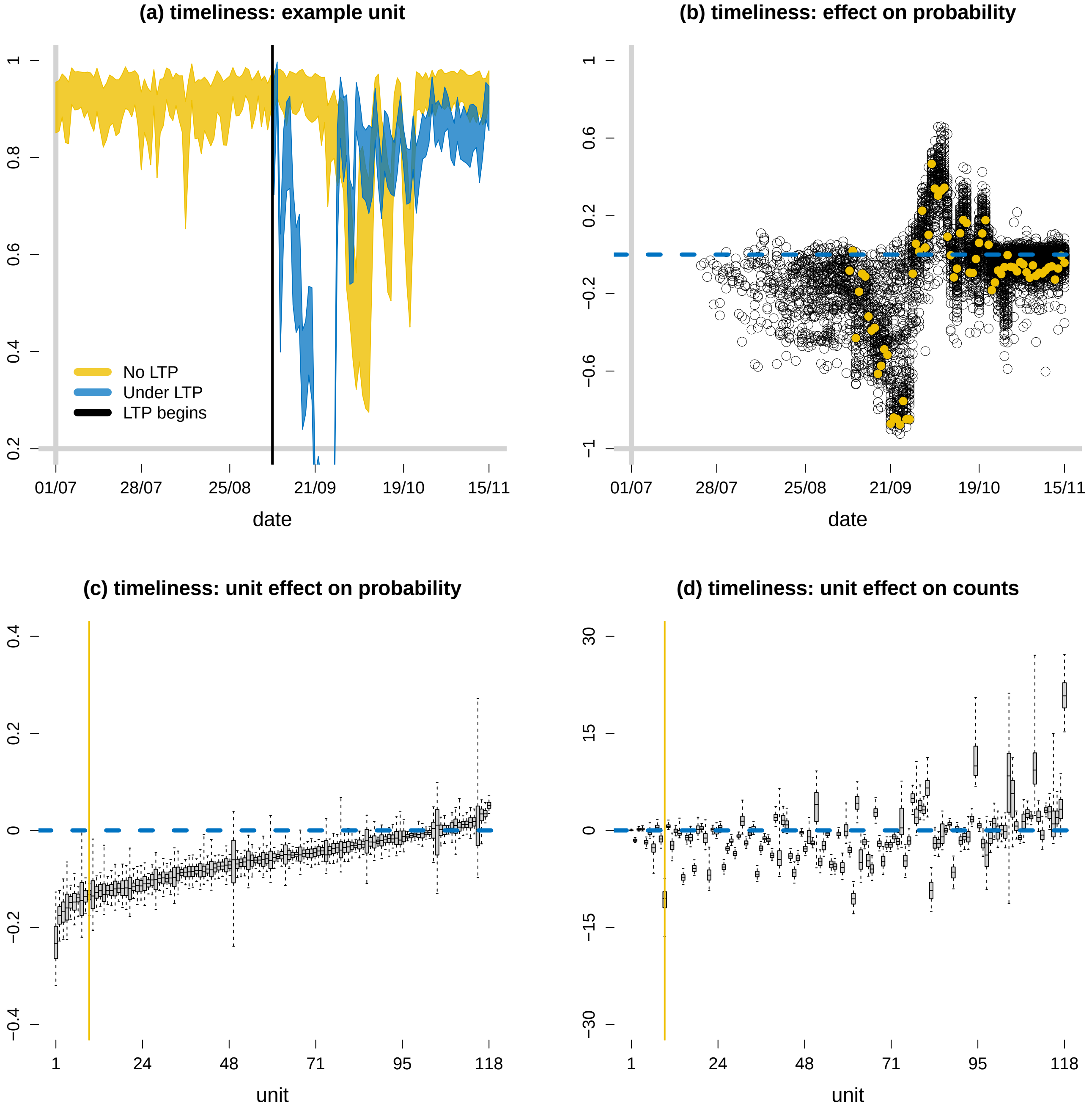

Figure D.2: Summary of the results for outcome **timely completion**. Panel (a) shows the 95% credible bands for $p_{\iota t2}$ and $p_{\iota t2}^{(T_\iota)}$, where ι is a unit chosen for illustration. Panel (b) is the scatterlplot of the point estimates (obtaiend as the posterior mean) of β_{it2} that is, the effect of LTPs on timely completion probability of unit i at day t. Yellow dots represent the $\beta_{\iota t2}$. Panels (c) and (d) show posterior summaries of β_{i2} (average unit effect on timely completion probability) and γ_{i2} (average unit effect on timely completion counts), respectively, where units have been ordered by increasing mean posterior β_{i2} in both plots. The vertical yellow lines are used to indicate unit ι. In panels (c) and (d), the boxes and whiskers represent the 75% and 95% credible intervals, respectively.

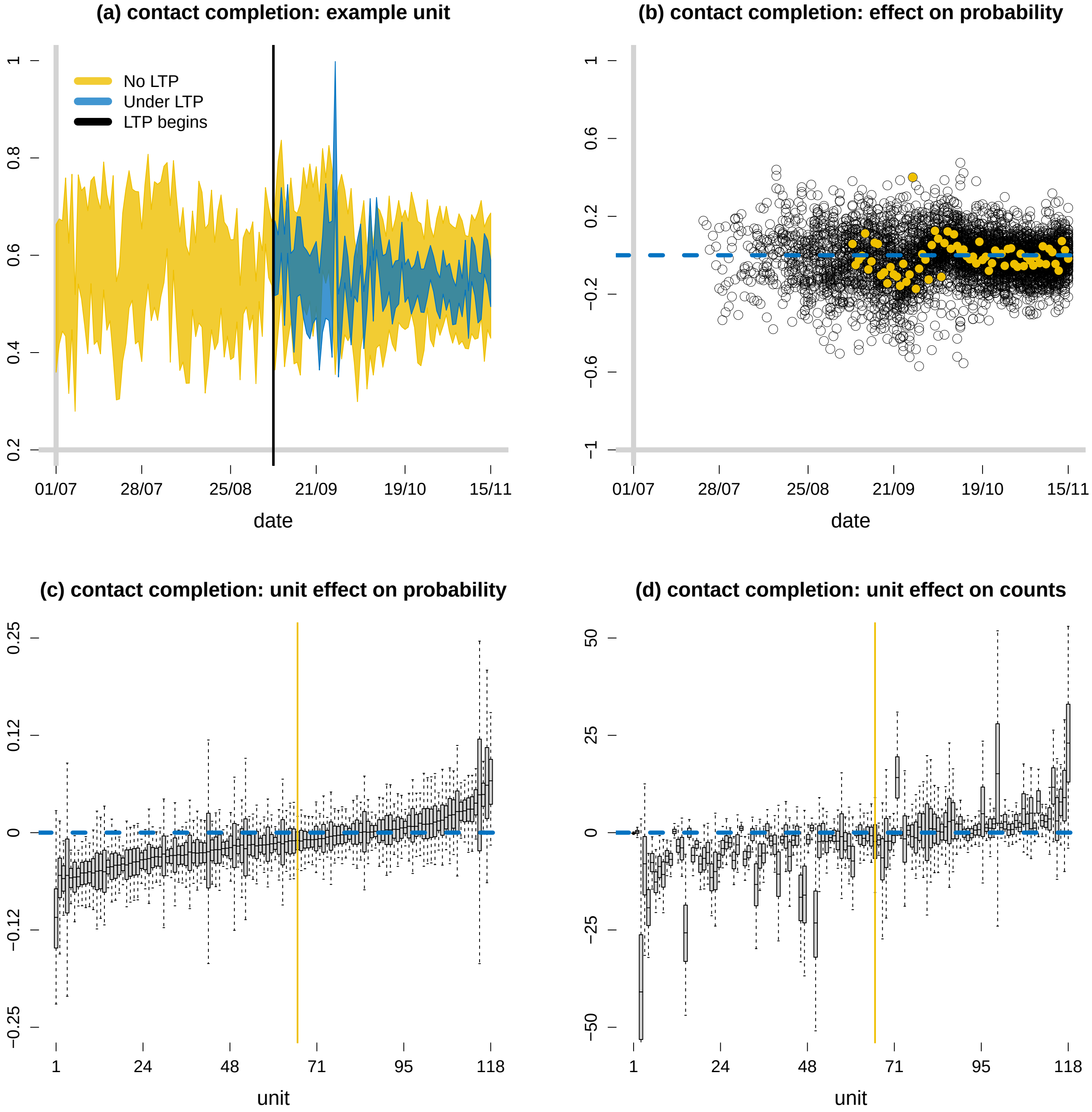

Figure D.3: Summary of the results for outcome **contact completion**. Panel (a) shows the 95% credible bands for $p_{\iota t3}$ and $p_{\iota t3}^{(T_\iota)}$, where ι is a unit chosen for illustration. Panel (b) is the scatterlplot of the point estimates (obtained as the posterior mean) of β_{it3} that is the effect of LTPs on contact completion probability of unit i at day t. Yellow dots represent the $\beta_{\iota t3}$. Panels (c) and (d) show posterior summaries of β_{i3} (average unit effect on contact completion probability) and γ_{i3} (average unit effect on contact completion counts), respectively, where units have been ordered by increasing mean posterior β_{i3} in both plots. The vertical yellow lines are used to indicate unit ι. In panels (c) and (d), the boxes and whiskers represent the 75% and 95% credible intervals, respectively.

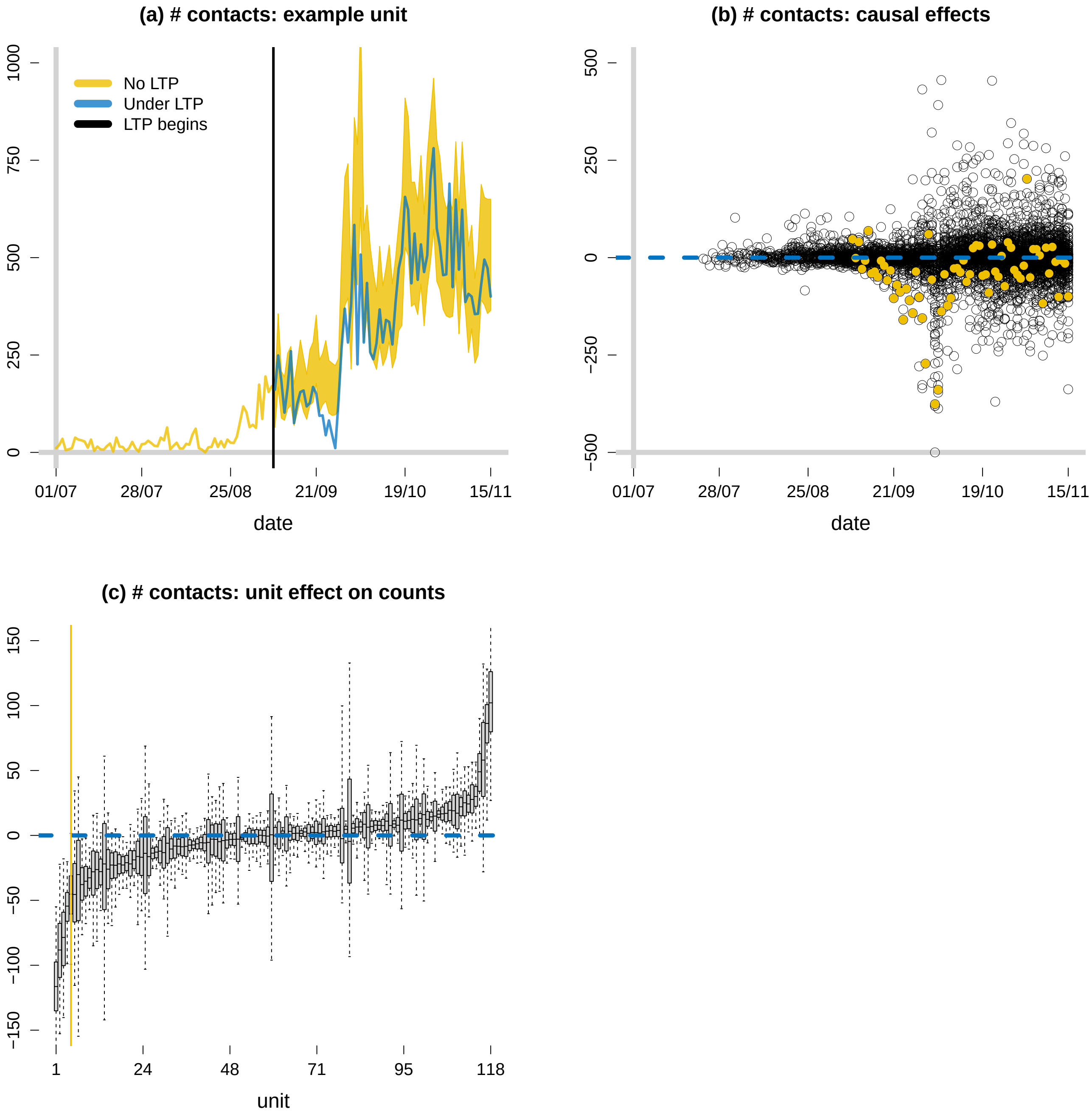

Figure D.4: Summary of the results for outcome **number of contacts**. Panel (a) shows the values of $z_{\iota t1}$ and the 95% credible bands for $z^{(0)}_{\iota t1}$ $(t > T_\iota)$, where ι is a unit chosen for illustration. Panel (b) is the scatterlplot of point estimates (obtained as the posterior mean) of δ_{it1} that is, the effect of LTPs on number of contacts elicited of unit i at day t. Yellow dots represent the $\delta_{\iota t1}$. Panel (c) shows posterior summaries of δ_{i1} (average unit effect on number of contacts), where units have been ordered by increasing mean posterior δ_{i1} in both plots. The vertical yellow lines are used to indicate unit ι. In panel (c), the boxes and whiskers represent the 75% and 95% credible intervals, respectively.

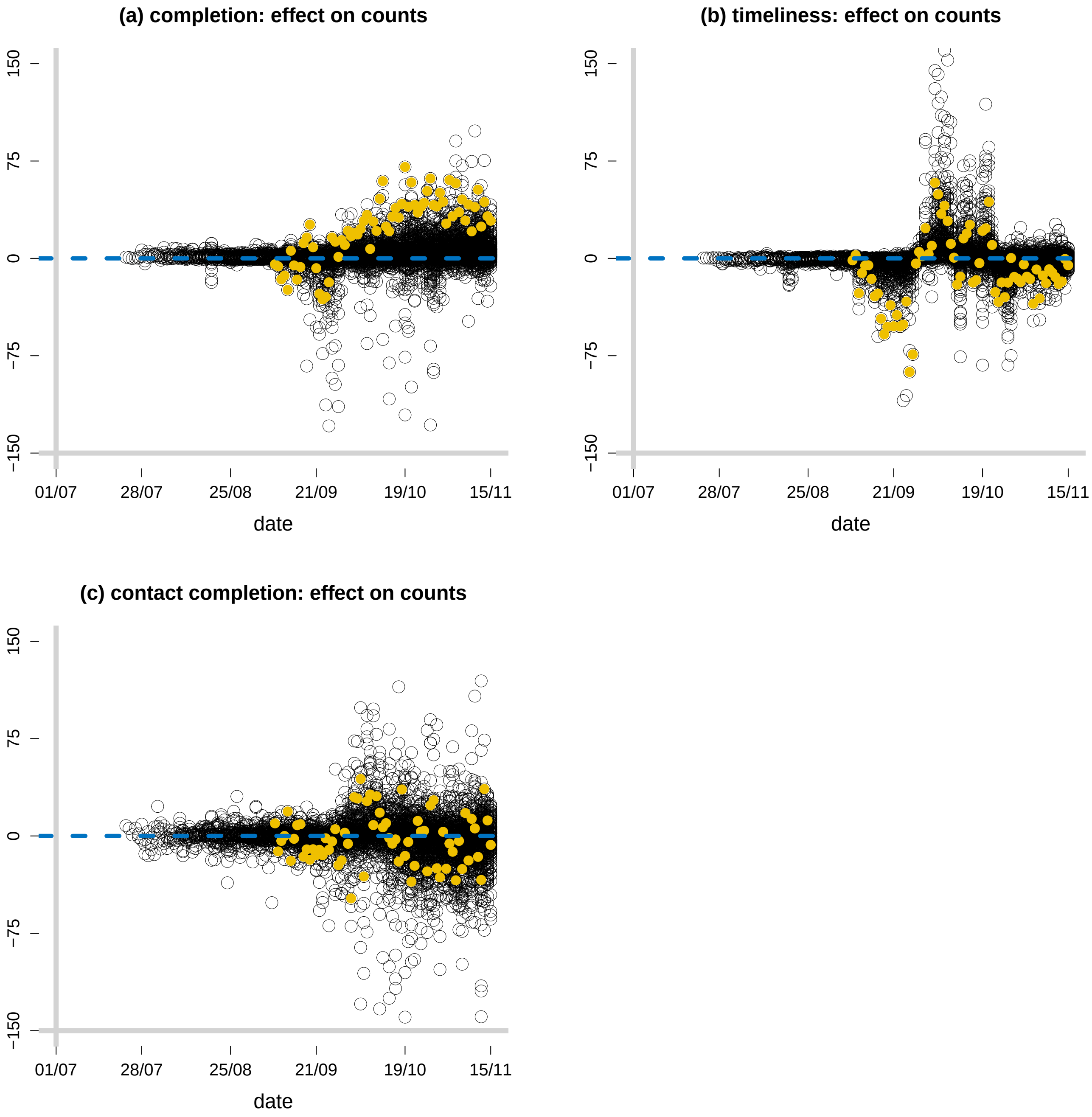

Figure D.5: Scatterplots of the point estimates (obtained as the posterior mean) of γ_{itd}. Panels (a), (b) and (c) correspond to outcomes completion, timely completion and contact completion, respectively. In all plots, the yellow dots show the estimated effects in unit ι that we used in the main paper for illustration.

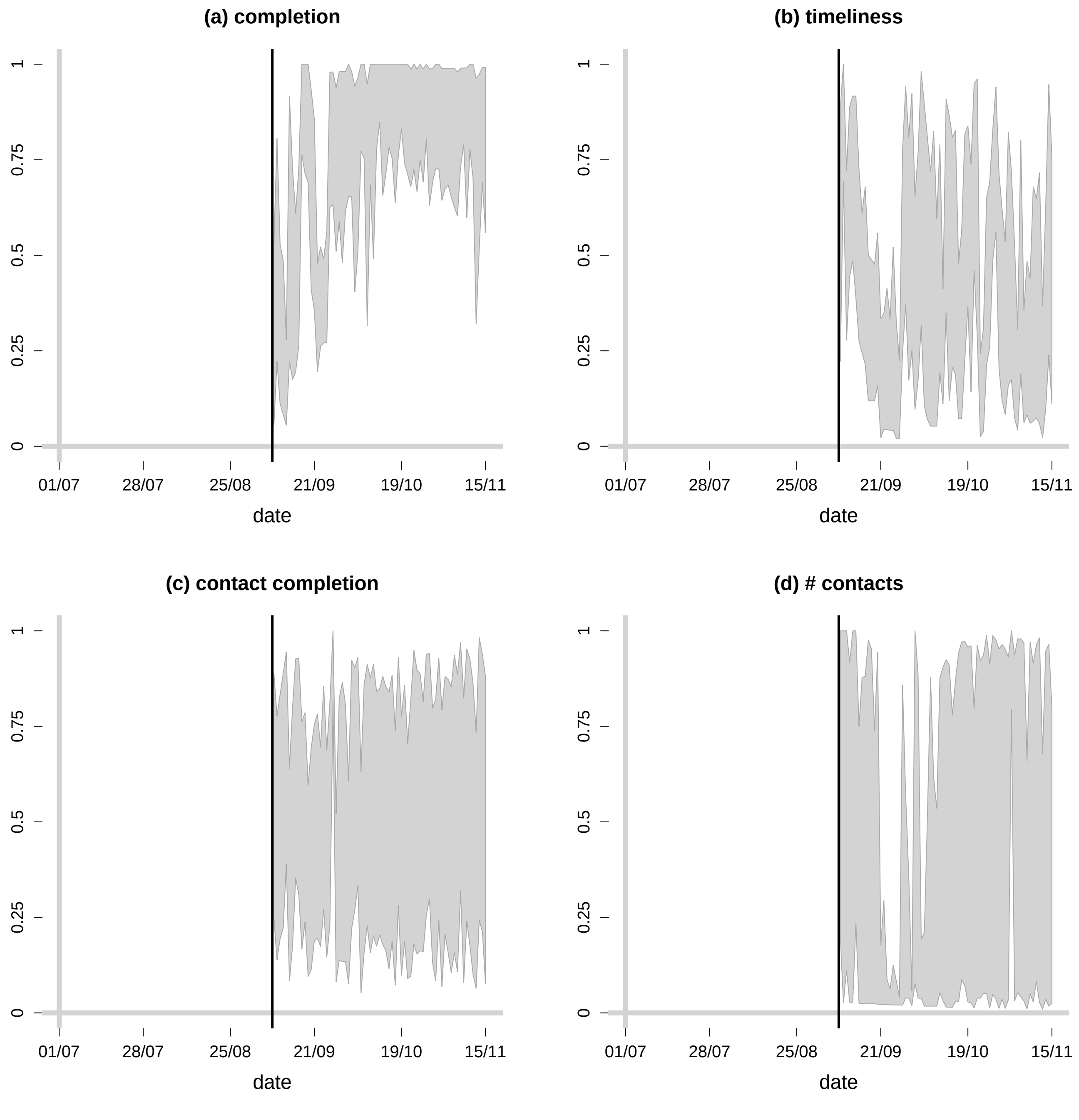

Figure D.6: Illustration of posterior ranks for unit ι. Panels (a)-(d) summarise the posterior distribution of $r^{(\beta)}_{\iota t1}$, $r^{(\beta)}_{\iota t2}$, $r^{(\beta)}_{\iota t3}$ and $r^{(\delta)}_{\iota t1}$, respectively, where shaded regions represent the 95% credible bands.

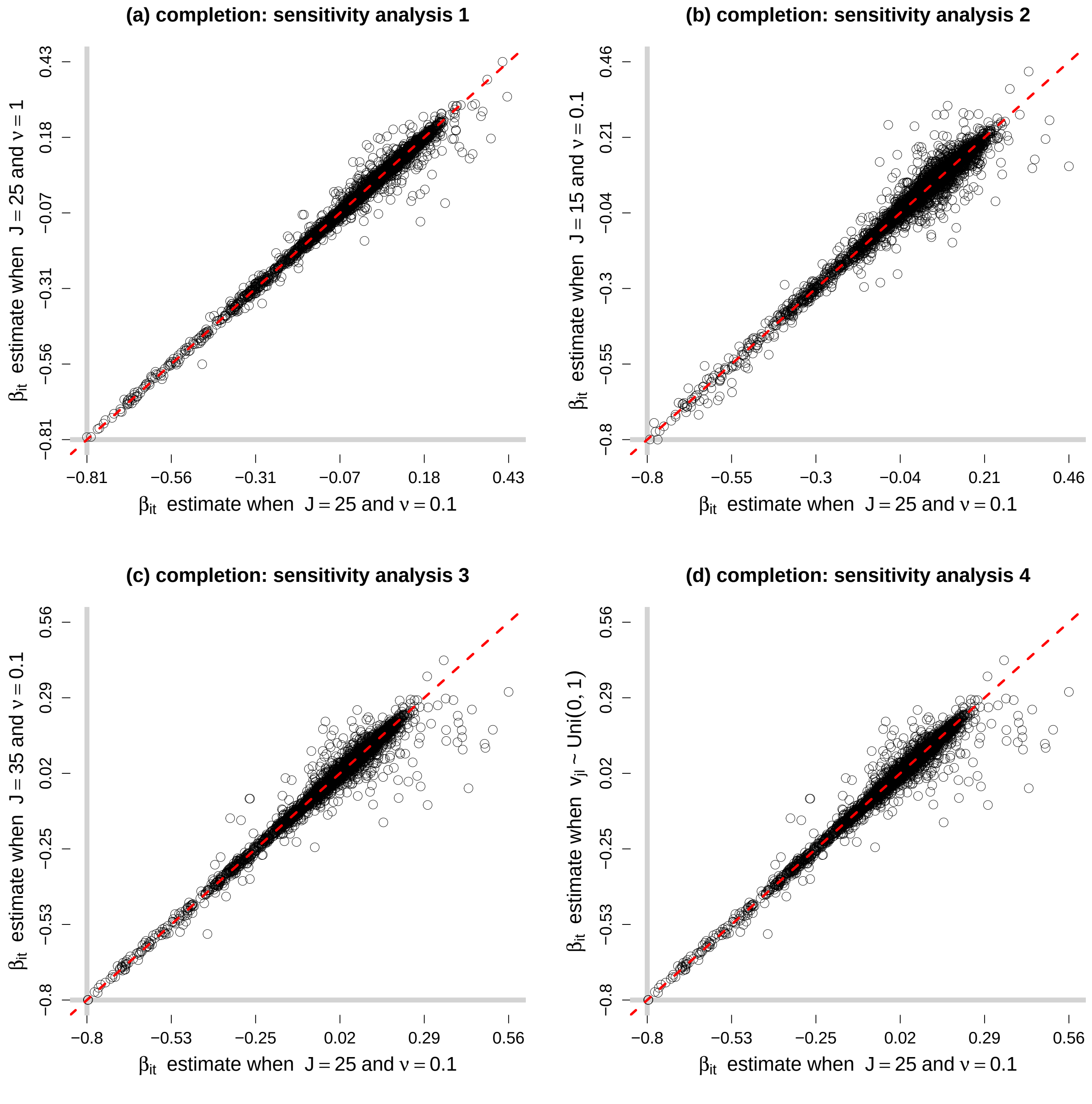

Figure D.7: Results of the sensitivity analysis for estimand β_{it1}. Panels (a)-(d) compare the **point estimates** of β_{it1} obtained in each one of the four sensitivity analyses considered, against the corresponding quantities obtained from our original analysis in Section 6 of the main paper.

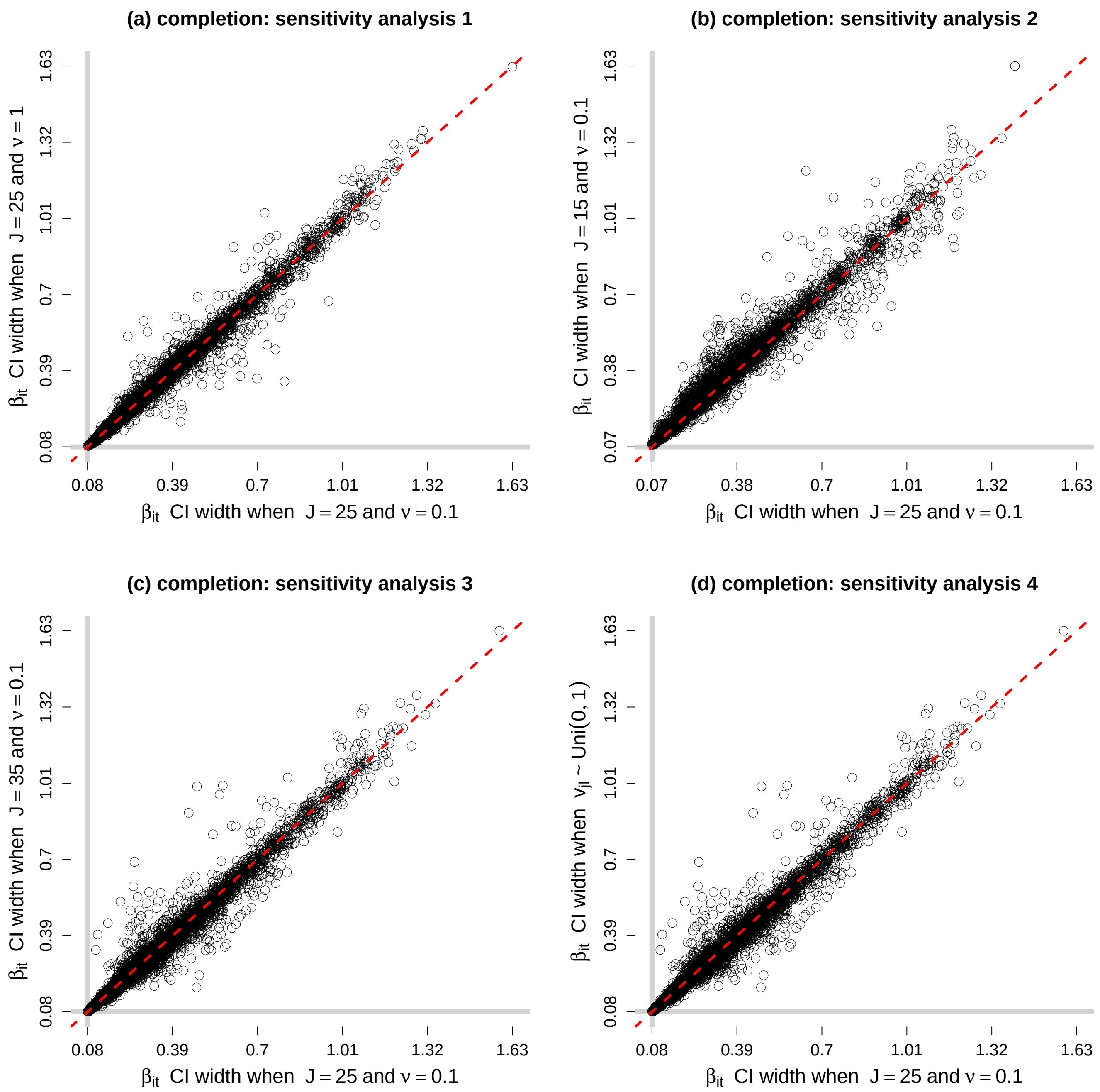

Figure D.8: Results of the sensitivity analysis for estimand β_{it1}. Panels (a)-(d) compare the **credible interval width** of β_{it1} obtained in each one of the four sensitivity analyses considered, against the corresponding quantities obtained from our original analysis in Section 6 of the main paper.

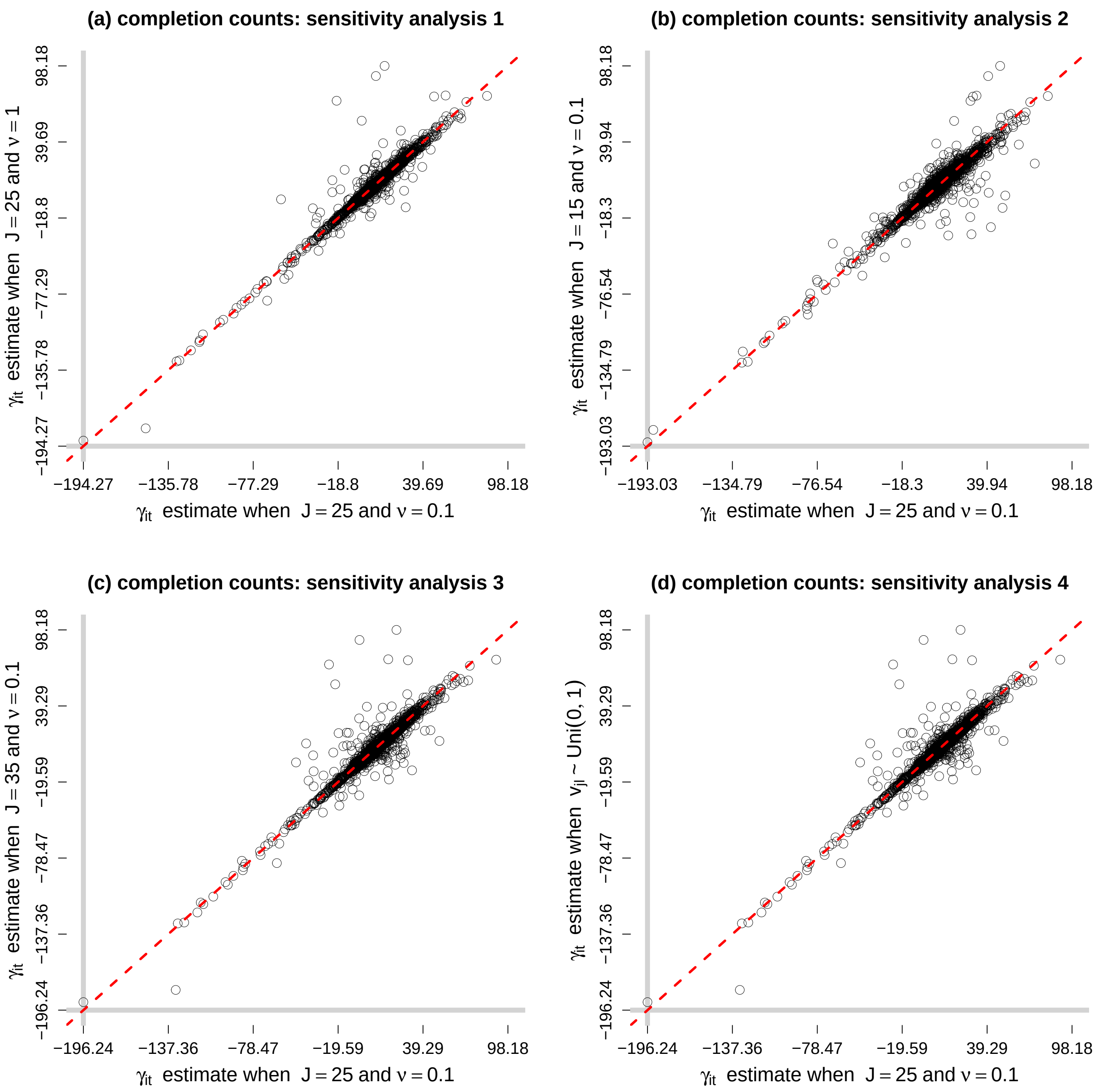

Figure D.9: Results of the sensitivity analysis for estimand γ_{it1}. Panels (a)-(d) compare the **point estimates** of γ_{it1} obtained in each one of the four sensitivity analyses considered, against the corresponding quantities obtained from our original analysis in Section 6 of the main paper.

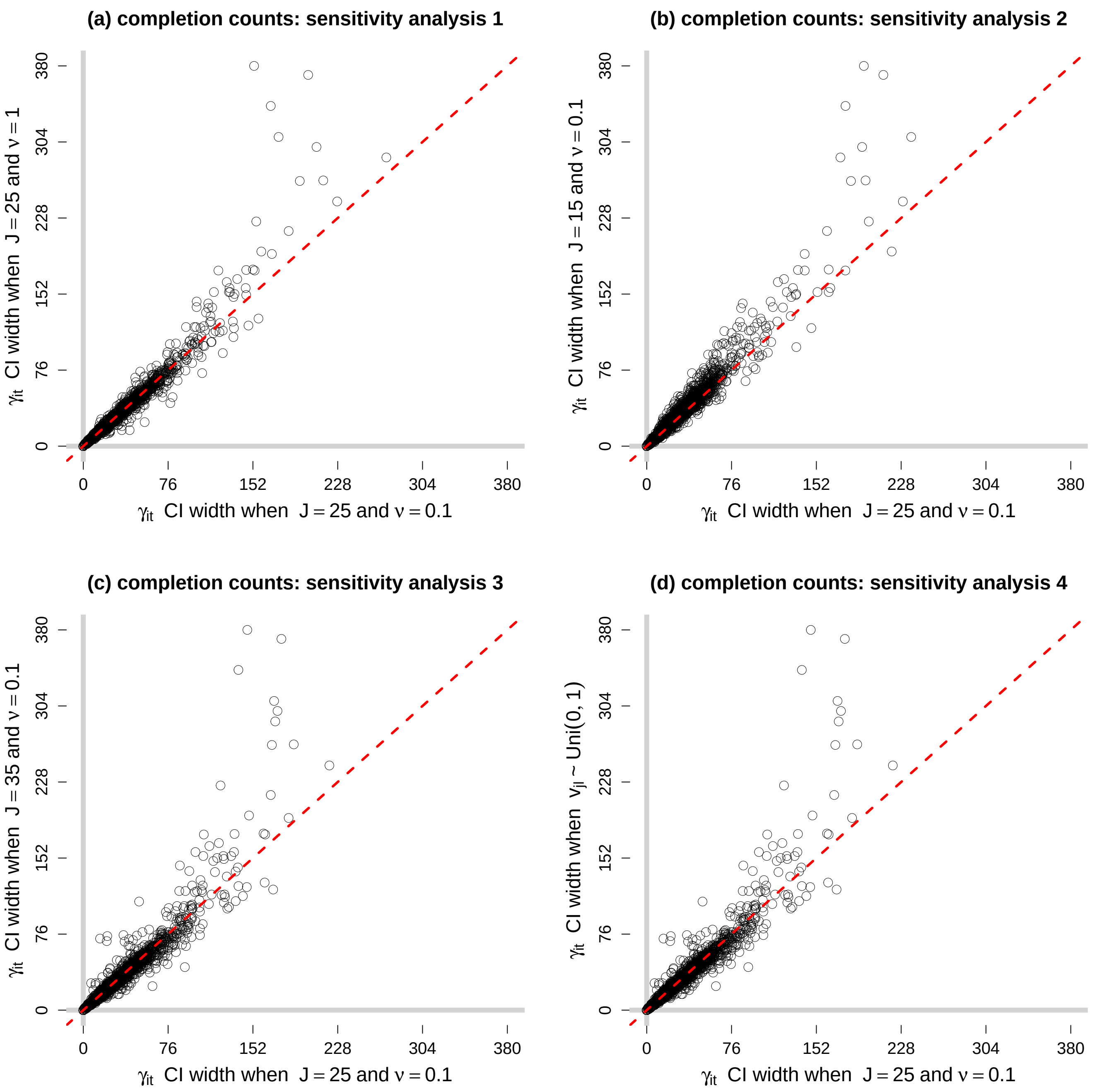

Figure D.10: Results of the sensitivity analysis for estimand γ_{it1}. Panels (a)-(d) compare the **credible interval width** of γ_{it1} obtained in each one of the four sensitivity analyses considered, against the corresponding quantities obtained from our original analysis in Section 6 of the main paper.

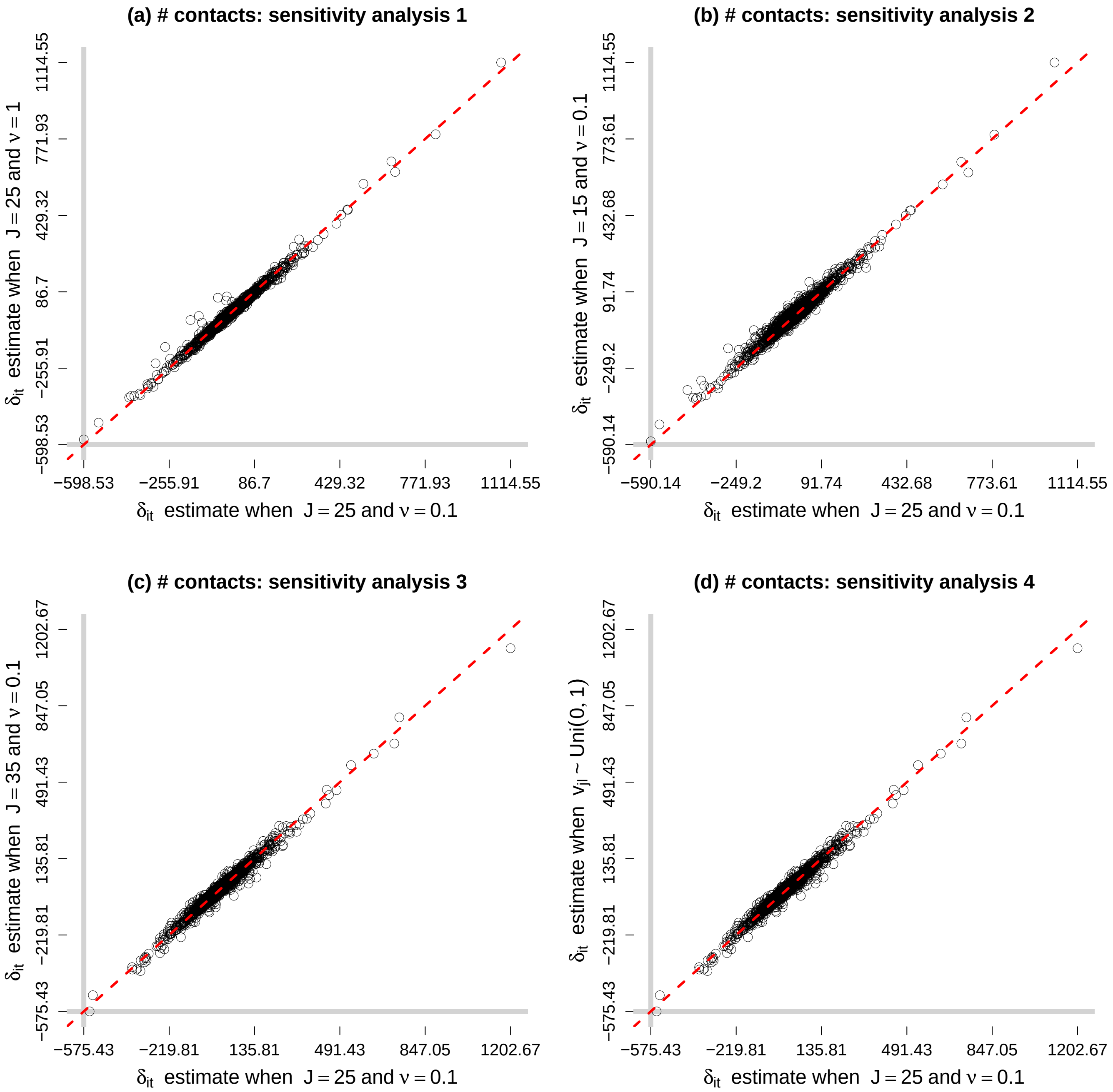

Figure D.11: Results of the sensitivity analysis for estimand δ_{it1}. Panels (a)-(d) compare the **point estimates** of δ_{it1} obtained in each one of the four sensitivity analyses considered, against the corresponding quantities obtained from our original analysis in Section 6 of the main paper.

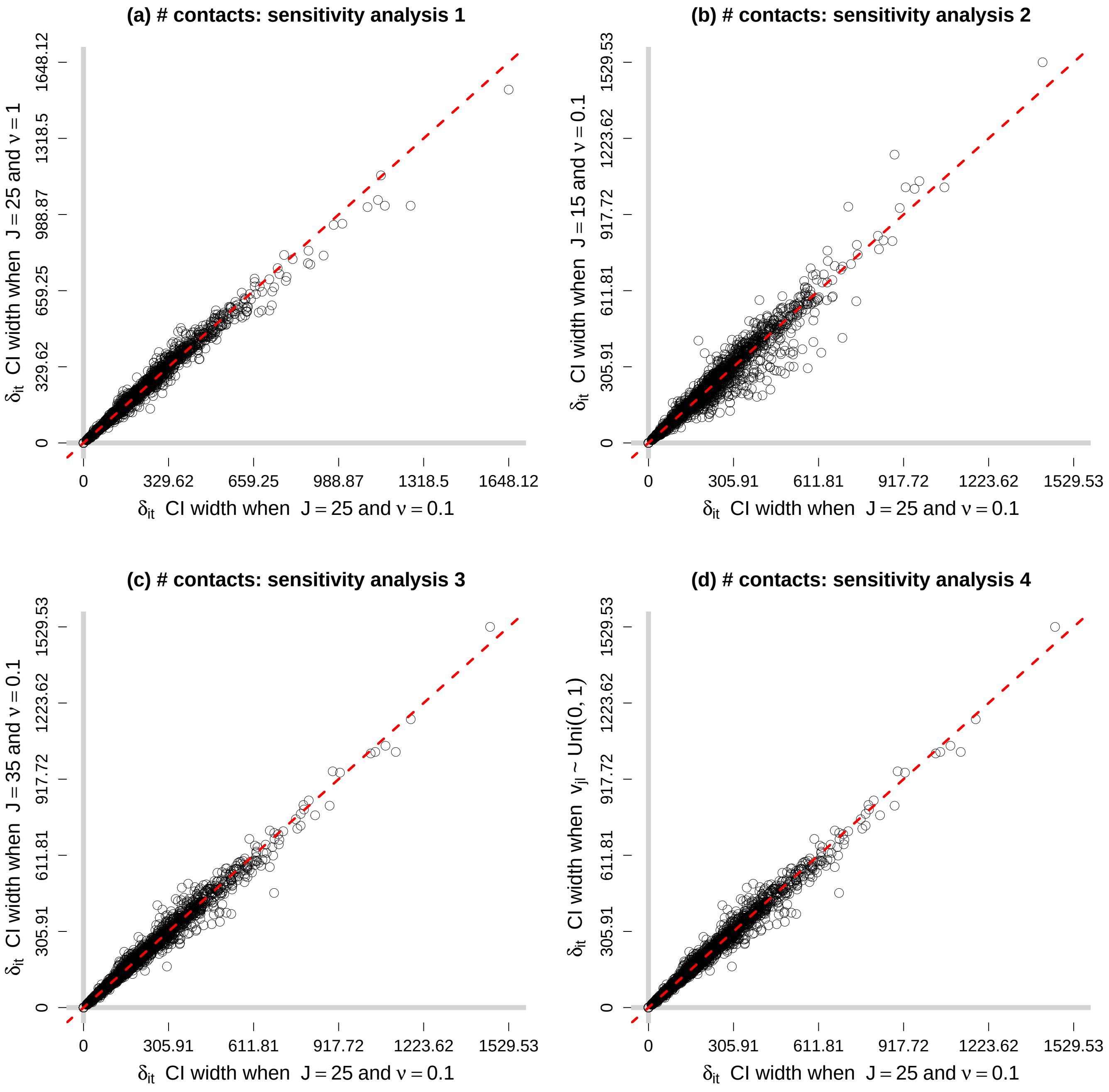

Figure D.12: Results of the sensitivity analysis for estimand δ_{it1}. Panels (a)-(d) compare the **credible interval width** of δ_{it1} obtained in each one of the four sensitivity analyses considered, against the corresponding quantities obtained from our original analysis in Section 6 of the main paper.

References

Gao, C., McDowell, I. C., Zhao, S., Brown, C. D., and Engelhardt, B. E. (2016). Context specific and differential gene co-expression networks via Bayesian biclustering. *PLOS Computational Biology*, 12(7):1–39.

Girolami, M. and Calderhead, B. (2011). Riemann manifold Langevin and Hamiltonian Monte Carlo methods. *Journal of the Royal Statistical Society: Series B (Statistical Methodology)*, 73(2):123–214.

Livingstone, S. and Zanella, G. (2022). The Barker proposal: Combining robustness and efficiency in gradient-based MCMC. *Journal of the Royal Statistical Society Series B: Statistical Methodology*, 84(2):496–523.

Robins, J. M. and Richardson, T. S. (2011). Alternative Graphical Causal Models and the Identification of Direct Effects. In *Causality and Psychopathology: Finding the Determinants of Disorders and their Cures*. Oxford University Press.

Samartsidis, P., Seaman, S., Montagna, S., Charlett, A., Hickman, M., and De Angelis, D. (2020). A Bayesian multivariate factor analysis model for evaluating an intervention by using observational time series data on multiple outcomes. *Journal of the Royal Statistical Society: Series A (Statistics in Society)*, 183(4):1437–1459.

Stensrud, M. J., Young, J. G., Didelez, V., Robins, J. M., and Hernán, M. A. (2022). Separable effects for causal inference in the presence of competing events. *Journal of the American Statistical Association*, 117(537):175–183.
