# Supplementary material for: A Bayesian multivariate factor analysis model for causal inference using time-series observational data on mixed outcomes
Source: Biostatistics. 2023 Dec 6;25(3):867–84. doi: 10.1093/biostatistics/kxad030 (PMC11247182; doi:10.1093/biostatistics/kxad030)
Supplement: kxad030_Supplementary_Data [file kxad030_supplementary_data.zip › biosts-22315-File002.pdf]

A Bayesian multivariate factor analysis model for causal inference using observational time-series data on mixed outcomes

2023-09-27

This vignette provides some R code that can be used to apply the method presented in https://arxiv.org/abs/2110.02005 for policy evaluation. It uses R libraries Rcpp and RcppArmadillo. To report bugs or to ask any questions, please contact ps747@cam.ac.uk.

1) Data pre-processing

The main inputs to the program are three-dimensional arrays y (continuous outcomes), k (binomial number of successes), n (binomial number of trials), z (count outcomes) and w (count outcomes offsets). These arrays should have the same number of rows (representing time-series length T) and the same number of columns (representing the number of units N). The third dimension should be set equal to the total number of outcomes of each type (D_1, D_2 and D_3 in the paper). If your dataset does not have any outcomes of one type, set the z-dimension of the corresponding array to zero. For count outcomes, if there is no offset, set all elements of w equal to one. The code does not currently support missing values.

For illustration, we use a synthetic dataset generated for the purposes of the simulation study of Section 5 of the paper. The following commands load the data in R and prepare the inputs for the program:

```
load('./R/dataset_vignette.RData')
nTimes = dim(y)[1]
nUnits = dim(y)[2]
y = array( y, c(nTimes,nUnits,1) )
z = array( z, c(nTimes,nUnits,1) )
w = array( w, c(nTimes,nUnits,1) )
k = array( k, c(nTimes,nUnits,1) )
n = array( n, c(nTimes,nUnits,1) )
```

The other main input is the vector Timings of size N, specifying the intervention times. For each unit, set the corresponding element of Treat to be equal to the number of pre-intervention observations plus one (T_i in the paper). Note that for control units, this will be $T+1$. Treat should have no entries equal to zero (i.e. units who are under intervention throughout the entire study period) and at least one entry equal to $T+1$ (i.e. at least one control unit).

2) Fitting the model

The function to fit the model is cbfa (for causal Bayesian factor analysis). You can load this in R using the following commands:

```
library(Rcpp)
library(RcppArmadillo)
sourceCpp('./src/cbfa.cpp')
```

As well as the arrays of data, the following inputs have to be specified:

1. nMCMC: The total number of MCMC iterations.

2. `nBurn`: A number specifying how many of the `nMCMC` iterations should be viewed as burn-in. The Barker and SMMALA stepsizes are adapted during this phase. Posterior samples are still saved.
3. `nThin`: Every how many iterations to save posterior draws. Make sure this number divides `nMCMC` exactly.
4. `window`: When adapting the Barker and SMMALA stepsizes, the acceptance rate is evaluated based on the last `window` iterations.
5. `P`: The upper limit for the number of factors (J^* in the paper).
6. `SMAX`: The upper limit of the uniform prior on normal outcome variances.
7. `tpb_prior`: Vector of length seven, specifying the hyperparameters of the TPB shrinkage prior. We recommend setting to `(rep(0.5,6),0.1)`. The last argument is the global shrinkage parameter (ν in the paper).
8. `tpb_nBurn`: A small number (500 will suffice) of iterations at the beginning of the simulation, during which the loadings shrinkage parameters are not updated. We do this to allow the algorithm to move to a region with high posterior density.

In our simulation study, we used the following specifications:

```
# Simulation parameters
nBurn     = 500
nIter     = 1000      # not an input, specifying number of post-burn-in samples in output
nThin     = 25
nMCMC     = nThin*(nBurn+nIter)
window    = 100
P         = 20
SMAX      = 10
tpb       = rep(0.5,7)
tpb[7]    = 0.1       # the \nu parameter
tpb_nBurn = 100
```

We now run the model:

```
# Set the seed to be able to replicate results
set.seed(123)
fit = cbfa( nMCMC=nMCMC, nBurn=nBurn*nThin, nThin=nThin, window=100, P=P, y=y, n=n, k=k,
            w=w, z=z, Timings=Timings, SMAX=10, tpb_prior=tpb, tpb_nBurn=1000 )
```

Depending on the values of N, T, J and D_2, the algorithm might take a very long time to run. Importantly, datasets with multiple binomial variables and large counts will run very slowly, due to the need to draw the PG latent variables (ω_{itd} in the paper). Reducing `nThin` is not recommended as mixing of the over-dispersion parameters (ξ_d) can be slow. If your dataset contains no count variables, or if you are happy to use the size/probability parametrisation of the negative binomial distribution (as opposed to mean-overdispersion used in the paper), all the updates are conjugate. Use the function `cbfa_conjugate` instead. If you are trying out the commands in this vignette, there is no need to wait for the MCMC to run, you can use the object stored in `fit_vignette.RData`.

3) Diagnostics and visualisation

Type `names(fit)` to see the variables for which posterior samples are saved. Note that when there are multiple outcomes of one type, the corresponding factors are stacked one next to the other in `fit$f_`. For instance, if there were $D_1 = 2$ continuous outcomes, each `fit$f_normal[,,b]` (`b` is MCMC iterations) would have `2*P` columns. Draw some traceplots to make sure the chain is mixing, eg:

```
load( './R/fit_vignette.RData' )
par( mfrow=c(2,2) )
plot( fit$gamma[-c(1:nBurn)],    ylab='', xlab='Iteration', main='TPB gamma parameter', type='l')
plot( fit$xi[-c(1:nBurn),1],     ylab='', xlab='Iteration', main='NB dispersion',       type='l')
```

```
plot( fit$omega[-c(1:nBurn),1], ylab='', xlab='Iteration', main='A PG variable',       type='l')
plot( fit$crt[-c(1:nBurn),1],   ylab='', xlab='Iteration', main='A CRT variable',      type='l')
```

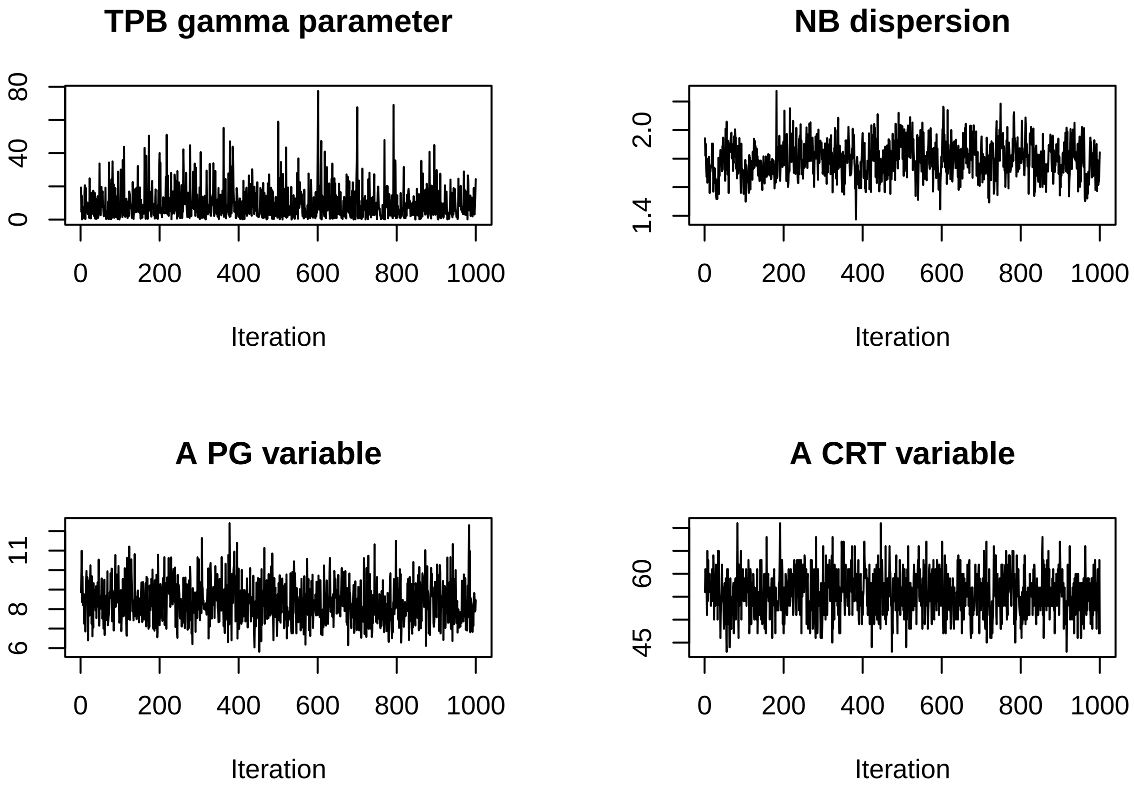

We do not save the posterior samples for all PG (`fit$omega`) and CRT (`fit$crt`) auxiliary variables. Instead, we randomly choose a few of them for the purposes of diagnostics. The observations to which these correspond (i.e. the i, t and d indices), are given by `fit$omega_idx` and `fit$crt_idx`, respectively.

To get an idea of how many factors are supported by the data, have a look at some draws from the posterior of the loadings matrix, e.g.:

```
library(lattice)
nDraws = length( fit$eta )
levelplot( t(fit$L[,,nDraws]), asp=1, ylab='Unit', xlab='Factor' )
```

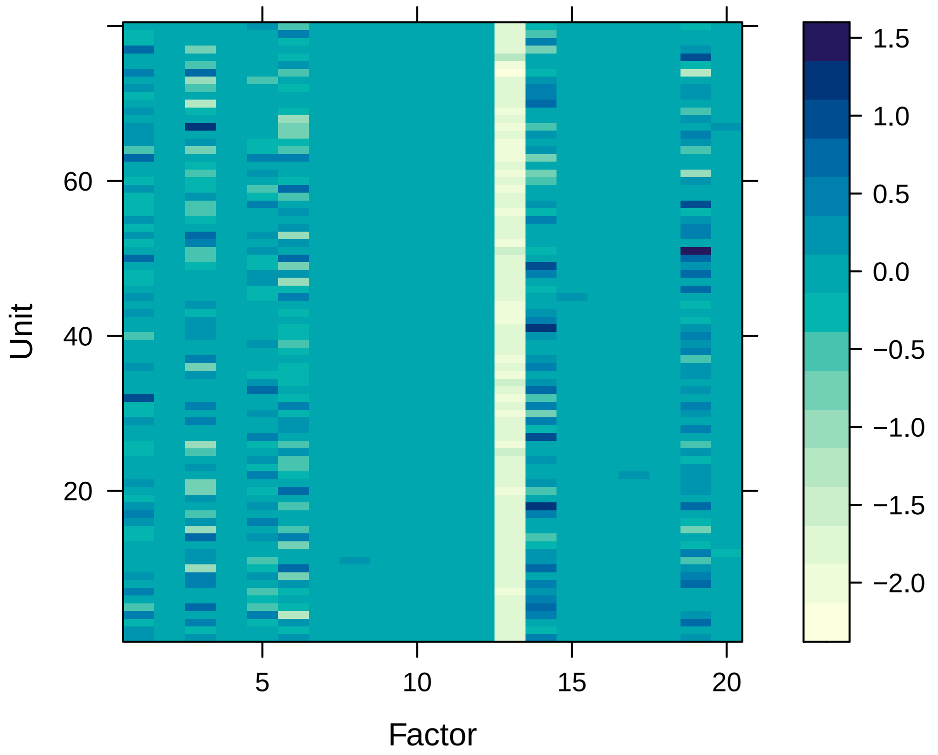

We see that only seven columns of the loadings matrix are dense (i.e. the elements are non-zero for most units). In this case, these are columns 1, 3, 5, 6, 13, 14 and 19. This is expected as we set $J = 6$ in our simulation study, and one additional factor is needed to account for the mean being different to zero. One can also look at posterior of the L1-norm of the columns of Λ:

```
L1_norm = matrix( NA, nDraws, P )
for ( b in 1:nDraws ) {
  L1_norm[b,] = apply( abs( fit$L[,,b] ), 2, mean )
}
# Function boxes in plot.R can help us visualize these posteriors
source('./R/plot.R')
boxes( L1_norm[-c(1:nBurn),], outliers=FALSE, MAIN='L1 posterior norm', XLAB='Factor' )
```

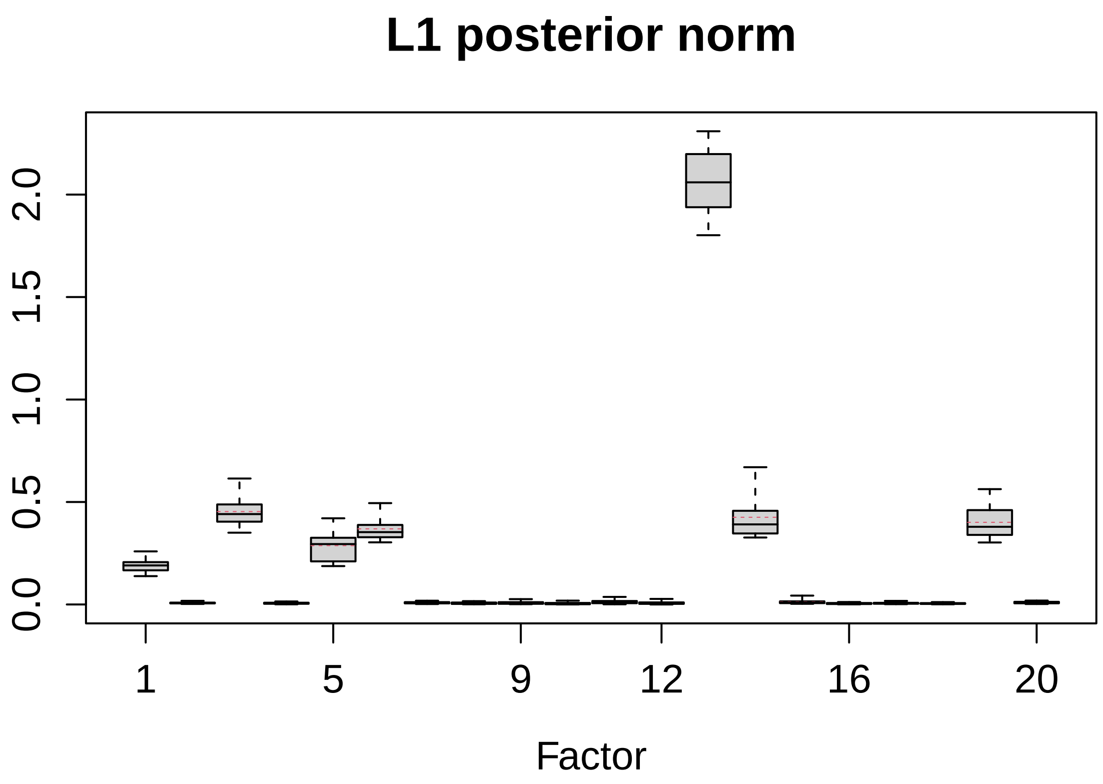

The object `fit` does not currently provide draws from the posterior distribution of the causal effects or the fitted values. This is because, depending on the size of the dataset, these might occupy a large amount of memory. However, one can obtain them for a single unit using the function `fitted_values` in `post_processing.R`. For example, for unit $i = 3$, we type:

```
id = 3
source('./R/post_processing.R')
unit3 = fitted_values( id, fit, Timings, y, n, k, z, w )
```

The function `polygons` in `plot.R` can help us visualise the outputs. Firstly, for units $i = 3$ and the first outcome $d = 1$ of each type, we plot the 95% credible bands (observed data overlayed in red) for μ_{itd}, p_{itd}, $k^{(0)}_{itd}$ and $z^{(0)}_{itd}$ by typing:

```
d = 1
par( mfrow=c(2,2) )
polygons( unit3$fit_normal[-c(1:nBurn),,d], MAIN='Normal outcome',  XLAB='Time point' )
lines( y[,id,d], col=2, lty=2, lwd=2 )
polygons( unit3$fit_binom_prob[-c(1:nBurn),,d], MAIN='Binomial probability',  XLAB='Time point' )
lines( k[,id,d]/n[,id,d], col=2, lty=2, lwd=2 )
polygons( unit3$fit_binom_counts[-c(1:nBurn),,d], MAIN='Binomial counts',  XLAB='Time point' )
lines( k[,id,d], col=2, lty=2, lwd=2 )
polygons( unit3$fit_negbin[-c(1:nBurn),,d], MAIN='Count outcome',  XLAB='Time point' )
lines( z[,id,d], col=2, lty=2, lwd=2 )
```

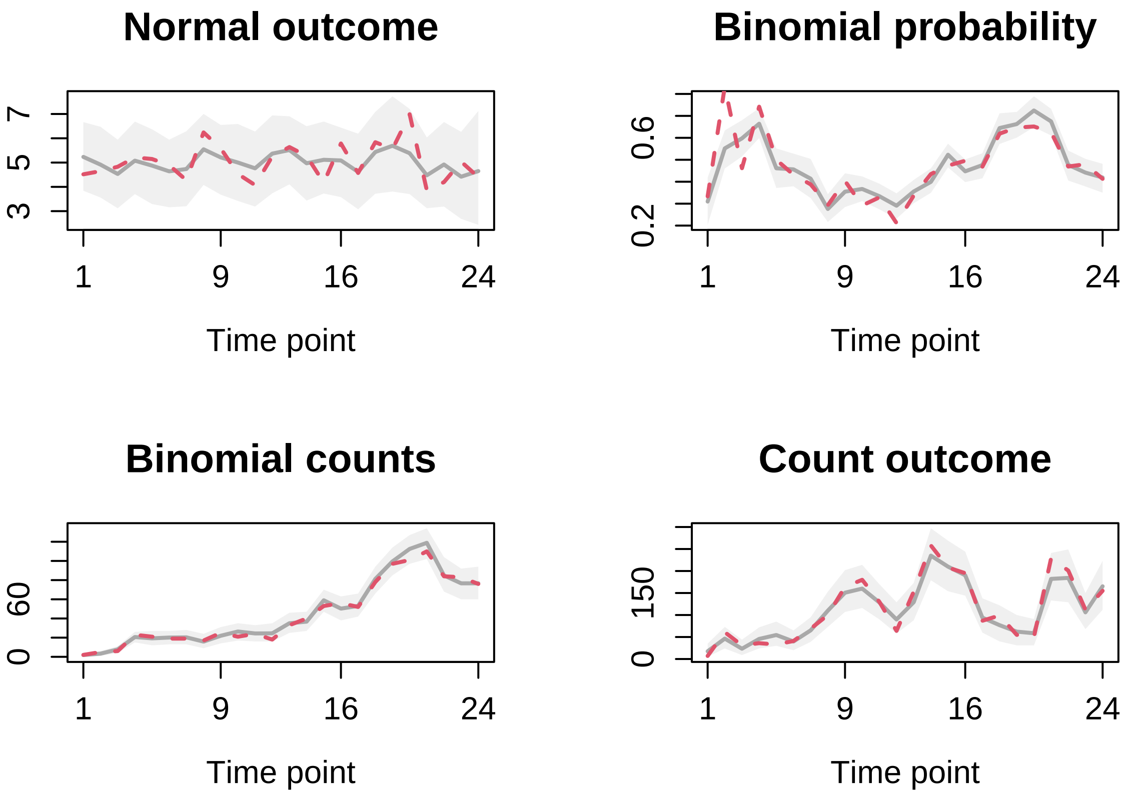

Secondly, we plot 95% credible bands for the causal effects α_{itd}, β_{itd}, γ_{itd} and δ_{itd} defined in Section 3 of the paper. For $i = 3$ and $d = 1$, this can be done by typing:

```
d = 1
par( mfrow=c(2,2) )
polygons( unit3$alpha[-c(1:nBurn),,d], MAIN='Effect on continuous outcome',  XLAB='Time point' )
abline( h=0, col=1, lty=2 ); abline( v=Timings[id]-0.5, col=3, lty=2 )
polygons( unit3$beta_prob[-c(1:nBurn),,d], MAIN='Effect on binomial probability',  XLAB='Time point' )
abline( h=0, col=1, lty=2 ); abline( v=Timings[id]-0.5, col=3, lty=2 )
polygons( unit3$beta_counts[-c(1:nBurn),,d], MAIN='Effect on binomial counts',  XLAB='Time point' )
abline( h=0, col=1, lty=2 ); abline( v=Timings[id]-0.5, col=3, lty=2 )
polygons( unit3$delta[-c(1:nBurn),,d], MAIN='Effect on count outcome',  XLAB='Time point' )
abline( h=0, col=1, lty=2 ); abline( v=Timings[id]-0.5, col=3, lty=2 )
```

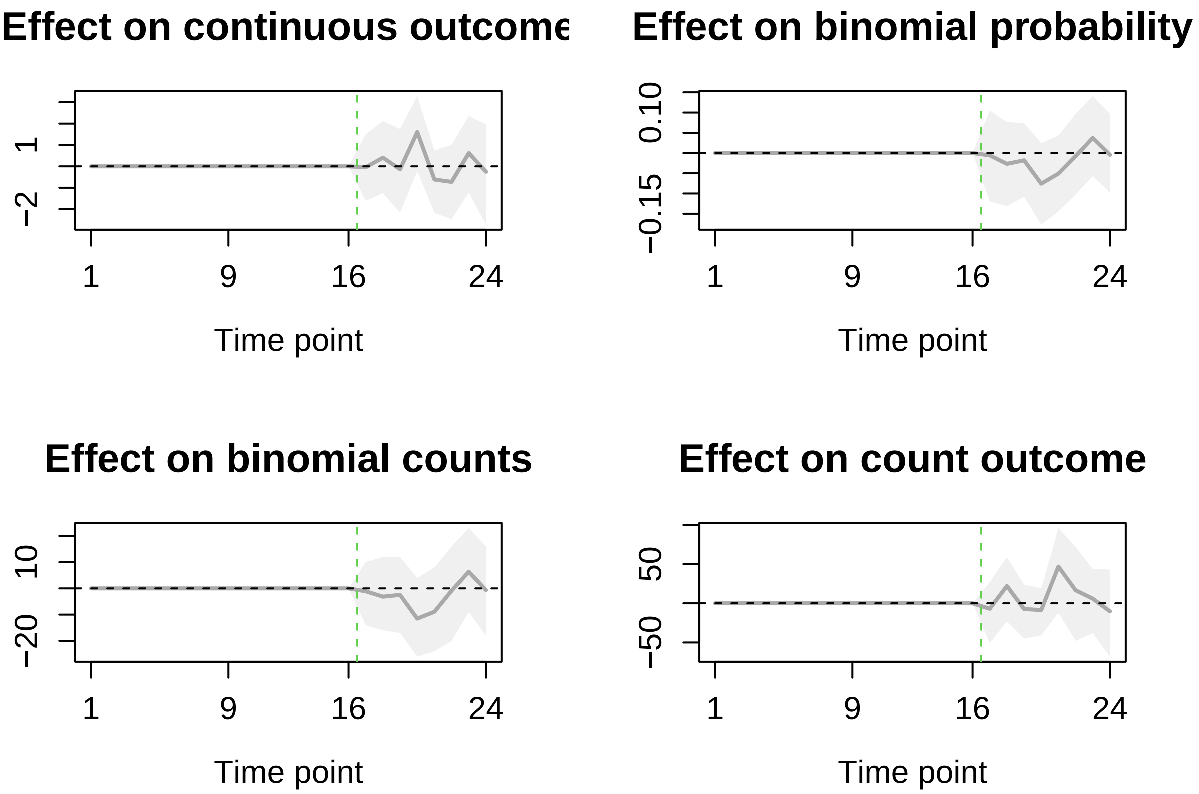

In plots above, the 95% credible bands for the causal effects include zero (i.e. not intervention effect). This is not surprising, because, in this simulation, we set their true values equal to zero. Average treatment effects, such as the ones defined in Section 3 of the paper, can be obtained by transforming the outputs of function `fitted_values` (possibly applied to more than one units). For example, we can obtain the posterior distribution of the average across post-intervention time points effect on unit i and outcome $d = 1$ that is $\alpha_{id} = \frac{1}{T-T_i+1}\sum_{t=T_i}^{T}\alpha_{itd}$. In our example, we can type:

```
avg_effect = apply( unit3$alpha[ -c(1:nBurn),-c(1:(Timings[id]-1)) ,1 ] , 1, mean )
hist( avg_effect, xlab='Average across post-intervention time points effect in unit 3', main='Posterior
```

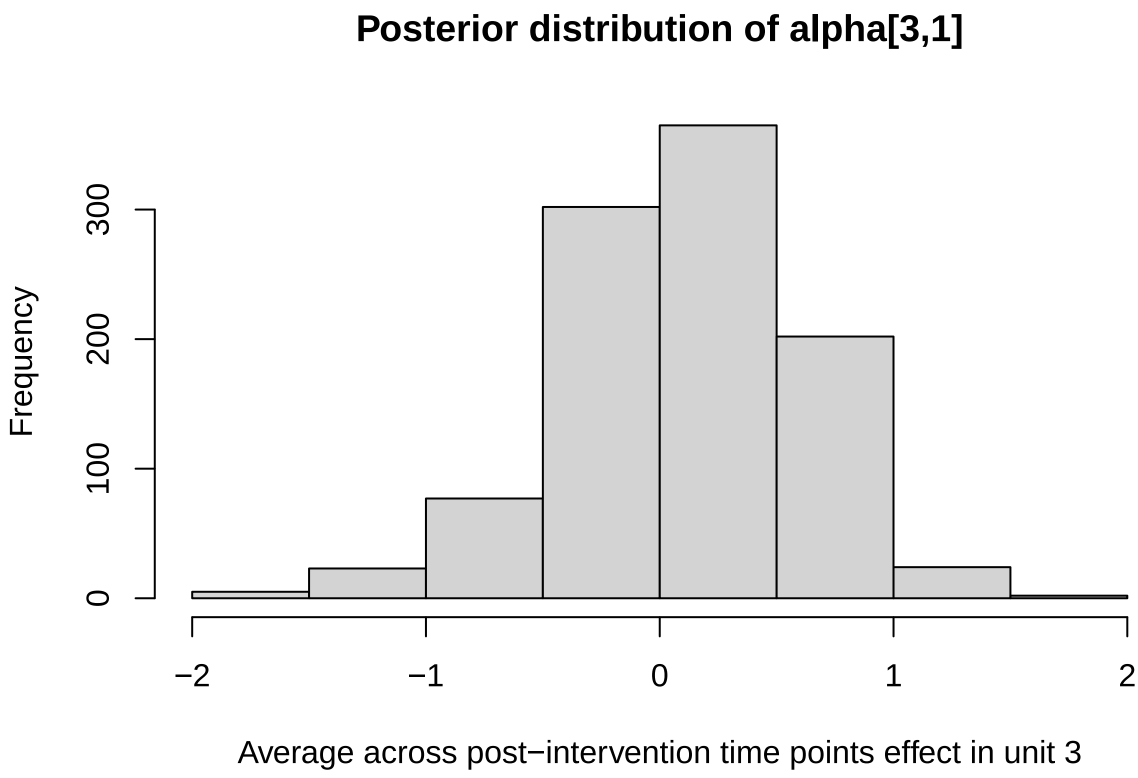

4) Under development

The following feature will be added to the function shortly:

1. Parallel implementation to speed-up running time
2. Alternative MCMC algorithm with different blocking of parameters for better mixing
3. Modelling of Poisson and non-negative continuous outcomes
